# The Genome of *Spironucleus salmonicida* Highlights a Fish Pathogen Adapted to Fluctuating Environments

**DOI:** 10.1371/journal.pgen.1004053

**Published:** 2014-02-06

**Authors:** Feifei Xu, Jon Jerlström-Hultqvist, Elin Einarsson, Ásgeir Ástvaldsson, Staffan G. Svärd, Jan O. Andersson

**Affiliations:** Department of Cell and Molecular Biology, Science for Life Laboratory, Uppsala University, BMC, Uppsala, Sweden; Duke University Medical Center, United States of America

## Abstract

*Spironucleus salmonicida* causes systemic infections in salmonid fish. It belongs to the group diplomonads, binucleated heterotrophic flagellates adapted to micro-aerobic environments. Recently we identified energy-producing hydrogenosomes in *S. salmonicida*. Here we present a genome analysis of the fish parasite with a focus on the comparison to the more studied diplomonad *Giardia intestinalis*. We annotated 8067 protein coding genes in the ∼12.9 Mbp *S. salmonicida* genome. Unlike *G. intestinalis*, promoter-like motifs were found upstream of genes which are correlated with gene expression, suggesting a more elaborate transcriptional regulation. *S. salmonicida* can utilise more carbohydrates as energy sources, has an extended amino acid and sulfur metabolism, and more enzymes involved in scavenging of reactive oxygen species compared to *G. intestinalis*. Both genomes have large families of cysteine-rich membrane proteins. A cluster analysis indicated large divergence of these families in the two diplomonads. Nevertheless, one of *S. salmonicida* cysteine-rich proteins was localised to the plasma membrane similar to *G. intestinalis* variant-surface proteins. We identified *S. salmonicida* homologs to cyst wall proteins and showed that one of these is functional when expressed in *Giardia*. This suggests that the fish parasite is transmitted as a cyst between hosts. The extended metabolic repertoire and more extensive gene regulation compared to *G. intestinalis* suggest that the fish parasite is more adapted to cope with environmental fluctuations. Our genome analyses indicate that *S. salmonicida* is a well-adapted pathogen that can colonize different sites in the host.

## Author Summary

Studies of model organisms are very powerful. However, to appreciate the enormous diversity of genetic and cell biological processes we need to extend the number of available model organisms. For example, there are very few model organisms for diverse microbial eukaryotes, a group of organisms which indeed represents the vast majority of the eukaryotic diversity. To this end, we have developed a system to do genetic modification on the Atlantic salmon pathogen *Spironucleus salmonicida*. Using this system we could show that the organism is capable of producing hydrogen within specialised compartments. Here we present the genome sequence of *S. salmonicida* together with a thorough annotation. We compare the results with the closest available model organism, the human intestinal parasite *Giardia intestinalis*. The fish parasite has a more elaborate system for regulation of gene expression, as well as a larger metabolic capacity. This indicates that *S. salmonicida* is a well-adapted pathogen that can deal with fluctuating environments, an important trait to be able to establish systemic infections in the host. The development of *S. salmonicida* into a model system will benefit the studies of fish infections, as well as cell biological processes.

## Introduction

Most of the eukaryotic diversity is represented by microbial organisms [1]. Yet, the eukaryotic genomic efforts are very biased because only a minority of the microbial groups have been sampled on the genomic level, whereas there is a multitude of animal, plant and fungi genome projects. To understand the true diversity of eukaryotes we need to study the whole eukaryotic diversity. In this study we explore one previously understudied eukaryotic group, the diplomonads, in order to understand the diversity within the group and broaden the knowledge of eukaryotes in general.

Diplomonads are a group of anaerobic, flagellated protists, classified within Fornicata in the supergroup Excavata [1]. They lack aerobic mitochondria [2], although reduced mitochondria (mitosomes) have been identified in the most studied diplomonad *Giardia intestinalis* [3], and recently we identified hydrogenosomes in *Spironucleus salmonicida* [4], the focus of this study. Diplomonads have two diploid nuclei and most likely a sexual or parasexual life cycle [5]–[7], and there is an on-going metabolic adaptation by acquisition of mainly prokaryotic genes [8]–[10]. Together these findings refute earlier suggestions that diplomonads represent a primitive bacterial-like eukaryotic group [11]. There are free-living members of diplomonads, such as *Trepomonas*, as well as commensals or parasites of various animals [2]. For example, *G. intestinalis* causes diarrhea in humans and other animals [12], [13] and members of the genus *Spironucleus* can cause severe infections in ornamental and farmed fish [14]. Diplomonads with different life-styles are intermixed in the diplomonad phylogeny, even within *Spironucleus*, suggesting that transitions between lifestyles have happened multiple times in the group [15].

Aquaculture is a fast growing food sector in the world. The diplomonad *S. salmonicida* (“the salmonid killer”) is a threat to sustainable aquaculture because it is able to cause systemic infections in farmed Atlantic salmon, Chinook salmon and Arctic char [16], [17]. Gross pathologies of *S. salmonicida* include internal haemorrhaging, splenomegaly and granulomatous lesions in the liver and spleen. In Northern Norway, outbreaks of spironucleosis in farmed Atlantic salmon, *Salmo salar*, is a recurring problem and causes mass mortality and economical loss. Drug treatment is not possible, making studies of the parasite important to develop alternative strategies [14]. The pathogenic *S. salmonicida* is genetically different from the morphologically indistinguishable diplomonad *Spironucleus barkhanus* which is a commensal in wild freshwater populations of Arctic char and grayling *Thymallus thymallus* [17], [18]. The parasite has recently been identified in both wild Arctic char and brown trout but no indications of disease were observed [19]. This suggests that wild salmonids might be asymptomatic reservoir hosts and that *S. salmonicida* is an opportunistic pathogen. We have indeed a very limited knowledge about how this important fish parasite is transmitted between hosts or the life cycle in general, how it is able to form the deadly lesions, the virulence genes responsible for the invasive infections and how it avoids the fish immune system during infection.

We have developed a stable transfection system for *S. salmonicida* to study the parasite [20]. Here we present a thoroughly annotated genome sequence and comparative analyses to *G. intestinalis*. We identify large differences in transcriptional regulation, the metabolic capacity and candidate variable surface proteins, which is in agreement with phenotypic differences between the species. In contrast, conservation of genes involved in encystation suggests similar machineries for that stage in the life cycle. The development of *S. salmonicida* into a model system contributes to the understanding of the pathogenicity and evolution of this enigmatic eukaryotic group, as well as eukaryotes in general.

## Results and Discussion

### Sequencing and assembly

The genome of *S. salmonicida* (ATCC 50377) was characterized using the optical mapping method provided by OpGen. The optical maps indicate a genome size of 12.6 Mbp distributed in nine chromosomes. The size is in good agreement with earlier estimates using flow cytometry [18], and similar to *G. intestinalis* (Table 1). We sequenced the genome *de novo* using a complimentary approach of 454 FLX to get large scaffolds and Illumina to increase sequence quality. The Illumina technology was also applied on RNA to get RNA-Seq data. This yielded a draft assembly of the genome containing 452 contigs in 233 scaffolds with a total length of 12.9 Mbp. The number of contigs and scaffolds are slightly more than the first *G. intestinalis* genome, but less than the two subsequently published genomes [10], [21], [22]. The largest scaffold is 0.56 Mbp in size, and the scaffold N50 is 0.15 Mbp. The average coverage of 454 and Illumina reads were 40× and 280× in the selected draft assembly, respectively.

**Table 1:** Comparison of the *S. salmonicida* and *G. intestinalis* genomes.

	*S. salmonicida*	*G. intestinalis*
Size (Mbp)	12.9	11.7
Chromosomes	9	5
G+C content (%)	33.4	49.0
Proteins annotated	8067	5901
Mean gene length (aa)	373	530
Gene density per kbp	0.63	0.50
Coding percentage (%)	72.1	78.2
Mean intergenic distance (bp)	421	481
Introns	3	6
tRNAs	145	63

The *S. salmonicida* genome is not very repetitive. Only 5.2% of the genome was masked by RepeatMasker (http://www.repeatmasker.org/), with 4.8% of genome as low complexity. The allelic sequence heterozygosity is ∼0.15%, which is much lower than the *G. intestinalis* GS genome, but higher than *G. intestinalis* WB [10], [21]. 64.8% of the draft genome can be mapped onto the optical maps. The scaffolds which could not be mapped have mostly sizes below 40 kbp, which is the limit for a sequence to be able to uniquely map to an optical map due to the expected frequencies of restriction sites. The relatively low number of scaffolds and the good agreement with the optical maps suggest that our selected draft assembly is of high quality and suitable to perform whole genome analyses of this diplomonad and compare the results to previously published genomes.

### Annotation and analysis of coding and intergenic regions

**Figure 1:**
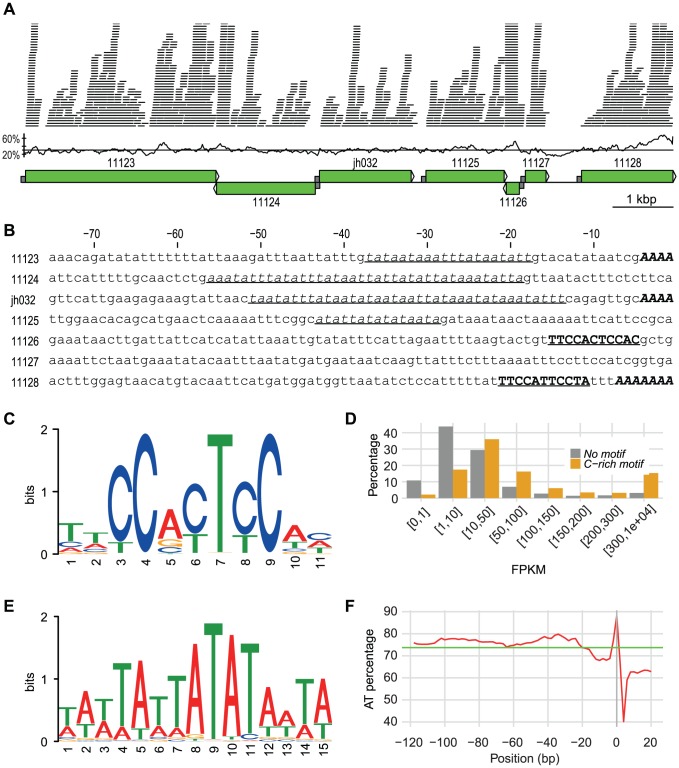
A 10 kbp genomic region with promoter info. A 10 kbp long genomic region located on scaffold scf7180000020498. The first part shows the Illumina RNA-Seq reads mapped onto the region. A coverage cutoff 40 was used for a better display. The GC content in 100 bp windows with a step size of 20 bp is shown. The average GC content in the region (30.5%) is indicated by a line. Green boxes with arrows indicate position and direction of annotated genes. Half sized grey boxes indicate 75 bp promoter regions with sequences shown in B. Numbers refer to protein IDs: 11123, Phospholipid-transporting ATPase; 11124, Hypothetical protein; jh032, Hypothetical protein; 11125, Long-flagella protein, kinase, CMGC RCK; 11126, Ribosomal protein S30; 11127, Prefoldin subunit 6; 11128, ATP-dependent RNA helicase. **B**. 75 bp promoter sequences of the genes shown in A. TATA-box motifs are in underlined italic font, C-rich motifs are in bold underlined upper cases, and the multiple As before start codon are in bold italic upper cases. **C**. Sequence logo of C-rich motif. **D**.Comparison of expression between genes with and without C-rich motif. Genes are divided into eight categories based on their FPKM-values. The y-axis represents the percentage of genes within each category. **E**. Sequence logo of TATA-box motif. **F**. AT contents in percentage of the 20 bp C-terminus of the genes with their 120 bp promoter regions drawn with window size of 3 and step size of 2. X-axis shows the positions, while y-axis shows the AT percentage. Green line indicates the average AT percentage of the regions. Plot was drawn in R.

We developed an annotation pipeline in-house during this project, which combines results from various sources (details described in methods and Protocol S1). Using the pipeline we annotated and manually inspected 8067 genes with an addition of 267 partial genes and 21 pseudogenes. Since the *S. salmonicida* genome is divergent from previously sequenced genomes, only 3164 of the genes have functional annotations including 879 genes annotated with domain information. The remaining 4903 genes code for hypothetical proteins, with 847 of those displaying similarity to genes in other species. The RNA-Seq data mapped well to open reading frames with clear boundaries (Figure 1A). This correlation was used as an indicator of functional genes during the manual review of the annotation.

The low frequency of introns (see below) makes gene identification easier in this genome than in many other eukaryotes. On the other hand, *S. salmonicida* uses an alternative genetic code in which only a single termination codon is used [23]. This leads to few termination codons and a high frequency of random open reading frames. In combination with the large genetic distance to the closest previously sequenced genome, this makes gene identification challenging. We believe that our annotation is of fairly high standard also for the hypothetical genes since 80% (3909 out of 4903) of them have support from RNA-Seq data and the average length of hypothetical genes is 345 bp close to 417 bp for the genes with functional annotation. Together this suggests that most genes annotated as hypothetical genes are functional genes.

Interestingly, *S. salmonicida* has around 3000 more annotated protein-coding genes than *G. intestinalis*, but with genes overall shorter at an average length of 373 aa, the percentage of the coding regions in the genome is still less, 72% versus 78% (Table 1). There are 47 cases of overlapping genes with an average overlap length of 38 bp. The genome has an average GC content of 33.4% and the coding regions have on average higher GC content (36.2%) compared to the intergenic regions (25.4%). 3268 *S. salmonicida* genes are found to be shared with 3089 *G. intestinalis* genes, whereas 4799 lack detectable homologs in the human parasite (Table S1). The average level of amino acid identity is 39.4% for orthologous genes (Figure S1). Similarity searches against available sequences in the public databases identified homologs for 182 of the *S. salmonicida* genes missing in *G. intestinalis*. The remaining 4617 genes lack detectable homologs, making 57.2% of the genes unique to *S. salmonicida* among the available sequenced genomes. We found little conserved synteny between *S. salmonicida* and *G. intestinalis* using the shared genes identified.

The largest clusters of *S. salmonicida* proteins consist of cysteine-rich proteins (discussed below) and proteins with protein kinase domains. Using a combination of three approaches we identified 138 putative protein kinases. These were classified according to Kinase.com database (http://kinase.com/) and the result was compared to the *G. intestinalis* kinome [24] (Table S2). One distinct difference between the two diplomonads was observed. While *Giardia* devotes 71% (198 out of 278) of its kinome to the NIMA (Never in Mitosis Gene A)-Related Kinase (NEK) family [24], *S. salmonicida* has only 18. NEK kinase family is known to regulate entry to mitosis [25] and flagella length [24]. It is universally present in eukaryotes, but typically found in fewer than 10 copies [24], thus is only slightly expanded in *S. salmonicida*. Thus, NEK kinases have expanded in the *G. intestinalis* lineage and the biological function of this massive expansion is likely not shared with *S. salmonicida*.

In contrast to NEK kinases, tRNAs are more abundant in *S. salmonicida*; there are 145 tRNA genes in the fish parasite genome, compared to 63 in the *G. intestinalis* genome. The tRNAs code for all 20 standard amino acids as well as one selenocysteine tRNA, and all expected tRNA synthetases were found during the annotation. Five 5S ribosomal RNA (rRNA) genes were identified in different locations of the genome, and one copy of 5.8S, 18S and 28S rRNAs were found in a single small contig which most likely is a collapse of repetitive reads since the contig has a ten times higher read coverage than the genomic average. Homologs to all ribosomal proteins found in *G. intestinalis* are present in the *S. salmonicida* genome. Three additional short ribosomal proteins, S30, L29e and L39, were identified which lack recognizable homologs in *G. intestinalis*. Another difference is that *S. salmonicida* encodes multiple copies of a dozen ribosomal proteins, whereas *G. intestinalis* only has a single gene for each subunit. Thus, *S. salmonicida* has more genes devoted to the core protein synthesis machinery than the previously studied diplomonad.

### Diplomonad genomes retain few and different introns

Although the splicing machinery is ancestrally present in *G. intestinalis* [26], only six introns have been identified in the three sequenced *G. intestinalis* genomes [27], [28]. Using our RNA-Seq data we could identify four putative introns in the *S. salmonicida* genome. Three of these were confirmed using RT-PCR. One is in a gene coding for ribosomal protein S24, one in the gene for ribosomal protein L30, and one in a gene for an unknown protein. The fourth intron in a hypothetical protein makes a short extension on the N-terminus, and the extension is only weakly expressed according to the RNA-Seq data. This could be an intron on its way to be lost, leading to a shorter protein.

*S. salmonicida* introns show the canonical GT/AG splice sites and are similar to the ones in *Giardia* and *Trichomonas vaginalis*. *S. salmonicida* introns contain a conserved AC-repeat motif, ACTAACAAACTAG, similar to ACTAACACACAG in *T. vaginalis* [29] and [AC]CT[GA]AC[AC]CACAG in *Giardia* [10] (Figure S2). This indicates that excavates likely have a shared intron splicing mechanism, strongly supporting an ancient presence probably followed by extensive intron loss in the two diplomonad lineages. Introns are found in different genes in *G. intestinalis* and *S. salmonicida*. Thus, the intron loss may eventually go to completion because there might not be a single diplomonad gene that requires the presence of an intron. Three split introns have been found in two genes in the *G. intestinalis* genome [27], [28], in addition to the canonical introns. Genes containing split introns are encoded from different loci in the genome and the transcripts from these are trans-spliced into a single mRNA used in translation. The homologs to the genes containing split introns in *G. intestinalis* [28] were found intact without introns in *S. salmonicida*. Further attempts failed to reveal split introns in *S. salmonicida* from the currently available data. The splicing machinery in *G. intestinalis* is highly reduced [10]. When we analyzed the machinery in *S. salmonicida* very similar results were obtained; nine Sm-like proteins were identified, as was putative Prp8, 22, 28 and 43 proteins.

### Divergent signal peptides in *S. salmonicida*

Signal peptides are present in the N-terminal of newly synthesized proteins destined to the secretory pathway. We used SignalP, version 4.1 [30], to identify putative signal peptides in the diplomonad genomes. The method predicted 381 and 109 proteins in the *G. intestinalis* and *S. salmonicida* genomes, respectively. Looking into five orthologous groups which had members with predicted signal peptides in both organisms, we realized that certain *S. salmonicida* orthologs carry weaker signals. For example, a group of sugar transporters contains multiple *S. salmonicida* proteins with predicted signal peptides as well as members that share the characteristic pattern in the SignalP analysis, but score below the threshold (Figure S3). A similar case was found in the cyst wall proteins (see below). This indicates that *S. salmonicida* has more signal peptides than predicted, with some signal peptides being divergent and therefore are not recognized by the currently available methods. An updated profile could be used in future improved searches for signal peptides in this genome, given that experimental studies confirm the function for these signal peptides scoring below the threshold using the available profile.

The signal recognition particle (SRP) binds the signal peptide when it emerges from the exit site of the translating ribosome [31]. The complex of the translating ribosome and the SRP particle docks to the signal recognition receptor in the ER membrane and the nascent protein is translocated through the Sec61 channel. Most eukaryotic SRPs contain six proteins and the 7S RNA, divided into the Alu and S domains [31]. The two proteins in the Alu domain are absent from the *S. salmonicida* genome, and the Alu-domain is missing in the 7S RNA (S. Svärd, unpublished results). The role of the Alu domain is to arrest translation elongation just after the signal sequence emerges from the ribosome [31] to provide a time window for translocation of the nascent chain into the ER. The lack of an Alu domain in the *S. salmonicida* and *G. intestinalis* [22] SRP suggests that this process is regulated differently in diplomonads. The SRP 72 protein of the S domain could neither be identified; nor could the Sec61-β and Sec61-γ subunits. Thus, SRP and its interacting proteins are highly diverged in *S. salmonicida*, in line with the diverged signal peptides.

### Identification of an abundant C-rich motif that is a putative promoter in *S. salmonicida*

Transcription has been found to be loosely regulated in *G. intestinalis* with fuzzy boundaries of gene transcripts and a relative high fraction of anti-sense transcription [27], [32]. This is coupled to an absence of conserved promoter motifs in *G. intestinalis* except AT-rich sequences at the transcription start sites [12]. The picture is different in *S. salmonicida* which show more specific boundaries of transcripts for most genes (Figure 1A). Putative regulatory elements included a TATA-box motif, a C-rich motif and an enrichment of As which probably served as transcription initiator element (Figure 1B).

We identified a conserved 11-nt C-rich motif in the upstream regions of annotated genes (Figure 1C) shared by 16.7% of the *S. salmonicida* genes. This motif is preferentially found in a position around 10 bp upstream of the initiation codon and is a strong candidate for being part of a promoter. Indeed, genes with a C-rich motif in the promoter region are more often observed to be highly expressed as measured by RNA-Seq reads (Figure 1D). Conserved house-keeping genes are over-represented among the genes that have this C-rich motif, for example 71 out of 82 ribosomal proteins are connected with the motif. These observations suggest that the C-rich motif is a strong candidate for a promoter sequence in *S. salmonicida* which should be tested further experimentally.

Similar C-rich motifs are found in the two other diplomonads with sequence data available, *S. vortens* and *G. intestinalis*, as well as the parabasalid *T. vaginalis*, but with much lower frequencies. For example, there are only around 1% of *G. intestinalis* genes that have similar C-rich motifs, and majority of those are hypothetical genes. For *S. vortens* and *T. vaginalis*, we found C-rich motifs in 5% and 1% of the analysed genes, respectively (Figure S4A). Thus, the motif is most frequent in the *Spironucleus* genomes, suggesting similarities in gene regulation. Alternative motifs were found upstream of smaller subsets of the annotated genes. For example, cyst wall proteins shared a putative promoter motif (see below).

A divergent TATA-binding protein (TBP) is found in *G. intestinalis*, although no conserved TATA-motifs are present [33]. The putative TBP is even more divergent in the *S. salmonicida* genome. It was identified as hypothetical protein that contained a divergent TBP domain, the amino acid sequence did not show significant similarities to the *Giardia* or any other TBPs in standard searches. Nevertheless, this putative protein has many potential binding sites within the genome because a clear TATA-box motif was detected in the promoter regions of 80.7% of the genes in *S. salmonicida* (Figure 1E). This adds to the picture of the presence of a more elaborate transcriptional regulation in this organism.

A third distinct sequence pattern was found using the sequence logo method on the 5′ end of the genes. There is an enrichment of As immediately upstream of the start codon in the *S. salmonicida* genes (Figure S4B). In fact, in 33.0% of the *S. salmonicida* genes, we observed at least three As right before ATG start codon. This AAA signal is probably part of the transcription initiator element (Inr). We see no clear difference on transcription expression levels between genes with and without the AAA using RNA-Seq data.

We analysed the AT content upstream of all protein-coding genes. The location of TATA-box motif corresponds well to the AT percentage peak between −60 bp to −20 bp, the C-rich motif corresponds to a dip around −10 bp, and the putative Inr-element are shown as an increase of the AT-content close to the start codon (Figure 1F). *S. vortens* has similar AT percentage dip as well as similar C-rich motif, whereas *G. intestinalis* without the general C-rich motif does not have the AT percentage dip upstream of the start codon (Figure S3CD).

The distinct motifs upstream of genes can only function as regulatory elements if they are recognized by DNA-binding proteins. Therefore we searched the proteome for homologs of such protein families previously analysed [34]. We found that there was a large expansion of proteins containing Myb-like DNA binding domains; there are 107 such proteins annotated in the *S. salmonicida* genome, compared to 8 in the *G. intestinalis* genome. Myb domain-containing proteins are also expanded in *T. vaginalis* [34]. Furthermore, *S. salmonicida* has several other putative transcription factors similar to C2-H2 Zn-finger, E2F and DP1 transcription factors. Taken together with the observation of several putative promoter motifs (Figure 1), it is very likely that there are differences in the regulation on the transcriptional level in *S. salmonicida* compared to in *G. intestinalis*.

Tight regulation of genes is important for organisms living in fluctuating environments, and probably a feature of free-living ancestors of these parasites. The extent of regulation on the post-transcriptional level is not well-known from any diplomonad, but here we report observations that suggest differences of the potential for transcriptional level regulation which may be coupled to different life styles. The retention of extensive transcriptional regulation in *S. salmonicida*, but not in *G. intestinalis*, could be the basis for its ability for systematic infections in the fish during which it is likely to permit radically different microenvironments. *G. intestinalis*, on the other hand, can only grow within a defined part of the intestine of its host, a comparatively stable environment with less need of transcriptional regulation.

### Overlap of the polyadenylation signal and termination codon may lead to codon reassignment

In sequence surveys of *S. salmonicida* and *S. barkhanus*, a conserved motif was found around the termination codon connected with the presence of a polyA tail around 14 bp downstream, suggesting that the termination codon and the polyadenylation signal overlap [8], [18]. Here we show that this signal is not restricted to highly expressed genes. The sequence logo analysis of 3′ end of the genes reveals a dominance of A and T two positions upstream and G right before the only stop codon TGA used in *S. salmonicida* (Figure S5A). This putative signal, AGTGA, shows a similar pattern to the most used polyadenylation signal, AGTAAA, in *Giardia* [12], [27]. A 4-bp polyadenylation signal, TAAA, was recently functionally identified in *T. vaginalis* [35]. Strikingly, TAA is used in ∼90% of the genes in that genome and in more than half of the analysed genes the position of the polyadenylation and termination overlapped.

The fact that the termination codon serves as the core motif in the polyadenylation signal could indeed explain how a change of genetic code could happen. If only a single termination codon can serve as a polyadenylation signal, there could be a preferential use of that codon at the 3′ end of all genes in a genome, provided there is selection for an overlap of termination codon and polyadenylation signal. As a consequence, the other two codons will be free to adapt new functions. This could indeed be an explanation for the code re-assignment in a subset of the diplomonads [23].

The polyadenylation machinery in *Giardia* is highly reduced compared to the corresponding machinery in yeasts [10]. An analysis of the polyadenylation machinery in *S. salmonicida* shows the same picture (Figure S5B) with only a few of the proteins identified. Interestingly, *Giardia* and *Spironucleus* seem to be missing the same proteins, which have been lost or degenerated to such a degree that they cannot be identified using sequence-based searches.

### Putative encystation pathway

Transmission routes of piscine *Spironucleus* species have not been mapped and there are only anecdotal reports of cysts. *Spironucleus vortens* has recently been shown to survive for more than 30 days outside the host in feces [36] but this has not been observed in *S. salmonicida*. Cysts have been detected in the terrestrial species *Spironucleus muris* and *Spironucleus meleagridis* and their cysts show immunological cross-reactivity to the cyst wall of *Giardia* cysts [37], [38]. Here we use comparative genomics tools in combination with functional characterizations to study the putative presence of a cyst stage in *S. salmonicida*.

**Figure 2:**
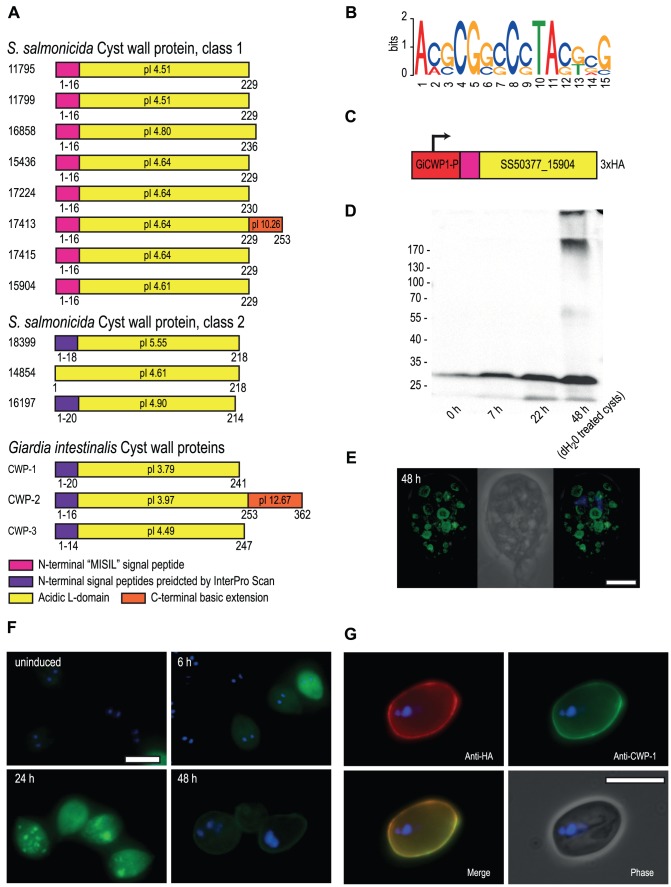
*S. salmonicida* cyst wall proteins traffic in *G. intestinalis* ESVs and incorporate into the cyst wall. **A**. Schematic representation of candidate *S. salmonicida* cyst wall proteins (CWPs). Numbers refer to protein IDs and conserved features are shown by coloured boxes with the amino acid positions indicated. The isoelectric point (pI) is indicated. **B**. Sequence logo of motifs upstream of the eight class 1 CWPs, glucosamine-6 phosphate deaminase, two glucose 6-phosphate N-acetyltransferase and two UDP-glucose 4-epimerases. **C**. The construct used to express the candidate *S. salmonicida* class 1 CWP in *G. intestinalis* during encystation. The red box indicates the promoter region of *G. intestinalis* CWP-1. **D**. Western blot of samples taken from *G. intestinalis* transfectants carrying the *S. salmonicida* CWP construct. Expected size of the epitope-tagged SS50377_15904 is 28.4 kDa. **E** and **F**. Immunofluorescence analysis of *G. intestinalis* transfectants at different time points into encystation. The protein was detected using anti-HA conjugated to AlexaFluor488 (green) and the nuclei were labelled using DAPI (blue). Scale bars, 5 µm. **G**. Immunofluorescence micrograph of a *G. intestinalis* transfectant at 48 h post inducation of encystation following water-treatment. The cysts were stained by rabbit anti-HA and detected by anti-rabbit Alexa Fluor 594 (red), and probed using anti-CWP-1 conjugated to FITC (green). Scale bar, 10 µm.

The production of the environmentally resistant cyst wall that protects the cyst from the harsh environment outside the host has been extensively studied in *Giardia*. The cyst wall is composed of the aminosugar N-acetylgalactosamine (GalNAc) as well as three cyst wall proteins (CWP 1–3) [13]. The five enzymes needed to produce GalNAc from fructose 6-phosphate are all present in *S. salmonicida*. The analysis also uncovered a family of eight highly similar candidate cyst wall proteins with homology to *Giardia* CWP-1 (Figure 2A). We also identified another three potential cyst wall proteins that displayed a higher degree of divergence. The former family of genes included a potential homolog of *G. intestinalis* CWP-2 which also carries a short basic extension at the C-terminal similar to that protein, although the extension is substantially shorter in the *Spironucleus* protein (Figure 2A). The presence of regulatory motif upstream of encystation-inducible genes has been noted in *G. intestinalis* [39]. We have also found a conserved motif in the promoter regions of the conserved encystation related proteins in *S. salmonicida* (Figure 2B). The comparative genomic analyses strongly suggested a cyst stage in the *S. salmonicida* life cycle. This was further tested experimentally.

### *S. salmonicida* cyst wall proteins traffic in *Giardia* ESVs and incorporate into the cyst wall

Attempts of inducing encystation in *S. salmonicida* using cues traditionally employed in *G. intestinalis* (increased bile concentration and pH or cholesterol-deprivation) have not been successful at triggering cyst formation in *S. salmonicida*. Neither are cyst-like objects observed during routine *in vitro* passaging of the parasite. However, *S. salmonicida* cyst wall proteins display similar characteristics as *G. intestinalis* cyst wall proteins in analyses for signal peptides, even though they score below the threshold (Figure S6). This suggests that they may be functional in *G. intestinalis*. To test this we fused a *S. salmonicida* cyst wall protein to the *G. intestinalis* CWP-1 promoter, inserted a C-terminal 3×HA epitope tag and established *G. intestinalis* transfectants carrying an episomal plasmid with the construct (Figure 2C). This particular *S. salmonicida* cyst wall protein does not contain any TAG or TAA codons that in *S. salmonicida* encode glutamine. We proceeded to induce encystation in *Giardia* and studied the expression of the *S. salmonicida* cyst wall protein by Western blot and immunofluorescence (Figure 2DEFG). *G. intestinalis* transfectants carrying epitope-tagged SS50377_15904 under transcriptional control of the CWP-1 promoter were encysted and samples were taken at 0, 7, 22 and 48 h for analysis by Western blot. The sample at 48 h was water-treated to yield water-resistant cysts. The SS50377_15904 construct is induced upon encystation and is found to be present in high molecular weight protein species resistant to non-reducing conditions at 48 h post induction (Figure 2D). We further studied the construct by immunofluorescence at different time points into encystation (Figure 2EF). At 6 h post induction the cells show expression of the protein in the cytosol. At 24 h some cells show partial sorting of the protein into maturing encystation-specific vesicles (ESVs). In some cells the protein was present in the doughnut-shaped fluid-phase of the ESV, in a similar way as *Giardia* CWP-1 [40] (Figure 2EF). This pattern is more pronounced in some cells at 48 h (Figure 2E). At 48 h post induction the protein can also be detected as incorporated in the cyst wall in a subpopulation of the cells (Figure 2F), as judged by the co-localization with *G. intestinalis* CWP-1 (Figure 2G). These results show that *S. salmonicida* has a cyst wall protein that is functional in *G. intestinalis*, and supports the hypothesis that the parasite has a cyst stage in the life cycle.

### Proteases

*S. salmonicida* has to be able to degrade the host tissue to invade different organs in the fish. We identified that the *S. salmonicida* degradome consists of 111 protease homologs. The *S. salmonicida* proteases are divided into four catalytic classes and 26 families according to Merops protease classification [41] (Table S3). It includes 50 cysteine proteases belonging to 7 families; 34 metallo proteases belonging to 13 families, 8 serine proteases belonging to 4 families, and 14 threonine proteases belonging to the T1 family. Cysteine proteases from parasites are important virulence factors and known to degrade the host's extracellular matrix during invasion [42]. *S. salmonicida* has slightly more proteases in this category than *Giardia*, which is in agreement with its invasive phenotype [16].

### *S. salmonicida* contains novel classes of cysteine-rich membrane proteins

Pathogens need to constantly avoid the host immune system to be able to strive within the host. A common way to do this is by antigenic variation by frequent changes of the cell surface which is the part exposed to the host immune system. In *G. intestinalis* this is done by a protein family of variant-specific surface proteins (VSPs) [43]. Hundreds of genes encoding VSPs are found in the *G. intestinalis* genomes and these are among the most divergent protein families within the *Giardia* genomes, both within and between isolates [22], [43]. Only one VSP is expressed at a time, and expression is switched from one VSP to another every 6 to 13 generations [44]. In this way a new VSP will be exposed on the surface of the parasite before it has been recognized by the adaptive immune system. *Giardia* VSPs are cysteine-rich (∼12% cysteine) with frequent CXXC motifs and a conserved C-terminal membrane domain which is followed by a hydrophilic cytoplasmic tail with a conserved five amino acid CRGKA signature sequence [43].

**Figure 3:**
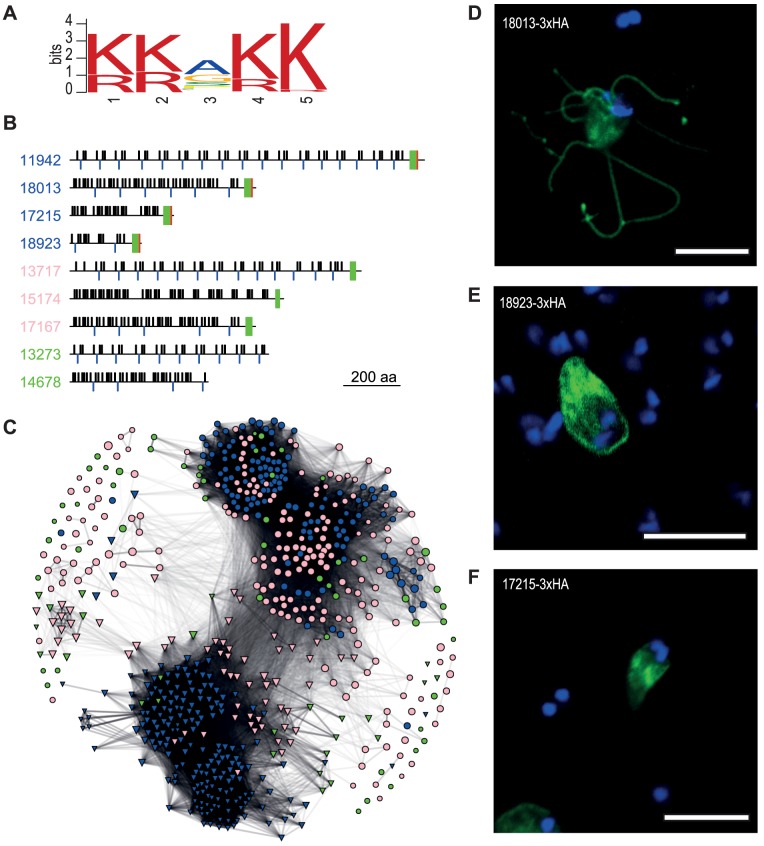
Diverse families of *S. salmonicida* cysteine-rich proteins. **A**. Sequence logo of a five amino acid motif shared by CRMP-1. **B**. Schematic view of selected *S. salmonicida* cysteine-rich proteins. Numbers refer to protein IDs and blue font indicates CRMP-1, pink CRMP-2 and green CRP. Black bars above and blue bars below the line indicate CXXC and CXC domains, respectively. Green and red boxes indicate TM domain and the conserved five amino acid motif shown in A, respectively, the basis for the classification. **C**. Network analysis of cysteine-rich proteins. *S. salmonicida* genes are represented in circle whereas *G. intestinalis* genes are shown in triangle. Blue indicates VSP or CRMP-1, pink indicates HCMP or CRMP-2, and green indicates HCP or CRP. Edges are weighted and scaled by reciprocal BLAST scores. Node sizes are scaled by protein sizes. **DEF**.Members of the *S. salmonicida* CRMP-1 protein family localise to different membrane domains. Stably transfected *S. salmonicida* carrying the pSpiro-PAC-18013-3×HA (D), pSpiro-PAC-18923-3×HA (E) and pSpiro-PAC-17215-3×HA (F), episomal plasmids.

*S. salmonicida* also harbours many cysteine-rich proteins. In a subset of these we identified a [KR][KR]X[KR][KR] motif (Figure 3A, Figure S7) towards the C-terminal which is reminiscent of the *Giardia* CRGKA signature sequence in VSPs. We classified the *S. salmonicida* cysteine-rich proteins into three groups based on the presence and absence of CXXC, CXC, KKXKK motifs and a C-terminal transmembrane (TM) domain (Figure 3B, Table S4). We named the groups cysteine-rich membrane protein 1 (CRMP-1), cysteine-rich membrane protein 2 (CRMP-2) and cysteine-rich protein (CRP).

The CRMP-1 group contains 125 membrane proteins with a conserved C-terminal five amino acid KKXKK motif, and three or more CXXC and CXC motifs. This group of proteins are similar to *Giardia* VSPs, but with a slightly different C-terminal sequence (Figure 3B). Some of the proteins contain an extra tail after this motif (Figure S7). The similarity to *Giardia* VSPs suggests that they may function as variable surface proteins in *S. salmonicida*. However, di-lysine motifs in the C-terminal of type I integral membrane proteins have been found to be both necessary and sufficient for endoplasmic reticulum (ER)-retention in other eukaryotes [45]. Protein disulfide isomerase-2 is a type-I integral membrane protein that carries a consensus C-terminal di-lysine motif for (KKAKKSE) and localises to the ER in *S. salmonicida* [20]. It is possible that the C-terminal tail of CRMP-1 proteins function as an ER retention signal.

The CRMP-2 group contains 195 cysteine-rich proteins which lack the conserved five amino acids signature sequence, but contain three of more CXXC or CXC motifs as well as a C-terminal TM domain (Figure 3B). The combination of CXXC and CXC motifs and a transmembrane domain, but lack of the five amino acid signature sequence found in CRMP-1, make the CRMP-2 more similar to the class of high cysteine membrane proteins (HCMP) in *G. intestinalis*. A study in *Giardia* showed that HCMPs were structurally similar to that of VSPs, but the only characterized HCMP was regulated and expressed similar to a cyst wall protein (CWP) [46].

We have also annotated 52 CRPs which contain more than 10% cysteines but do not belong to the other two categories (Figure 3B).

We performed a network analysis based on bi-directional BLAST hits to determine the relationship between and within the groups of *S. salmonicida* and *G. intestinalis* cysteine-rich proteins (Figure 3C). Strikingly, the cysteine-rich proteins from the two parasites are found in two distinct clusters with very little overlap. Some of the proteins show very little sequence similarity within the protein family and are found outside these clusters. Two different evolutionary scenarios could create such a pattern. It could be that the families of cysteine-rich proteins have expanded independently in the two parasites, or there is a high gene-turnover rate in which new cysteine-rich proteins are created via duplication events with a similar rate as genes are lost.

The patterns within the species-specific clusters are different (Figure 3C). The majority of the *G. intestinalis* VSPs are found in two clusters, with the HCMPs and high cysteine proteins (HCPs) loosely associated with these. The clustering within the *S. salmonicida* cluster is less distinct. CRMP-1 and CRMP-2 tend to cluster within the same class, but there are also intermixing between groups. Thus, the network analysis suggests that the classification into VSPs and HCMPs according to the presence of the C-terminal motif has a stronger correlation with overall primary sequence similarity in the proteins in *G. intestinalis* than in *S. salmonicida*.

The presence of transmembrane domains of the CRMP-1 and CRMP-2 proteins suggests that they are localised to either internal or external membranes in the cell. To test this, we epitope tagged three selected CRMP-1 proteins and studied their cellular localization (Figure 3DEF). This analysis shows one that localises like VSPs to the surface of the cellular body and flagella (Figure 3D), one that localises to the cellular body (Figure 3E) and one that localises to ER-like structures (Figure 3F). It remains to be determined whether different CRMPs are exposed at different time points of infection, as expected if they are responsible for antigenic variation in the fish parasite. Likewise, additional data are needed to determine if these proteins are under positive selection for variation because the available sequences are too divergent for such analyses.

It has been determined that the expression of VSPs in *Giardia* is regulated by components of the RNA interference pathway [47]. However, we failed to find Dicer, Argonaute and RNA-dependent RNA polymerase, key component of the RNA interference pathway, in the *S. salmonicida* genome. This indicates that CRMP expression in *S. salmonicida* is regulated by some other unknown means. We neither found any secretion signal peptides in the N-terminal of the protein as in *Giardia*. Either the signal peptides used by *S. salmonicida* are very divergent from what is known, or the secretion of CRMP proteins to the surface of the cell is regulated by some unknown means. 74% of the *Giardia* VSPs share Inr of PyAatgTT [43], while in *S. salmonicida*, 85 out of 125 CRMP-1s have the common AAAatg Inr without any clear conserved bases after start codon. Obviously, the *S. salmonicida* CRMPs are regulated differently from *G. intestinalis* VSPs, although the CRMPs show structural similarities and localise to the surface of the cell as VSPs. It may be that the two parasites independently have developed different mechanisms for expression of large families of cysteine-rich membrane proteins as an adaptation to a parasitic life style.

### *S. salmonicida* has a more extensive metabolism and more transporters than *G. intestinalis*

Diplomonads were previously viewed as primitive eukaryotes harbouring bacterial-like metabolism [11]. The prokaryotic features of the enzymes later turned out to be explained by recent gene acquisitions rather than ancient retention of the genes by genomic studies of *G. intestinalis* and *S. salmonicida* [8]–[10], [48]. This metabolic adaptation via lateral gene transfer has, for example, contributed to the anaerobic metabolism of these organisms. With the complete *S. salmonicida* genome we can have more insights into the similarities and differences in the metabolism of these two pathogenic diplomonads.

*S. salmonicida* and *G. intestinalis* genes were classified into functional categories (Table 2). *G. intestinalis* has more enzyme functions represented in only two categories: slightly more enzymes involved in glycan metabolism were detected, and *S. salmonicida* lacks the mevalonate pathway (in Metabolism of terpenoids and polyketides) which is present in *Giardia*. In the other nine categories *S. salmonicida* has more enzymes. The largest differences in the metabolism are observed for the categories carbohydrate metabolism, energy metabolism and amino acid metabolism in which *S. salmonicida* has 32, 21 and 26 more enzymes than *G. intestinalis*, respectively.

**Table 2:** Metabolic enzymes identified in the KAAS analysis.

	*S. salmonicida*	*G. intestinalis*
Carbohydrate metabolism	66	34
Energy metabolism	49	28
Lipid metabolism	26	16
Nucleotide metabolism	55	48
Amino acid metabolism	41	15
Metabolism of other amino acids	15	6
Glycan biosynthesis and metabolism	10	11
Metabolism of cofactors and vitamins	29	17
Metabolism of terpenoids and polyketides	5	13
Biosynthesis of other secondary metabolites	7	2
Xenobiotics biodegradation and metabolism	12	7

The higher number of metabolic genes in *S. salmonicida* suggests that this parasite can utilise more metabolites than *G. intestinalis*. We classified the putative transporter proteins into families to test if it also has a higher capacity for transport of metabolites. In total 219 putative transporters were identified in *S. salmonicida* compared to 138 in *G. intestinalis* (Table S5). The three most common families are the Major facilitator superfamily (MFS), the ATP-binding cassette (ABC) superfamily, and the Amino acid/auxin permease (AAAP) family. The MFS and SBC superfamilies have broad specificities including metabolites such as sugars and amino acids [49], [50]. Together these two superfamilies have 67 and 43 members in the *S. salmonicida* and *G. intestinalis* genomes, respectively (Table S5). The AAAP family, in contrast, transport only single or multiple amino acids [51]. Twenty AAAPs are found in the *S. salmonicida* genome, compared to nine in the *G. intestinalis* genome. These observations suggest that the fish parasite is able to transport a larger variety of metabolites than *G. intestinalis*, especially amino acids and sugars.

**Figure 4:**
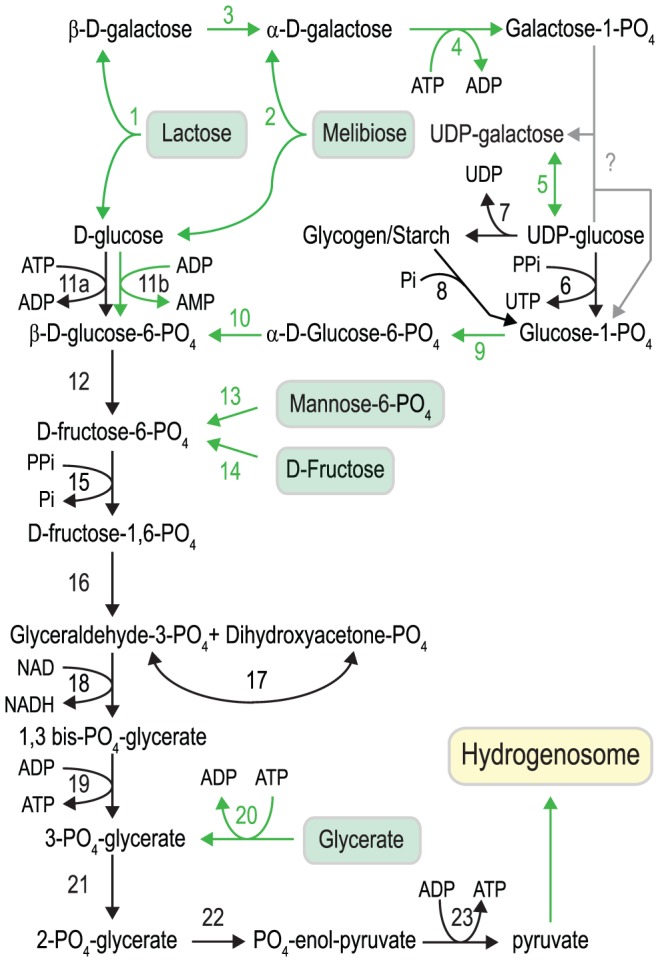
Carbohydrate metabolism in *S. salmonicida*. Black arrows indicate enzymatic functions present in both *G. intestinalis* and *S. salmonicida*, and green arrows indicate functions not detected in *G. intestinalis*. Key to enzymes: 1. β-galactosidase, 2. α-galactosidase, 3. galactose mutarotase, 4. galactokinase, 5. UDP-glucose 4′ epimerase, 6. UTP-glucose-1 phosphate uridylyltransferase, 7. glycogen synthase, 8. glycogen phosphorylase, 9. phosphoglucomutase, 10. glucose-6-phosphate 1-epimerase, 11a. glucokinase, 11b. ADP-specific glucokinase, 12. glucose phosphate isomerase, 13. phosphomannose isomerase, 14. Fructokinase, 15. pyrophosphate-dependent phosphofructokinase, 16. fructose bisphosphate aldolase, 17. triosephosphate isomerase, 18. glyceraldehyde 3-phosphate dehydrogenase, 19. phosphoglycerate kinase, 20. glycerate kinase, 21. phosphoglyceromutase, 22. enolase, 23. pyruvate kinase.

**Figure 5:**
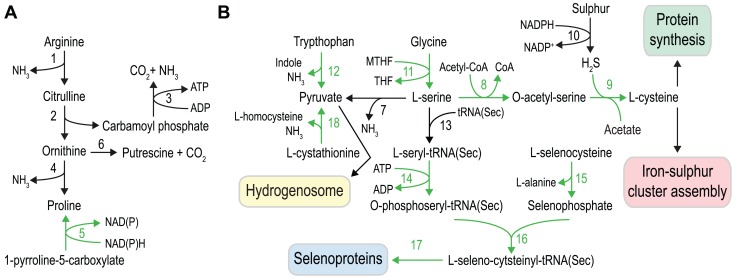
Amino acid metabolism in *S. salmonicida*. **A**. Arginine dihydrolase pathway and proline metabolism, and **B**. serine, cysteine, sulfur and selenium metabolism. Black arrows indicate enzymatic functions present in both *G. intestinalis* and *S. salmonicida*, and green arrows indicate functions absent in *G. intestinalis*. Key to enzymes: 1. arginine deiminase, 2. ornithine carbamoyl transferase, 3. carbamate kinase, 4. ornithine cyclodeaminase, 5. pyrroline-5-carboxylate reductase, 6. ornithine decarboxylase, 7. L-serine dehydratase, 8. serine O-acetyltransferase, 9. cysteine synthase, 10. sulfide dehydrogenase, 11. serine hydroxymethyltransferase. 12. tryptophanase, 13. seryl-tRNA synthetase, 14. *O*-phosphoseryl tRNA(Sec) kinase, 15. selenophosphate synthetase - NifS fusion protein, 16. *O*-phosphoseryl-tRNA(Sec) selenium transferase, 17. selenocysteine (Sec)-specific elongation factor, 18. cystathionine β-lyase.

We examined the metabolic capacity in more detail for some of the functional categories to understand the metabolic differences between the two diplomonads on a finer scale (Figures 4 and 5, Figure S8).

### Carbohydrates as energy sources

A major metabolic difference between the two diplomonads is that *S. salmonicida* harbours hydrogenosomes [4]. 20 proteins have been experimentally confirmed to be localised to the organelle, serving functions such as iron-sulfur cluster biogenesis, protein translocation, hydrogenase maturation and metabolic enzymes. We suggested a potential pathway for ATP production from pyruvate via the concerted action of hydrogenosomal pyruvate∶ferrodoxin oxidoreductase (PFOR), [FeFe] hydrogenases, ferredoxins and a potential acetyl-CoA synthetase (ADP-forming) [4]. The hydrogenosomal presence of serine hydroxymethyltransferase and a putative H-protein of the glycine cleavage system argue for that at least parts of the amino acid metabolism is localised to the hydrogenosome. Thus, the hydrogenosomes in *S. salmonicida* likely produce ATP from pyruvate, the end product of glycolysis. This provides *S. salmonicida* with an additional way of converting pyruvate into energy, compared to the hydrogenosome-lacking *G. intestinalis* (Figure S8).

*S. salmonicida* encodes an almost identical set of enzymes as *G. intestinalis* to perform glycolysis (Figure 4). However, the fish parasite has a more extensive capability to use different metabolites to feed into the pathway. Mannose-6-phosphate and fructose can be converted into fructose 6-phosphate by phosphomannose isomerase and fructokinase, and *S. salmonicida* appears to be able to utilise glycerate by the action of glycerate kinase to generate 2-PO_4_-glycerate, the substrate for enolase in the penultimate step of glycolysis (Figure 4). We also identified homologs to α-galactosidase and β-galactosidase, two enzymes which catalyse the hydrolysis of galactosides into monosaccharides. The enzymes have glycolipids and glycoproteins as substrates, as well as the disaccharides lactose and melibiose (Figure 4). The glucose generated when these enzymes act on disaccharides shuttles directly into glycolysis whereas the galactose needs to be metabolised by a specialised set of enzymes (Figure 4). The Leloir pathway converts galactose to glucose-1-phosphate. We detected three out of the four enzymes (galactose mutarotase, galactokinase and UDP-glucose 4-epimerase) of the pathway in *S. salmonicida*. The third enzyme of the pathway, galactose 1-phosphate uridylyltransferase, could not be identified. This function is probably performed by an unidentified protein. Glucose-1-phosphate is then shuttled into the glycolysis via two additional enzymes detected in *S. salmonicida*, but absent in *G. intestinalis*: a phosphoglucomutase and glucose-6-phosphate 1-epimerase. Our bioinformatic analysis indicates that *S. salmonicida* likely can use five additional carbohydrates compared to *G. intestinalis* to feed into the glycolysis (Figure 4). However, experimental studies of the fish parasite are needed to test this hypothesis.

There are a large amount of *S. salmonicida* proteins putatively involved in end product synthesis in *S. salmonicida* (Figure S8). The pyruvate generated in the glycolysis can be converted to acetyl-CoA by the action of the five PFORs identified in the genome, shuttling electrons to [FeFe] hydrogenase via ferredoxin (Figure S8). The two [2Fe-2S] type ferredoxins in *S. salmonicida* have been localised to the hydrogenosome. *S. salmonicida* encodes seven iron-only hydrogenases, at least two of which are located in the hydrogenosome. In addition to these enzymes we identified eight flavodoxins in the genome. These are bacterial flavoproteins containing one molecule FMN that typically can replace the role of ferredoxin in electron-transfer functions. The presence of multiple paralogs of the proteins in these pathways and their different localizations [4] suggest that hydrogen and energy generation from pyruvate probably is occurring both in the hydrogenosome and the cytosol.

### Amino acids as energy sources

The capacity to metabolise amino acids appears to be greater in *S. salmonicida* than in *G. intestinalis*, and several of the differences suggest that the fish parasite utilise a variety of amino acids as energy sources (Figure 5). We identify all the enzymes of the arginine dihydrolase pathway, a rare pathway in eukaryotes that is present in both *G. intestinalis* and *T. vaginalis* [52], [53] and allows the utilization of arginine as an energy source (Figure 5A). Proline is potentially synthesized not only from ornithine cyclodeaminase but also from pyrroline 5-carboxylate using pyrroline 5-carboxylate reductase (ProC). The activity of ProC is dependent on cofactor F_420_ and we detect the presence of a putative F_420_:gamma-glutamyl ligase in the *S. salmonicida* genome that might be involved in the synthesis of the final cofactor. Tryptophan could also serve as an energy source in *S. salmonicida* due to the presence of three copies of a bacterial-like tryptophanase that generate pyruvate from tryptophan with the concomitant production of indole and NH_3_ (Figure 5). We previously identified a potential homolog of the H-protein of the glycine cleavage system in the hydrogenosomes of *S. salmonicida* as well as serine hydroxymethyltransferase. Consequently, *S. salmonicida* might employ a glycine cleavage system in the metabolism of glycine and serine. Serine can be used to generate pyruvate by L-serine dehydratase. Serine is also used in the synthesis of selenocysteine and in the *de novo* synthesis of cysteine via serine *O*-acetyl transferase and cysteine synthase A (Figure 5). Cystathionine might be used to generate pyruvate by the formation of homocysteine employing a cystathionine β-lyase. Energy generation is however not the only purpose of the *S. salmonicida* amino acid metabolism. In addition, the fish parasite has an extended set of enzymes for incorporation of cysteine and selenocysteine into proteins.

### Selenium metabolism

Selenium metabolism is a trait absent in *G. intestinalis* and *T. vaginalis*, but present in *Spironucleus* [18]. The tRNA(Sec) and the four enzymes needed to incorporate selenocysteine into protein with selenophosphate and L-serine as precursors were found in the *S. salmonicida* genome (Figure 5). The identified polypeptide coding for selenophosphate synthetase is fused with a NifS-like protein in its C-terminal end. It has been shown that NifS-like proteins can function as a selenocysteine lyase in *Escherichia coli* and *Arabidopsis* which delivers selenium to selenophosphate synthetase [54], [55]. The fusion suggests that the NifS-like part has a selenocysteine lyase activity, which would enable *S. salmonicida* to utilise selenocysteine for selenophosphate biosynthesis (Figure 5). This fusion has only been found in the bacterium *Caldithrix abyssi* (GI: 493985699); it probably replaced the cognate selenophosphate synthetase and provided *S. salmonicida* the ability to use ingested selenoproteins as a selenium source. Only three putative selenoproteins could be found, all selenoprotein W paralogs. However, selenium may be incorporated into proteins independent of selenocysteine in *S. salmonicida*. Selenophosphate synthetase has sometimes been found in prokaryotic genomes that lack other selenium utilization genes [56], [57]. A gene uniquely shared between such genomes was detected and speculated to be involved in incorporation of selenium into protein independent of selenocysteine [56], [57]. *S. salmonicida* has three identical homologs of this protein which previously has not been detected in any eukaryotic genome. Thus, it may be that selenium is used in other proteins than the identified canonical selenoproteins in the *S. salmonicida* genome.

### Sulfur metabolism in *S. salmonicida*

The large amount of cysteine-rich and iron-sulfur cluster containing proteins in *S. salmonicida* provides high demand of available cysteine. Sulfur is used in many metabolites and is essential for growth of all organisms, and inorganic sulfur is assimilated by photosynthetic organisms and fungi [58]–[60]. Other organisms often rely on uptake of reduced sulfur compounds from the environment [61]. *G. intestinalis* indeed seems to lack genes for biosynthesis of cysteine and methionine, sulfur-containing amino acids [10], [12]. On the other hand, protein-coding genes with sequence similarity to prokaryotic sulfide dehydrogenase [62], [63] have been found in *G. intestinalis* and *S. salmonicida* [64] (Figure 5). Sulfide dehydrogenase has been proposed to be part of fermentation of organic compounds in archaea with sulfur as the electron acceptor [65], [66]. It may be that this bacterial acquisition has a similar role in diplomonads by oxidation of NADPH to NADP^+^ (Figure 5). Here we identified two additional enzymes, cysteine synthase and serine *O*-acetyltransferase which enable the *S. salmonicida* to biosynthesize cysteine from sulfur and serine (Figure 5). Consequently, in the presence of sulfur or sulfide the parasite is not dependent on a cysteine-rich diet for the synthesis of key enzymes containing iron-sulfur clusters. However, these key enzymes are sensitive to oxygen damage.

### An expanded repertoire of genes involved in oxidative stress response

The conventional enzymes for oxidative stress response, superoxide dismutase, catalase and glutathione peroxidase have not been found in *G. intestinalis* neither using experimental [67] nor bioinformatic approaches [10]. Instead an O_2_-scavaging NADH oxidase [48], [67], [68], superoxide reductase [69] and flavodiiron proteins [70] have been found to be involved in the antioxidative response. *S. salmonicida* causes systemic infections and thus needs a more efficient oxygen scavenging system than *Giardia*. Experimental data suggest that *S. vortens* has an elaborate system that consumes O_2_ for several hours in the absence of added substrates [71]. We indeed identified many more genes involved in protection against reactive oxygen species (ROS) in *S. salmonicida* than in *G. intestinalis* (Table 3). We cannot find any enzymes for glutathione synthesis and recycling in *S. salmonicida*, suggesting that cysteine is the major intracellular thiol, as in *Giardia* and *Entamoeba histolytica*. Future experiments will show if expression of the cysteine-rich CRMP proteins in external and internal membranes (Figure S9) is important in ROS protection, similar to the cysteine-rich metallothioneins in mammalian cells [72]. The importance of the antioxidative response for *S. salmonicida* is underscored by the redundancy of oxygen detoxification mechanisms and the presence of multiple orthologs for many of the proteins (Table 3, Figure S9). Many of the enzymes have bacterial origin and can also be found in *E. histolytica* and *T. vaginalis* [8], [9], [73]. However, the nitric oxide protecting giardial enzyme flavohemoglobin [74] could not be found in the *S. salmonicida* genome. The amino acid methionine (Met) can be oxidized to methionine sulfoxide (MetO) but methionine sulfoxide reductases A (MsrA) and B (MsrB) reduce MetO back to Met, reactivating the oxidized proteins (Figure S9). We detected two MsrA and two MsrB genes in the *S. salmonicida* genome (Table 3), showing that these processes are active in the parasite. Interestingly, certain forms of MRS contain redox-active selenocysteine residues and it is possible that the *S. salmonicida* MRS proteins are selenocysteine proteins. This analysis shows that *S. salmonicida* has an extensive oxygen detoxification system, well-adapted for coping with changing O_2_-levels during infection and transmission.

**Table 3:** Enzymes involved in the oxidative stress response.

Protein	#Spiro	#*Giardia*	Function
FAD/FMN dependent oxidoreductase	3	1	O_2_ to O_2_−
NADH oxidase	4	1	O_2_ to O_2_−
NADPH oxidoreductase	3	1	O_2_ to O_2_−
Nitroreductase	3	1	O_2_ to O_2_−
A-type flavoprotein	7	1	O_2_ to H_2_O
Superoxide reductase	1	1	O_2_− to H_2_O_2_
Hybrid cluster protein	2	1	H_2_O_2_ to H_2_O
Peroxiredoxin	4	3	H_2_O_2_ to H_2_O
Protein containing alkyl hydroperoxide reductase	1	3	H_2_O_2_ to H_2_O
Rubrerythrin 1	5	0	H_2_O_2_ to H_2_O
Peptide methionine sulfoxide reductase MsrA	2	1	repair of oxidative damaged proteins
Peptide methionine sulfoxide reductase MsrB	2	1	repair of oxidative damaged proteins

### Conclusions

The analyses of the *S. salmonicida* genome have provided insights into the biology of this fish parasite. *S. salmonicida* is capable of infecting a large number of different tissues, which have different micro environments, and thereby causing systemic infections. Our analyses have revealed an organism adapted to such fluctuating environments. More regulatory elements, for example putative promoters in many of the genes in the genomes, were found in *S. salmonicida* compared to *G. intestinalis*, suggesting that the fish parasite has a larger potential for regulation on the transcriptional level. The fish parasite encodes enzymes for several more carbohydrates and amino acids for energy production than *G. intestinalis*. Efficient transcriptional regulation of these enzymes may enable *S. salmonicida* to utilise different metabolites during infection of various tissues. The oxygen levels are fluctuating during systematic infections. *S. salmonicida* has a larger number of genes involved in oxidative stress response compared to *G. intestinalis*. The presence of these proteins probably enables *S. salmonicida* to use oxygen-sensitive iron-sulfur cluster containing enzymes for energy production throughout the infection.

The bioinformatic and functional studies indicate that *S. salmonicida* spread between hosts via a cyst stage, although we were unable to encyst the parasite *in vitro*. The conservation of the encystation genes between *G. intestinalis* and *S. salmonicida* suggests that this process was present in the diplomonad ancestor. Both characterized diplomonads have large repertoires of cysteine-rich proteins. The cluster analysis indicated that these protein families are very divergent between the two diplomonads. A subset of the cysteine-rich proteins is used for antigenic variation within *G. intestinalis* and a similar role in *S. salmonicida* appears likely. If so, the mechanisms for antigenic variation are probably rather different in the two diplomonads. Accordingly, convergent evolution, rather than shared ancestry, may have resulted in similar functions for cysteine-rich proteins in *G. intestinalis* and *S. salmonicida*. Thus, the question if the ancestral diplomonad was a free-living organism or a parasite remains open.

Our analyses have uncovered large functional differences within the group diplomonads which provide insights into the flexibility of eukaryotic genomes. We believe that the combination of a draft genome with high-quality annotation and the ability to perform functional studies could turn *S. salmonicida* into a powerful model organism. Not only for comparative studies to the important human parasite *G. intestinalis*, but also for eukaryotes in general.

## Materials and Methods

### Material and sequencing

*S. salmonicida* (ATCC 50377), previously known as *S. barkhanus* [17], was isolated from a muscle abscess in Atlantic salmon grown in Vesterålen Sea in northern Norway. Cells were obtained from American Type Culture Collection (ATCC) and grown in axenic culture following the ATCC protocol. *S. salmonicida* was cultivated in LYI media in tightly capped slanted culture tubes (Nunc) at 16°C according to reference [20]. *G. intestinalis* WB/C6 (ATCC 50803) was cultivated according to reference [75] in TYDK media in tightly capped slanted culture tubes at 37°C. Total genomic DNA was isolated from trophozoites using standard methods. Total RNA was harvested from two batches of trophozoites exponential and stationary stages of growth using standard methods. Amplification with PCR primers specific to bacterial ribosomal RNA indicated no signs of bacterial contamination in the cultures. Equal amounts of total RNA from the two growth stages were pooled and mRNA was isolated using polyA-selection.

Total genomic DNA was sequenced using a Genome Sequencer FLX with GS FLX Titanium series reagents, one run with single shotgun reads and one run from a sequencing library with 3 kbp inserts, yielded 454 reads of 55× genome coverage. The genomic DNA was also sequenced with the Illumina Genome Analyzer IIx instrument, one run with paired-end reads with 100 bp in read length and pairs are 350 bp apart, which yielded Illumina reads of over 200× coverage. The same Illumina instrument was used to sequence *S. salmonicida* mRNA, with paired end reads with insert sizes of 175 bp. Raw DNA and RNA sequence reads are archived at NCBI Sequence Read Archive (SRA) under accession number SRA091283.

### Optical mapping

*S. salmonicida* cells (10^9^ cells) were harvested by chilling on ice followed by pelleting at 500× *g*, 5 min, and 4°C. The cells were washed two times with 10 pellet volumes of PBS. The resulting cell pellet was resuspended in 500 µl 200 mM NaCl, 100 mM EDTA, 10 mM Tris pH 7.2. The suspension was combined with 1% InCert agarose (Cat. No. 50121, Lonza) prepared in ddH_2_O and 100 µl plugs were moulded by incubation at 4°C for 30 min. Cells were lysed by incubating the plugs in 5 mL NDSK solution (1% *N*-lauroylsarcosine, 2 mg/ml Proteinase K in 0.5 M EDTA pH 9.5) at 50°C for 8 h in an upright 50 mL Falcon tube. After 8 h the NDSK solution was replaced by of 5 mL fresh NDSK solution and the plugs were incubated another 18 h. The final lysed nearly transparent plugs were stored in 0.5 M EDTA, pH 9.5 and shipped to OpGen for optical map determination employing the *Nhe*I restriction enzyme. MapSolver v3.2.0 provided by OpGen was used to map assembly sequences to the optical maps.

### Genome assembly

Celera Assembler (CA) v6.0 [76] was used to generate the selected genome assembly using 454 single and mate pair reads. The Illumina DNA reads were mapped to 454 assembly using BWA v0.5.9 [77] and Nesoni v0.40 (http://bioinformatics.net.au/software.nesoni.shtml) was used to correct 454 homopolymer errors based on the mapped bam file. The ribosomal RNAs were not present in the initial assembly, likely due to high coverage. They were then found in one 5.8 kb degenerate contig assembled with Celera Assembler by searching against Rfam 10.0 [78] using infernal v1.0.2 [79]. This extra contig were then included in the final assembly. The degenerate contigs were also used to search against the UniProt database using BLAST [80]. No biologically meaningful data were found in these contigs. Further details of the genome assembly are found in Protocol S1.

This Whole Genome Shotgun project has been deposited at DDBJ/EMBL/GenBank under the accession AUWU00000000. The version described in this paper is version AUWU01000000. The data will also be included in a 2014 release of GiardiaDB (http://giardiadb.org).

### Repeat detection

RepeatMasker version open-3.3.0 (http://www.repeatmasker.org/) was used to screen genome repeats. It was run with default settings and sequence comparison in RepeatMasker was performed by the program cross_match version 1.080812. RepeatMasker library used was RM database version 20110920 with RepBase Update 20110920.

### Heterozygosity estimation

Samtools [81] mpileup with B flag was used to generate pileup file from Illumina DNA reads mapped bam file. SNP sites were called in positions of base coverage more than 20 reads with an alternative base in more than 10% of the reads.

### Genome annotation

An in-house annotation pipeline was implemented to annotate the genome. The annotation pipeline consists of structural and functional annotation. For the structural annotation, EvidenceModeler (EVM) r03062010 [82] was used to combine *ab initio* gene predictions, domain information and transcript alignments in weighted manner to arrive at a consensus gene structure. GlimmerHMM v3.0.1 [83], Prodigal v2.50 [84] and Glimmer3 v.3.02 [85] were the gene prediction programs used. Among them, GlimmerHMM performed best and was weighted highest. Domain information was from Pfam 25.0 [86] and TIGRFAM 10.0 [87] hits using HMMER3 3.0 (http://hmmer.janelia.org/). Transcript alignments include RNA-Seq reads as well as the *Spironucleus* ESTs from dbEST were mapped to the draft assembly. RNA-Seq mapping was done by BWA [77] and EST mapping was done by BLAST. RNA-Seq was weighted most among all the information used. The consensus genes from EVM were then functionally annotated using BLAST results against UniprotKB 20111005 [88] as well as Pfam and TIGRFAM domain hits. Both the functional and structural annotations were then inspected manually with Artemis [89], and adjustments were done to improve the quality of the annotation. More details of the annotation pipeline are found in the Protocol S1.

### Shared and unique genes

The *G. intestinalis* genome refers to the genome from isolate WB [10] in the context of this paper unless otherwise stated. The *G. intestinalis* genome sequence and gene annotation were downloaded from *Giardia*DB 3.1 [90].

OrthoMCL v2.0.2 [91] was run with match cutoff of 50% and e-value cutoff of 1e-10, which resulted in 1349 shared core groups between *S. salmonicida* and *G. intestinalis* including 1718 genes from *S. salmonicida* and 1431 genes from *G. intestinalis*. On top of the OrthoMCL grouping, a pair of genes is considered to be shared between the two species if reciprocal BLAST hits have e-value <1e-03. This approach adds 1550 more *S. salmonicida* genes and 1658 more *G. intestinalis* genes into the shared pool. The rest of the genes were then considered to be unique to each other, which gives 4799 unique *S. salmonicida* genes compared to *G. intestinalis* and 2812 unique *G. intestinalis* genes. The 4799 *S. salmonicida* genes were then used in searches against UniProt KB 20130403 database. The same e-value cutoff was used to estimate *S. salmonicida* unique genes in comparison to all available sequenced genomes. Protein identities were extracted from the reciprocal BLAST results between *S. salmonicida* and *G. intestinalis*. 1147 OrthoMCL orthologous groups with only one member from each species were included in the analysis.

Synteny similarity between *G. intestinalis* and *S. salmonicida* was studied using the 1349 shared core groups based on a sliding window approach. Only homologous groups with less than five members in each species were included to reduce the noise from large protein families. We used a sliding window size of 20 kbp with step size of 1 kbp. Any window containing genes from at least three different homologous groups in both species were indicated and manually examined in ACT [92]. Among the 230 regions indicated, we failed to see any promising synteny blocks between the two species.

### Protein kinases

Protein kinases were identified in combination of three different approaches. 129 genes were found to contain significant Pfam Pkinase domain (PF00069) (score >25). OrthoMCL [91] gene clusters shared between *S. salmonicida* and *G. intestinalis* was used to annotate 67 protein kinases, adding 7 extra kinases.

Protein kinases are categorized into group, family and subfamily. To assign all the protein kinases into group, family and subfamily, we constructed HMM profiles for different group, family and subfamily domains using the alignment files downloaded from the Kinase.com database (http://kinase.com/). Hmmbuild from HMMER 3.0 (http://hmmer.janelia.org/) was used to build HMM profiles from the alignments; hmmsearch to search the protein sequences against HMM profiles. Cutoff score 40 was used for the proteins with Pkinase domain, and score 50 was used for the rest of proteins. This annotated 137 protein kinases, adding 3 extra. The final description of the 138 protein kinases were decided in combination of orthology and HMMER results with manual efforts, and followed name convention in *Giardia*. *G. intestinalis* protein kinase annotations were taken from reference [24].

### tRNAs and rRNAs

tRNAs were predicted by tRNAScan-SE v1.23 [93]. The most sensitive co-variance model was used. 5S, 18S and 28S rRNAs were predicted with RNAmmer v1.2 [94]. 5.8S were predicted by similarity search against Rfam 10.0 [78] using infernal v1.0.2 [79]. 5.8S and 28S overlapped in the initial prediction, and the 28S start was then adjusted according to alignment to other annotated 28S in NCBI.

### Introns

Potential introns were validated by PCR using genomic DNA or cDNA as template, and the PCR products were sequenced with Sanger sequencing. The conserved AC-repeat motif in the intron was also used to search in the whole genome in attempt to look for potential introns or split introns, and all the potential cases were inspected and tested experimentally. To search for split introns, we collected the RNA-Seq reads which aligned to two different positions in the genome for inspection. However, due to the abundance of chimeric reads from sequencing, it was difficult to identify the true signal of split introns, and we did not see any obvious case which could indicate a split intron.

### Promoters

MEME suite v4.8.1 [95] was used for promoter motif analysis. A maximum of 400 bp upstream of annotated genes (including 3′ partial genes) were used in search of potential promoter motifs using MEME. If the intergenic region was shorter than 400 bp, the longest possible sequence was used; if the intergenic regions were shorter than 8 bp, the sequence was ignored. 8208 promoter sequences were used to search for motifs. MEME was set to search for 10 most likely motifs with sizes from 6 bp to 30 bp. FIMO was then used to search in *S. vortens*, *G. intestinalis* and *T. vaginalis* promoter regions for similar motifs. *T. vaginalis* sequences and annotations were from TrichDB 1.3 and *S. vortens* data were from JGI (http://www.jgi.doe.gov/). BLAST searches were used to identify 1726 *S. vortens* homologs to 1253 *S. salmonicida* genes with BLASTX e-value <1e-10, >60% alignment match, <20% length difference, and with proper start and stop codon.

MEME was used to search for promoter motifs upstream of 19 putative *S. salmonicida* cyst-related genes (3 identical CWPs were excluded).

### Gene expression levels

Expression level was measured by mapped RNA-Seq reads in term of FPKM (Fragments Per Kilobase of transcript per Million mapped reads). Cufflinks v2.0.2 [96] was used to calculate the FPKM. The reference annotation file was provided to estimate the expression level of the annotated genes.

### Cloning and heterologous expression in *G. intestinalis* of a *S. salmonicida* cyst wall protein

The fusion of the CWP-1 promoter of *G. intestinalis* and the cyst wall protein was constructed by the creation of *Eco*RV site in the N-terminal of the SS50377_15904 gene. The mutated base is shown in bold font in the primer sequences below. The introduction of this unique restriction site did not alter the resulting amino acid sequence of the SS50377_15904 gene and created a seamless fusion to the CWP-1 promoter. The *S. salmonicida* cyst wall protein (SS50377_15904) was amplified by PCR from *S. salmonicida* genomic DNA as described in [20] using primers CWPE-F-EcoRV CCCGATATCTATCCTGGCAGTCCTCACACAGC and CWP-E-R CCCGCGGCCGCTGTCTAACGTAGACGCCGCAGTC. The *G. intestinalis* CWP-1 promoter was amplified by PCR from genomic DNA of *G. intestinalis* isolate WB/C6 using primers CWP1-P-HindIII CCCAAGCTTCAGAGGCATTGGACTTTGTCATG and CWP1-P-EcoRV CCCGATATCATCCCTGATATTTTATTTCTGTGTTTCTTG. Underlined sequences denote introduced restriction enzyme sites. The SS50377_15904 and CWP1-P PCR products were gel purified by QIAquick Gel Extraction kit (Qiagen) and digested using *Hind*III and *Eco*RV and *Eco*RV and *Not*I. All restriction enzymes were of the FastDigest type from Fermentas. The fragments were purified by QIAquick PCR purification kit (Qiagen) and ligated using T4 DNA ligase into digested (*Hind*III and *Not*I) and FastAP (Fermentas) dephosphorylated PHA-5 vector (Jerlström-Hultqvist, unpublished results). The vector was transformed into DH5α *E. coli* cells and correct clones were identified by restriction digestion of purified plasmids. The sequence of identified clones was verified by Sanger sequencing.

### Cloning and immunofluorescence of *S. salmonicida* cysteine-rich proteins

*S. salmonicida* cysteine-rich proteins were amplified by PCR from *S. salmonicida* genomic DNA using primers-pairs 17215-F ATATGCTAGCGCTTCATTGTAACATTTAATAAATTATCTCGCACATC 17215-R TATGCGGCCGCCATTGGATTTTTGAACCATTCTACGACATT 18013-F ATATGCTAGCTAAACTTCGTATGATATGCAATAAACGGC 18013-R TATGCGGCCGCCACCAAAGTACGTTACTAAGTGGCTCA 18923-F ATATGCTAGCTAATTATGGTTCTGCAGTGAGGAGTG 18923-R TATGCGGCCGCCACTGTTACTCCACTCTCTTCTTAGCC respectively as described previously [20]. The PCR products were gel-purified, digested by *Nhe*I and *Not*I and inserted into pSpiro-PAC-3×HA-C vector linearized with the above mentioned enzymes. Correct plasmids were recovered and sequences verified as described above.

### Transfection of *G. intestinalis* or *S. salmonicida*

Plasmid DNA for transfection of *S. salmonicida* or *G. intestinalis* was prepared as described in reference [20]. Culture and transfection of *S. salmonicida* and *G. intestinalis* WB/C6 were according to reference [20] and reference [75], respectively. Transfectants of either organism were selected and maintained using 50 µg/ml puromycin (A.G Scientific). The *G. intestinalis* transfectants were induced to encyst by replacing the normal growth media of 70–80% confluent cultures with encystation media with pH 7.8 and 1.25 mg/ml of bovine bile. *In vitro* generated cysts were harvested 48 h post induction by centrifugation at 500× *g* and kept in water at 4°C for 24 h.

### Immunofluorescence labelling and western blot of cells

*S. salmonicida* or encysting *G. intestinalis* transfectants cells (0, 6, 22 and 48 h post induction) as well as water-treated cysts were collected for immunofluorescence and Western blot as described [20], [75]. Cells were fixed, permeabilised and blocked [20]. The cells were stained either alone or with appropriate combinations anti-HA-Alexa Fluor 488 antibody (A488-101L, Covance; 1∶250 dilution), anti-CWP1-FITC antibody (Waterborne Inc.; 1∶20 dilution) or rabbit monoclonal HA-tag (C29F4) antibody (Cat. No: 3724, Cell signalling technologies; 1∶1600 dilution). The rabbit monoclonal was detected using goat anti-rabbit conjugated to AlexaFluor 594 (A-11037, Invitrogen; 1∶250 dilution). The stained cells were mounted in VectaShield with DAPI (Cat. No: H-1200, Vector Laboratories) and viewed using either a Zeiss Axioplan 2 epifluoresence microscope or a Zeiss 510 laser scanning confocal microscope. AxioVision LE 4.8.2.0 or Zen 2011 v7.0.0.285 (Carl Zeiss GmBH) was used to process images.

Cells carrying CRMP-1 constructs were fixed using 2% PFA, blocked with 2% BSA and stained using mouse anti-HA-Alexa Fluor 488 (1∶250), mounted in VectaShield medium containing DAPI and viewed using a Zeiss 510 laser scanning confocal microscope.

### Proteases

Peptidase and peptidase inhibitor sequences were obtained from the MEROPS database [41] (release 9.6). Predicted *S. salmonicida* genes were used to search against all the peptidase protein sequences using BLASTP with e-value cutoff of 1e-03, and to search against Pfam domains (v25.0) [86] using HMMER 3.0 (http://hmmer.janelia.org/). A gene was assigned as a peptidase if any of the following three conditions satisfied: if a gene was supported by both a BLAST hit with e-value <1e-03 and Pfam peptidase domain hit with score >20; if a gene was supported by a good domain hit with score >50; if a gene was supported by a good BLAST hit with e-value <1e-10 and was already manually annotated as protease. The catalytic type and protease family were predicted in accordance with the classification in MEROPS. The same strategy was used to assign the proteases of *G. intestinalis*. The assignment results were similar to the information from the MEROPS database.

### Cysteine-rich proteins

We aligned proteins with more than 5% cysteine using MUSCLE (v3.8.31) [97], and detected transmembrane domains, a conserved [KR][KR]X[KR][KR] signature motif, and repeated motifs of CXXC and CXC. Three types of cysteine-rich proteins were defined depending on the presence and absence of the transmembrane domain and sequence motifs within the cysteine-rich proteins. We used gene networks [98] to investigate the diversity of those three types together with the VSPs, HCMPs and HCPs from *G. intestinalis*. Python igraph was used to plot the gene networks. Each node represents a gene sequence, and sequences are connected if they share a sequence homology which was determined by the reciprocal BLASTP e-value <1e-05.

### Analysis of pathways

Pathways were predicted using KEGG Automatic Annotation Server (KAAS) [99] with default cutoff 60. The predictions for *S. salmonicida* and *G. intestinalis* were compared. The differences were manually examined and expanded using the literature on *G. intestinalis* metabolism [10], [12].

### Transporter proteins

To identify potential transporter proteins in *S. salmonicida*, we started from proteins with at least one transmembrane domain predicted by TMHMM v2.0 [100]. Transporter family information was downloaded from the Transporter classification database (TCDB; http://www.tcdb.org/). Transporters were assigned using a combination of BLASTP hits with <1e-03 against TCDB collections of transporters, and Pfam domain hits with score >25. The same strategy was applied to assign *G. intestinalis* transporters. The transporter assignments were then verified with orthologous groups between the two organisms and proved to be consistent.

## Supporting Information

Figure S1 Histogram of the protein identities. Histogram of the protein identities from the 1147 orthologous pairs between *S. salmonicida* and *G. intestinalis*. Green line indicates the average protein identity.
(PDF)

Figure S2 Alignment of four introns. MUSCLE v3.8.31 [97] was used to align the introns. The AC-repeat motif is underlined. SS50377_17358 marked with * is the gene where the intron failed to be verified using RT-PCR. SS50377_16979 encodes ribosomal protein L30, SS50377_16134 ribosomal protein S24, and SS50377_18398 and SS50377_17358 two hypothetical proteins.
(PDF)

Figure S3 Putative signal peptides characteristics within a group of sugar transporter. SignalP v4.1 [30] was used to analyse signal peptides. C-score (raw cleavage site score) and the S-score (signal peptide score) from the software are shown with red and green lines respectively. Dash lines indicate the score threshold used to claim a positive signal peptide. Black vertical bar indicates the cleavage site if predicted.
(PDF)

Figure S4 Sequence patterns around diplomonad start codons. A. Sequence logo of C-rich motif found in *S. vortens*. B. Sequence logo around the *S. salmonicida* start codon. C and D. AT contents in percentage of the 20 bp C-terminus of all the genes with their 120 bp promoter regions drawn with window size of 3 and step size of 2 for *S. vortens* and *G. intestinalis*, respectively. Green line indicates the average AT percentage.
(PDF)

Figure S5 3′ untranslated regions and polyadenylation machinery. A. Sequence logo around the stop codon. B. Polyadenlylation machinery in *S. salmonicida* and *G. intestinalis*. Numbers refer to protein IDs.
(PDF)

Figure S6 Putative signal peptides characteristics within cyst wall proteins. SignalP v4.1 [30] was used to analyse signal peptides. C-score (raw cleavage site score) and the S-score (signal peptide score) from the software were shown with red and green lines respectively. Dash line indicates the score threshold used to claim a positive signal peptide. Black vertical bar indicates the cleavage site if predicted.
(PDF)

Figure S7 Alignment of the CRMP-1 motif. MUSCLE v3.8.31 [97] was used to align 125 CRMP-1 complete amino acid sequences. Jalview v14.0 [101] was used to visualize and filter the alignment. Left part (2034 positions) of the alignment was removed leaving only C-terminus conserved region (79 positions). There are 66 sequences left after removing redundancy with threshold 95.
(PDF)

Figure S8 Metabolic reconstruction of *G. intestinalis* and *S. salmonicida*. Black arrows indicate enzymatic functions present in both *G. intestinalis* and *S. salmonicida*, green arrows indicate functions not detected in *G. intestinalis* and red arrows indicate functions not detected in *S. salmonicida*.
(PDF)

Figure S9 Oxidative stress response in *S. salmonicida*. Schematic representation of the function of the proteins listed in Table 3. *S. salmonicida* enzymes are shown in blue. Italic font indicates that putatively proteins performing these functions were identified.
(PDF)

Protocol S1 A detailed description of the sequencing data, genome assembly and annotation.
(PDF)

Table S1 *S. salmonicida* proteins without homologs in *G. intestinalis*.
(PDF)

Table S2 The kinome of *G. intestinalis* and *S. salmonicida*.
(PDF)

Table S3 Identified protease families in *G. intestinalis* and *S. salmonicida*.
(PDF)

Table S4 Cysteine-rich proteins in *S. salmonicida*.
(PDF)

Table S5 Identified transporter families in *G. intestinalis* and *S. salmonicida*.
(PDF)

## References

[1] Adl SM, Simpson AG, Lane CE, Lukes J, Bass D, et al (2012) The revised classification of eukaryotes. J Eukaryot Microbiol 59: 429–514. 23020233 10.1111/j.1550-7408.2012.00644.x PMC3483872

[2] Brugerolle G, Lee JJ (2002) Order Diplomonadida. In: Lee JJ, Leedale GF, Bradbury P, editors. An Illustrated Guide to the Protozoa, 2nd edn. Lawrence, Kansas: Society of Protozoologists. pp. 1125–1135.

[3] Tovar J, León-Avila G, Sánchez LB, Sutak R, Tachezy J, et al (2003) Mitochondrial remnant organelles of *Giardia* function in iron-sulphur protein maturation. Nature 426: 172–176. 14614504 10.1038/nature01945

[4] Jerlström-Hultqvist J, Einarsson E, Xu F, Hjort K, Ek B, et al (2013) Hydrogenosomes in the diplomonad *Spironucleus salmonicida* . Nat Commun 4: 2493. 24042146 10.1038/ncomms3493 PMC3778541

[5] Ramesh MA, Malik SB, Logsdon JM Jr (2005) A phylogenomic inventory of meiotic genes; evidence for sex in *Giardia* and an early eukaryotic origin of meiosis. Curr Biol 15: 185–191. 15668177 10.1016/j.cub.2005.01.003

[6] Cooper MA, Adam RD, Worobey M, Sterling CR (2007) Population genetics provides evidence for recombination in *Giardia* . Curr Biol 17: 1984–1988. 17980591 10.1016/j.cub.2007.10.020

[7] Andersson JO (2012) Double peaks reveal rare diplomonad sex. Trends Parasitol 28: 46–52. 22192817 10.1016/j.pt.2011.11.002

[8] Andersson JO, Sjögren ÅM, Horner DS, Murphy CA, Dyal PL, et al (2007) A genomic survey of the fish parasite *Spironucleus salmonicida* indicates genomic plasticity among diplomonads and significant lateral gene transfer in eukaryote genome evolution. BMC Genomics 8: 51. 17298675 10.1186/1471-2164-8-51 PMC1805757

[9] Andersson JO, Sjögren ÅM, Davis LAM, Embley TM, Roger AJ (2003) Phylogenetic analyses of diplomonad genes reveal frequent lateral gene transfers affecting eukaryotes. Curr Biol 13: 94–104. 12546782 10.1016/s0960-9822(03)00003-4

[10] Morrison HG, McArthur AG, Gillin FD, Aley SB, Adam RD, et al (2007) Genomic minimalism in the early diverging intestinal parasite *Giardia lamblia* . Science 317: 1921–1926. 17901334 10.1126/science.1143837

[11] Upcroft J, Upcroft P (1998) My favorite cell: *Giardia* . Bioessays 20: 256–263. 9631653 10.1002/(SICI)1521-1878(199803)20:3<256::AID-BIES9>3.0.CO;2-P

[12] Adam RD (2001) Biology of *Giardia lamblia* . Clin Microbiol Rev 14: 447–475. 11432808 10.1128/CMR.14.3.447-475.2001 PMC88984

[13] Ankarklev J, Jerlström-Hultqvist J, Ringqvist E, Troell K, Svärd SG (2010) Behind the smile: cell biology and disease mechanisms of *Giardia* species. Nat Rev Microbiol 8: 413–422. 20400969 10.1038/nrmicro2317

[14] Williams CF, Lloyd D, Poynton SL, Jorgensen A, Millet COM, et al (2011) *Spironucleus* species: economically-important fish pathogens and enigmatic single-celled eukaryotes. J Aquac Res Development S2.

[15] Kolisko M, Cepicka I, Hampl V, Leigh J, Roger AJ, et al (2008) Molecular phylogeny of diplomonads and enteromonads based on SSU rRNA, alpha-tubulin and HSP90 genes: implications for the evolutionary history of the double karyomastigont of diplomonads. BMC Evol Biol 8: 205. 18627633 10.1186/1471-2148-8-205 PMC2496913

[16] Kent ML, Ellis J, Fournie JW, Dawe SC, Bagshaw JW, et al (1992) Systemic hexamitid (Protozoa, Diplomonadida) infection in seawater pen-reared Chinook salmon *Oncorhynchus tshawytscha* . Dis Aquat Organ 14: 81–89.

[17] Jørgensen A, Sterud E (2006) The marine pathogenic genotype of *Spironucleus barkhanus* from farmed salmonids redescribed as *Spironucleus salmonicida* n. sp. J Eukaryot Microbiol 53: 531–541. 17123418 10.1111/j.1550-7408.2006.00144.x

[18] Roxström-Lindquist K, Jerlström-Hultqvist J, Jørgensen A, Troell K, Svärd SG, et al (2010) Large genomic differences between the morphologically indistinguishable diplomonads *Spironucleus barkhanus* and *Spironucleus salmonicida* . BMC Genomics 11: 258. 20409319 10.1186/1471-2164-11-258 PMC2874811

[19] Jørgensen A, Torp K, Bjorland MA, Poppe TT (2011) Wild arctic char *Salvelinus alpinus* and trout *Salmo trutta*: hosts and reservoir of the salmonid pathogen *Spironucleus salmonicida* (Diplomonadida; Hexamitidae). Dis Aquat Organ 97: 57–63. 22235595 10.3354/dao02404

[20] Jerlström-Hultqvist J, Einarsson E, Svärd SG (2012) Stable transfection of the diplomonad parasite *Spironucleus salmonicida* . Eukaryot Cell 11: 1353–1361. 22983987 10.1128/EC.00179-12 PMC3486028

[21] Franzén O, Jerlström-Hultqvist J, Castro E, Sherwood E, Ankarklev J, et al (2009) Draft genome sequencing of *Giardia intestinalis* assemblage B isolate GS: are human giardiasis caused by two different species? PLoS Pathog 5 (8) e1000560. 19696920 10.1371/journal.ppat.1000560 PMC2723961

[22] Jerlström-Hultqvist J, Franzén O, Ankarklev J, Xu F, Nohynkova E, et al (2010) Genome analysis and comparative genomics of a *Giardia intestinalis* assemblage E isolate. BMC Genomics 11: 543. 20929575 10.1186/1471-2164-11-543 PMC3091692

[23] Keeling PJ, Doolittle WF (1997) Widespread and ancient distribution of a noncanonical genetic code in diplomonads. Mol Biol Evol 14: 895–901. 9287422 10.1093/oxfordjournals.molbev.a025832

[24] Manning G, Reiner DS, Lauwaet T, Dacre M, Smith A, et al (2011) The minimal kinome of *Giardia lamblia* illuminates early kinase evolution and unique parasite biology. Genome Biol 12: R66. 21787419 10.1186/gb-2011-12-7-r66 PMC3218828

[25] O'Connell MJ, Krien MJ, Hunter T (2003) Never say never. The NIMA-related protein kinases in mitotic control. Trends Cell Biol 13: 221–228. 12742165 10.1016/s0962-8924(03)00056-4

[26] Collins L, Penny D (2005) Complex spliceosomal organization ancestral to extant eukaryotes. Mol Biol Evol 22: 1053–1066. 15659557 10.1093/molbev/msi091

[27] Franzen O, Jerlström-Hultqvist J, Einarsson E, Ankarklev J, Ferella M, et al (2013) Transcriptome profiling of *Giardia intestinalis* using strand-specific RNA-seq. PLoS Comput Biol 9: e1003000. 23555231 10.1371/journal.pcbi.1003000 PMC3610916

[28] Kamikawa R, Inagaki Y, Tokoro M, Roger AJ, Hashimoto T (2011) Split introns in the genome of *Giardia intestinalis* are excised by spliceosome-mediated trans-splicing. Curr Biol 21: 311–315. 21315596 10.1016/j.cub.2011.01.025

[29] Carlton JM, Hirt RP, Silva JC, Delcher AL, Schatz M, et al (2007) Draft genome sequence of the sexually transmitted pathogen *Trichomonas vaginalis* . Science 315: 207–212. 17218520 10.1126/science.1132894 PMC2080659

[30] Petersen TN, Brunak S, von Heijne G, Nielsen H (2011) SignalP 4.0: discriminating signal peptides from transmembrane regions. Nat Methods 8: 785–786. 21959131 10.1038/nmeth.1701

[31] Akopian D, Shen K, Zhang X, Shan SO (2013) Signal recognition particle: an essential protein-targeting machine. Annu Rev Biochem 82: 693–721. 23414305 10.1146/annurev-biochem-072711-164732 PMC3805129

[32] Teodorovic S, Walls CD, Elmendorf HG (2007) Bidirectional transcription is an inherent feature of *Giardia lamblia* promoters and contributes to an abundance of sterile antisense transcripts throughout the genome. Nucleic Acids Res 35: 2544–2553. 17403692 10.1093/nar/gkm105 PMC1885649

[33] Best AA, Morrison HG, McArthur AG, Sogin ML, Olsen GJ (2004) Evolution of eukaryotic transcription: insights from the genome of *Giardia lamblia* . Genome Res 14: 1537–1547. 15289474 10.1101/gr.2256604 PMC509262

[34] Iyer LM, Anantharaman V, Wolf MY, Aravind L (2008) Comparative genomics of transcription factors and chromatin proteins in parasitic protists and other eukaryotes. Int J Parasitol 38: 1–31. 17949725 10.1016/j.ijpara.2007.07.018

[35] Fuentes V, Barrera G, Sanchez J, Hernandez R, Lopez-Villasenor I (2012) Functional analysis of sequence motifs involved in the polyadenylation of *Trichomonas vaginalis* mRNAs. Eukaryot Cell 11: 725–734. 22467744 10.1128/EC.05322-11 PMC3370463

[36] Williams CF, Vacca AR, Lloyd D, Schelkle B, Cable J (2013) Non-invasive investigation of *Spironucleus vortens* transmission in freshwater angelfish *Pterophyllum scalare* . Dis Aquat Organ 105: 211–223. 23999705 10.3354/dao02618

[37] Januschka MM, Erlandsen SL, Bemrick WJ, Schupp DG, Feely DE (1988) A comparison of *Giardia microti* and *Spironucleus muris* cysts in the vole: an immunocytochemical, light, and electron microscopic study. J Parasitol 74: 452–458. 3288741

[38] Wood AM, Smith HV (2005) Spironucleosis (Hexamitiasis, Hexamitosis) in the ring-necked pheasant (*Phasianus colchicus*): detection of cysts and description of *Spironucleus meleagridis* in stained smears. Avian Dis 49: 138–143. 15839427 10.1637/7250-080204R

[39] Morf L, Spycher C, Rehrauer H, Fournier CA, Morrison HG, et al (2010) The transcriptional response to encystation stimuli in *Giardia lamblia* is restricted to a small set of genes. Eukaryot Cell 9: 1566–1576. 20693303 10.1128/EC.00100-10 PMC2950437

[40] Konrad C, Spycher C, Hehl AB (2010) Selective condensation drives partitioning and sequential secretion of cyst wall proteins in differentiating *Giardia lamblia* . PLoS Pathog 6: e1000835. 20386711 10.1371/journal.ppat.1000835 PMC2851657

[41] Rawlings ND, Morton FR, Kok CY, Kong J, Barrett AJ (2008) MEROPS: the peptidase database. Nucleic Acids Res 36: D320–325. 17991683 10.1093/nar/gkm954 PMC2238837

[42] Sajid M, McKerrow JH (2002) Cysteine proteases of parasitic organ. Mol Biochem Parasitol 120: 1–21. 11849701 10.1016/s0166-6851(01)00438-8

[43] Adam RD, Nigam A, Seshadri V, Martens CA, Farneth GA, et al (2010) The *Giardia lamblia vsp* gene repertoire: characteristics, genomic organization, and evolution. BMC Genomics 11: 424. 20618957 10.1186/1471-2164-11-424 PMC2996952

[44] Nash TE, Banks SM, Alling DW, Merritt JW Jr, Conrad JT (1990) Frequency of variant antigens in *Giardia lamblia* . Exp Parasitol 71: 415–421. 1699782 10.1016/0014-4894(90)90067-m

[45] Pagny S, Lerouge P, Faye L, Gomord V (1999) Signals and mechanisms for protein retention in the endoplasmic reticulum. J Exp Bot 50: 157–164.

[46] Davids BJ, Reiner DS, Birkeland SR, Preheim SP, Cipriano MJ, et al (2006) A new family of giardial cysteine-rich non-VSP protein genes and a novel cyst protein. PLoS ONE 1: e44. 17183673 10.1371/journal.pone.0000044 PMC1762436

[47] Prucca CG, Slavin I, Quiroga R, Elias EV, Rivero FD, et al (2008) Antigenic variation in *Giardia lamblia* is regulated by RNA interference. Nature 456: 750–754. 19079052 10.1038/nature07585

[48] Nixon JEJ, Wang A, Field J, Morrison HG, McArthur AG, et al (2002) Evidence for lateral transfer of genes encoding ferredoxins, nitroreductases, NADH oxidase, and alcohol dehydrogenase 3 from anaerobic prokaryotes to *Giardia lamblia* and *Entamoeba histolytica* . Eukaryot Cell 1: 181–190. 12455953 10.1128/EC.1.2.181-190.2002 PMC118039

[49] Law CJ, Maloney PC, Wang DN (2008) Ins and outs of major facilitator superfamily antiporters. Annu Rev Microbiol 62: 289–305. 18537473 10.1146/annurev.micro.61.080706.093329 PMC2612782

[50] Davidson AL, Dassa E, Orelle C, Chen J (2008) Structure, function, and evolution of bacterial ATP-binding cassette systems. Microbiol Mol Biol Rev 72: 317–364. 18535149 10.1128/MMBR.00031-07 PMC2415747

[51] Young GB, Jack DL, Smith DW, Saier MH Jr (1999) The amino acid/auxin: proton symport permease family. Biochim Biophys Acta 1415: 306–322. 9889387 10.1016/s0005-2736(98)00196-5

[52] Schofield PJ, Costello M, Edwards MR, O'Sullivan WJ (1990) The arginine dihydrolase pathway is present in *Giardia intestinalis* . Int J Parasitol 20: 697–699. 2228433 10.1016/0020-7519(90)90133-8

[53] Yarlett N, Martinez MP, Moharrami MA, Tachezy J (1996) The contribution of the arginine dihydrolase pathway to energy metabolism by *Trichomonas vaginalis* . Mol Biochem Parasitol 78: 117–125. 8813682 10.1016/s0166-6851(96)02616-3

[54] Lacourciere GM, Mihara H, Kurihara T, Esaki N, Stadtman TC (2000) *Escherichia coli* NifS-like proteins provide selenium in the pathway for the biosynthesis of selenophosphate. J Biol Chem 275: 23769–23773. 10829016 10.1074/jbc.M000926200

[55] Lacourciere GM (2002) Selenium is mobilized *in vivo* from free selenocysteine and is incorporated specifically into formate dehydrogenase H and tRNA nucleosides. J Bacteriol 184: 1940–1946. 11889101 10.1128/JB.184.7.1940-1946.2002 PMC134910

[56] Haft DH, Self WT (2008) Orphan SelD proteins and selenium-dependent molybdenum hydroxylases. Biol Direct 3: 4. 18289380 10.1186/1745-6150-3-4 PMC2276186

[57] Zhang Y, Turanov AA, Hatfield DL, Gladyshev VN (2008) *In silico* identification of genes involved in selenium metabolism: evidence for a third selenium utilization trait. BMC Genomics 9: 251. 18510720 10.1186/1471-2164-9-251 PMC2432076

[58] Kopriva S, Patron NJ, Keeling P, Leustek T (2008) Phylogenetic analysis of sulfate assimilation and cysteine biosynthesis in phototrophic organisms. In: Hell R, Dahl C, Knaff DB, Leustek T, editors. Sulfur Metabolism in Phototrophic Organisms: Springer Netherlands. pp. 31–58.

[59] Takahashi H, Kopriva S, Giordano M, Saito K, Hell R (2011) Sulfur assimilation in photosynthetic organisms: molecular functions and regulations of transporters and assimilatory enzymes. Annu Rev Plant Biol 62: 157–184. 21370978 10.1146/annurev-arplant-042110-103921

[60] Hérbert A, Casaregola S, Beckerich J-M (2011) Biodiversity in sulfurmetabolism in hemiascomycetous yeasts. FEMS Yeast Res 11: 366–378. 21348937 10.1111/j.1567-1364.2011.00725.x

[61] Payne SH, Loomis WF (2006) Retention and loss of amino acid biosynthetic pathways based on analysis of whole-genome sequences. Eukaryot Cell 5: 272–276. 16467468 10.1128/EC.5.2.272-276.2006 PMC1405893

[62] Ma K, Adams MWW (1994) Sulfide dehydrogenase from the hyperthermophilic archaeon *Pyrococcus furiosus*: a new multifunctional enzyme involved in the reduction of elemental sulfur. J Bacteriol 176: 6509–6517. 7961401 10.1128/jb.176.21.6509-6517.1994 PMC197004

[63] Hagen WR, Silva PJ, Amorim MA, Hagedoorn P-L, Wassink H, et al (2000) Novel structure and redox chemistry of the prosthetic groups of the iron-sulfur flavoprotein sulfide dehydrogenase from *Pyrococcus furiosus*; evidence for a [2Fe-2S] cluster with Asp(Cys)3 ligands. J Biol Inorg Chem 5: 527–534. 10968624 10.1007/pl00021452

[64] Andersson JO, Roger AJ (2002) Evolutionary analyses of the small subunit of glutamate synthase: gene order conservation, gene fusions and prokaryote-to-eukaryote lateral gene transfers. Eukaryot Cell 1: 304–310. 12455964 10.1128/EC.1.2.304-310.2002 PMC118040

[65] Bridger SL, Clarkson SM, Stirrett K, DeBarry MB, Lipscomb GL, et al (2011) Deletion strains reveal metabolic roles for key elemental sulfur-responsive proteins in *Pyrococcus furiosus* . J Bacteriol 193: 6498–6504. 21965560 10.1128/JB.05445-11 PMC3232869

[66] Liu Y, Beer LL, Whitman WB (2012) Sulfur metabolism in archaea reveals novel processes. Environ Microbiol 14: 2632–2644. 22626264 10.1111/j.1462-2920.2012.02783.x

[67] Brown DM, Upcroft JA, Upcroft P (1995) Free radical detoxification in *Giardia duodenalis* . Mol Biochem Parasitol 72: 47–56. 8538699 10.1016/0166-6851(95)00065-9

[68] Brown DM, Upcroft JA, Upcroft P (1996) A H_2_O-producing NADH oxidase from the protozoan parasite *Giardia duodenalis* . Eur J Biochem 241: 155–161. 8898901 10.1111/j.1432-1033.1996.0155t.x

[69] Testa F, Mastronicola D, Cabelli DE, Bordi E, Pucillo LP, et al (2011) The superoxide reductase from the early diverging eukaryote *Giardia intestinalis* . Free Radic Biol Med 51: 1567–1574. 21839165 10.1016/j.freeradbiomed.2011.07.017

[70] Vicente JB, Testa F, Mastronicola D, Forte E, Sarti P, et al (2009) Redox properties of the oxygen-detoxifying flavodiiron protein from the human parasite *Giardia intestinalis* . Arch Biochem Biophys 488: 9–13. 19545535 10.1016/j.abb.2009.06.011

[71] Millet CO, Cable J, Lloyd D (2010) The diplomonad fish parasite *Spironucleus vortens* produces hydrogen. J Eukaryot Microbiol 57: 400–404. 20726936 10.1111/j.1550-7408.2010.00499.x

[72] Babula P, Masarik M, Adam V, Eckschlager T, Stiborova M, et al (2012) Mammalian metallothioneins: properties and functions. Metallomics 4: 739–750. 22791193 10.1039/c2mt20081c

[73] Andersson JO, Hirt RP, Foster PG, Roger AJ (2006) Evolution of four gene families with patchy phylogenetic distribution: influx of genes into protist genomes. BMC Evol Biol 6: 27. 16551352 10.1186/1471-2148-6-27 PMC1484493

[74] Mastronicola D, Testa F, Forte E, Bordi E, Pucillo LP, et al (2010) Flavohemoglobin and nitric oxide detoxification in the human protozoan parasite *Giardia intestinalis* . Biochem Biophys Res Commun 399: 654–658. 20691663 10.1016/j.bbrc.2010.07.137

[75] Jerlström-Hultqvist J, Stadelmann B, Birkestedt S, Hellman U, Svärd SG (2012) Plasmid vectors for proteomic analyses in *Giardia*: purification of virulence factors and analysis of the proteasome. Eukaryot Cell 11: 864–873. 22611020 10.1128/EC.00092-12 PMC3416501

[76] Miller JR, Delcher AL, Koren S, Venter E, Walenz BP, et al (2008) Aggressive assembly of pyrosequencing reads with mates. Bioinformatics 24: 2818–2824. 18952627 10.1093/bioinformatics/btn548 PMC2639302

[77] Li H, Durbin R (2009) Fast and accurate short read alignment with Burrows-Wheeler transform. Bioinformatics 25: 1754–1760. 19451168 10.1093/bioinformatics/btp324 PMC2705234

[78] Gardner PP, Daub J, Tate J, Moore BL, Osuch IH, et al (2011) Rfam: Wikipedia, clans and the “decimal” release. Nucleic Acids Res 39: D141–145. 21062808 10.1093/nar/gkq1129 PMC3013711

[79] Nawrocki EP, Kolbe DL, Eddy SR (2009) Infernal 1.0: inference of RNA alignments. Bioinformatics 25: 1335–1337. 19307242 10.1093/bioinformatics/btp157 PMC2732312

[80] Consortium U (2012) Reorganizing the protein space at the Universal Protein Resource (UniProt). Nucleic Acids Res 40: D71–75. 22102590 10.1093/nar/gkr981 PMC3245120

[81] Li H, Handsaker B, Wysoker A, Fennell T, Ruan J, et al (2009) The Sequence Alignment/Map format and SAMtools. Bioinformatics 25: 2078–2079. 19505943 10.1093/bioinformatics/btp352 PMC2723002

[82] Haas BJ, Salzberg SL, Zhu W, Pertea M, Allen JE, et al (2008) Automated eukaryotic gene structure annotation using EVidenceModeler and the Program to Assemble Spliced Alignments. Genome Biol 9: R7. 18190707 10.1186/gb-2008-9-1-r7 PMC2395244

[83] Majoros WH, Pertea M, Salzberg SL (2004) TigrScan and GlimmerHMM: two open source ab initio eukaryotic gene-finders. Bioinformatics 20: 2878–2879. 15145805 10.1093/bioinformatics/bth315

[84] Hyatt D, Chen GL, Locascio PF, Land ML, Larimer FW, et al (2010) Prodigal: prokaryotic gene recognition and translation initiation site identification. BMC Bioinformatics 11: 119. 20211023 10.1186/1471-2105-11-119 PMC2848648

[85] Delcher AL, Bratke KA, Powers EC, Salzberg SL (2007) Identifying bacterial genes and endosymbiont DNA with Glimmer. Bioinformatics 23: 673–679. 17237039 10.1093/bioinformatics/btm009 PMC2387122

[86] Finn RD, Mistry J, Tate J, Coggill P, Heger A, et al (2010) The Pfam protein families database. Nucleic Acids Res 38: D211–222. 19920124 10.1093/nar/gkp985 PMC2808889

[87] Selengut JD, Haft DH, Davidsen T, Ganapathy A, Gwinn-Giglio M, et al (2007) TIGRFAMs and Genome Properties: tools for the assignment of molecular function and biological process in prokaryotic genomes. Nucleic Acids Res 35: D260–264. 17151080 10.1093/nar/gkl1043 PMC1781115

[88] UniProt C (2012) Reorganizing the protein space at the Universal Protein Resource (UniProt). Nucleic Acids Res 40: D71–75. 22102590 10.1093/nar/gkr981 PMC3245120

[89] Rutherford K, Parkhill J, Crook J, Horsnell T, Rice P, et al (2000) Artemis: sequence visualization and annotation. Bioinformatics 16: 944–945. 11120685 10.1093/bioinformatics/16.10.944

[90] Aurrecoechea C, Brestelli J, Brunk BP, Carlton JM, Dommer J, et al (2009) GiardiaDB and TrichDB: integrated genomic resources for the eukaryotic protist pathogens *Giardia lamblia* and *Trichomonas vaginalis* . Nucleic Acids Res 37: D526–530. 18824479 10.1093/nar/gkn631 PMC2686445

[91] Li L, Stoeckert CJ Jr, Roos DS (2003) OrthoMCL: identification of ortholog groups for eukaryotic genomes. Genome Res 13: 2178–2189. 12952885 10.1101/gr.1224503 PMC403725

[92] Carver TJ, Rutherford KM, Berriman M, Rajandream MA, Barrell BG, et al (2005) ACT: the Artemis Comparison Tool. Bioinformatics 21: 3422–3423. 15976072 10.1093/bioinformatics/bti553

[93] Schattner P, Brooks AN, Lowe TM (2005) The tRNAscan-SE, snoscan and snoGPS web servers for the detection of tRNAs and snoRNAs. Nucleic Acids Res 33: W686–689. 15980563 10.1093/nar/gki366 PMC1160127

[94] Lagesen K, Hallin P, Rodland EA, Staerfeldt HH, Rognes T, et al (2007) RNAmmer: consistent and rapid annotation of ribosomal RNA genes. Nucleic Acids Res 35: 3100–3108. 17452365 10.1093/nar/gkm160 PMC1888812

[95] Bailey TL, Elkan C (1994) Fitting a mixture model by expectation maximization to discover motifs in biopolymers. Proc Int Conf Intell Syst Mol Biol 2: 28–36. 7584402

[96] Trapnell C, Williams BA, Pertea G, Mortazavi A, Kwan G, et al (2010) Transcript assembly and quantification by RNA-Seq reveals unannotated transcripts and isoform switching during cell differentiation. Nat Biotechnol 28: 511–515. 20436464 10.1038/nbt.1621 PMC3146043

[97] Edgar RC (2004) MUSCLE: a multiple sequence alignment method with reduced time and space complexity. BMC Bioinformatics 5: 113. 15318951 10.1186/1471-2105-5-113 PMC517706

[98] Beauregard-Racine J, Bicep C, Schliep K, Lopez P, Lapointe FJ, et al (2011) Of woods and webs: possible alternatives to the tree of life for studying genomic fluidity in *E. coli* . Biol Direct 6: 39. 21774799 10.1186/1745-6150-6-39 PMC3160433

[99] Moriya Y, Itoh M, Okuda S, Yoshizawa AC, Kanehisa M (2007) KAAS: an automatic genome annotation and pathway reconstruction server. Nucleic Acids Res 35: W182–185. 17526522 10.1093/nar/gkm321 PMC1933193

[100] Krogh A, Larsson B, von Heijne G, Sonnhammer EL (2001) Predicting transmembrane protein topology with a hidden Markov model: application to complete genomes. J Mol Biol 305: 567–580. 11152613 10.1006/jmbi.2000.4315

[101] Waterhouse AM, Procter JB, Martin DM, Clamp M, Barton GJ (2009) Jalview Version 2 - a multiple sequence alignment editor and analysis workbench. Bioinformatics 25: 1189–1191. 19151095 10.1093/bioinformatics/btp033 PMC2672624

