# Supplementary figures and images for: The Genome of Spironucleus salmonicida Highlights a Fish Pathogen Adapted to Fluctuating Environments
Source: PLoS Genet. 2014 Feb 6;10(2):e1004053. doi: 10.1371/journal.pgen.1004053 (PMC3916229; doi:10.1371/journal.pgen.1004053)

Frequency

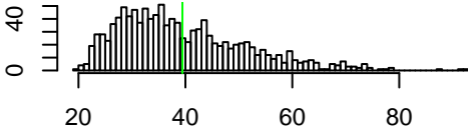

Protein identity

Supplement: Figure S1 — Histogram of the protein identities. Histogram of the protein identities from the 1147 orthologous pairs between S. salmonicida and G. intestinalis. Green line indicates the average protein identity. (PDF) [file pgen.1004053.s001.pdf]

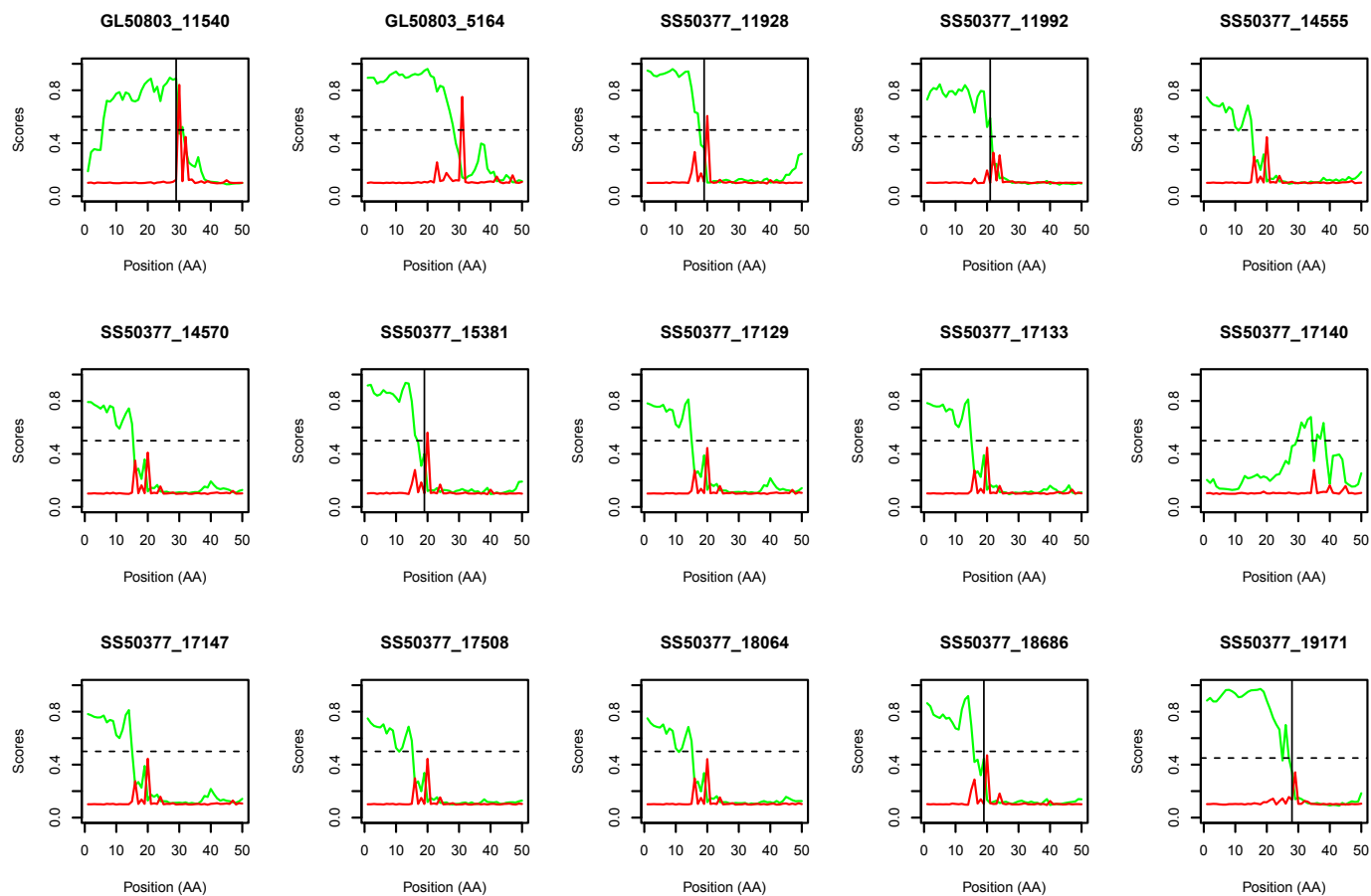

Supplement: Figure S3 — Putative signal peptides characteristics within a group of sugar transporter. SignalP v4.1 [30] was used to analyse signal peptides. C-score (raw cleavage site score) and the S-score (signal peptide score) from the software are shown with red and green lines respectively. Dash lines indicate the score threshold used to claim a positive signal peptide. Black vertical bar indicates the cleavage site if predicted. (PDF) [file pgen.1004053.s003.pdf]

A

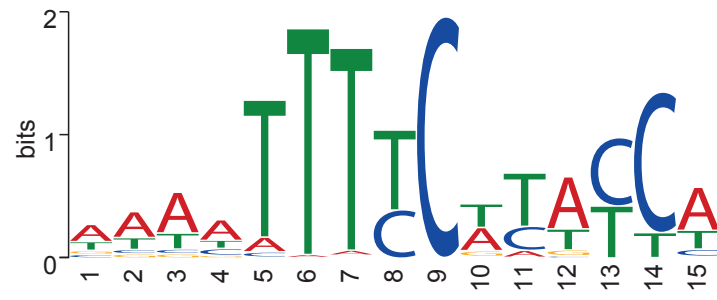

B

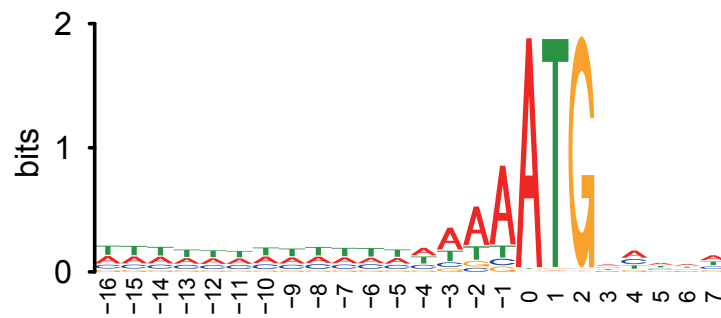

C

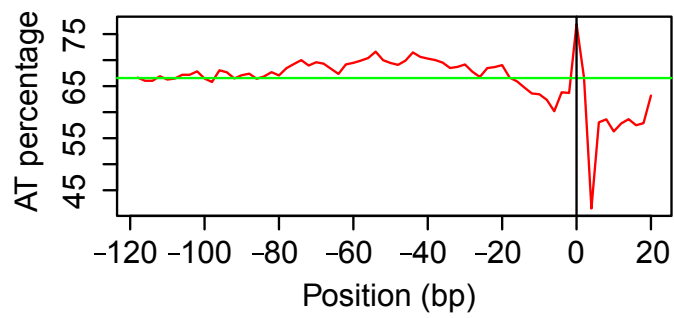

D

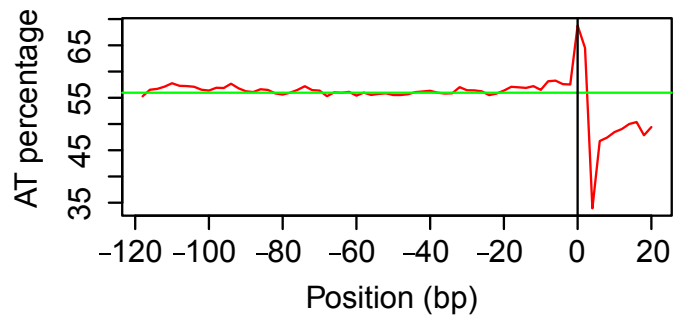

Supplement: Figure S4 — Sequence patterns around diplomonad start codons. A. Sequence logo of C-rich motif found in S. vortens. B. Sequence logo around the S. salmonicida start codon. C and D. AT contents in percentage of the 20 bp C-terminus of all the genes with their 120 bp promoter regions drawn with window size of 3 and step size of 2 for S. vortens and G. intestinalis, respectively. Green line indicates the average AT percentage. (PDF) [file pgen.1004053.s004.pdf]

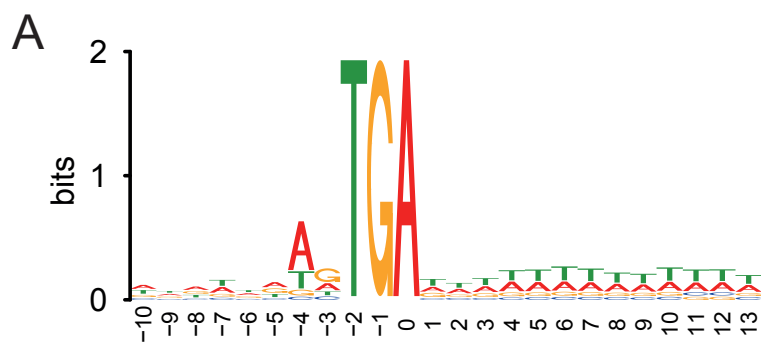

**B**

*S. salmonicida* polyadenylation

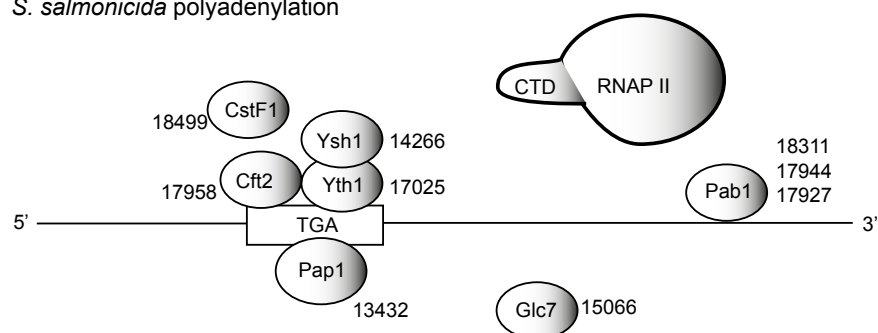

*G. intestinalis* polyadenylation

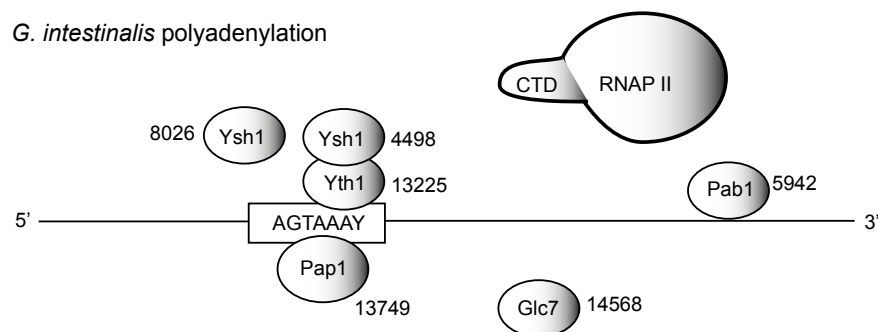

Supplement: Figure S5 — 3′ untranslated regions and polyadenylation machinery. A. Sequence logo around the stop codon. B. Polyadenlylation machinery in S. salmonicida and G. intestinalis. Numbers refer to protein IDs. (PDF) [file pgen.1004053.s005.pdf]

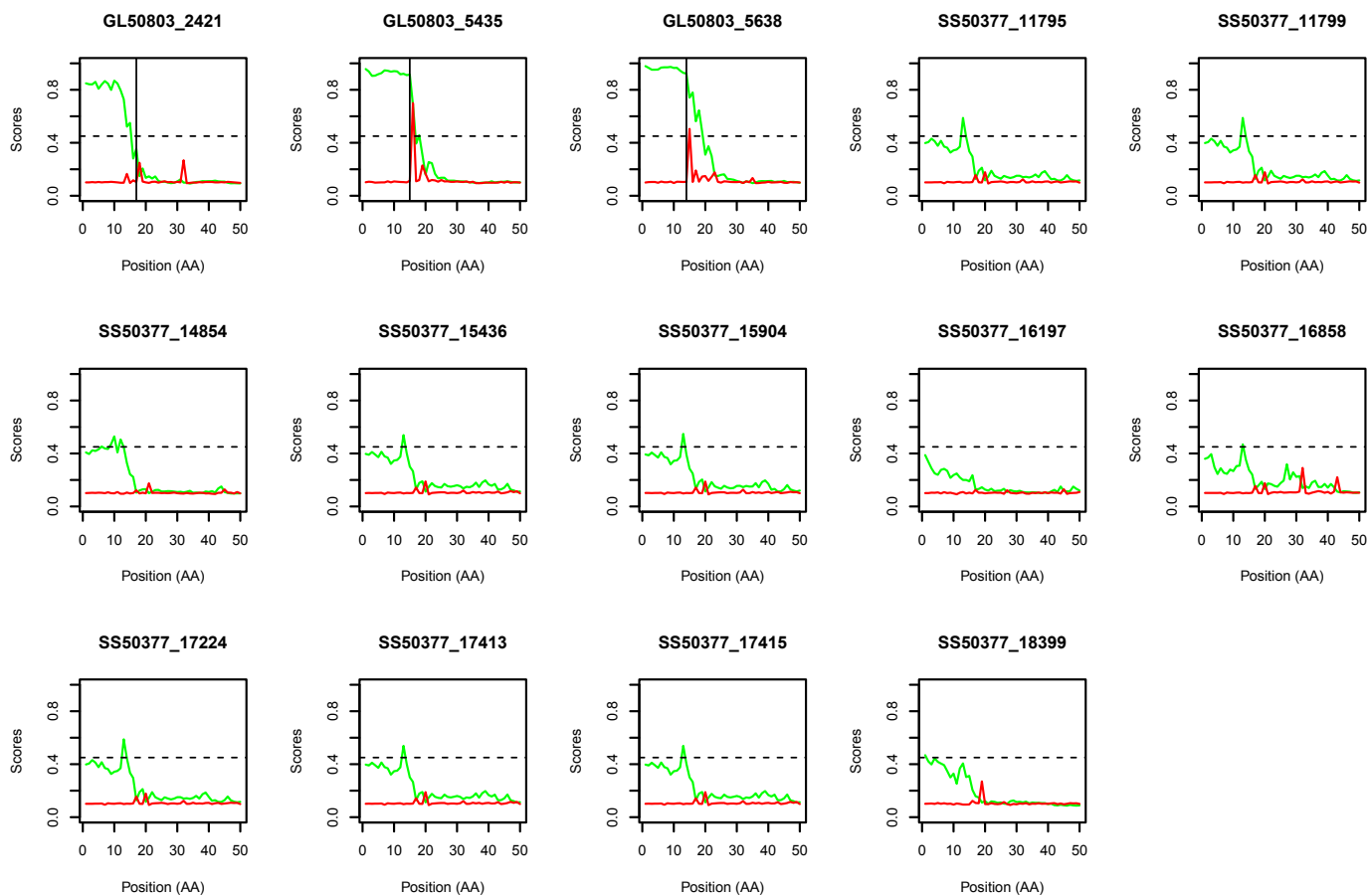

Supplement: Figure S6 — Putative signal peptides characteristics within cyst wall proteins. SignalP v4.1 [30] was used to analyse signal peptides. C-score (raw cleavage site score) and the S-score (signal peptide score) from the software were shown with red and green lines respectively. Dash line indicates the score threshold used to claim a positive signal peptide. Black vertical bar indicates the cleavage site if predicted. (PDF) [file pgen.1004053.s006.pdf]

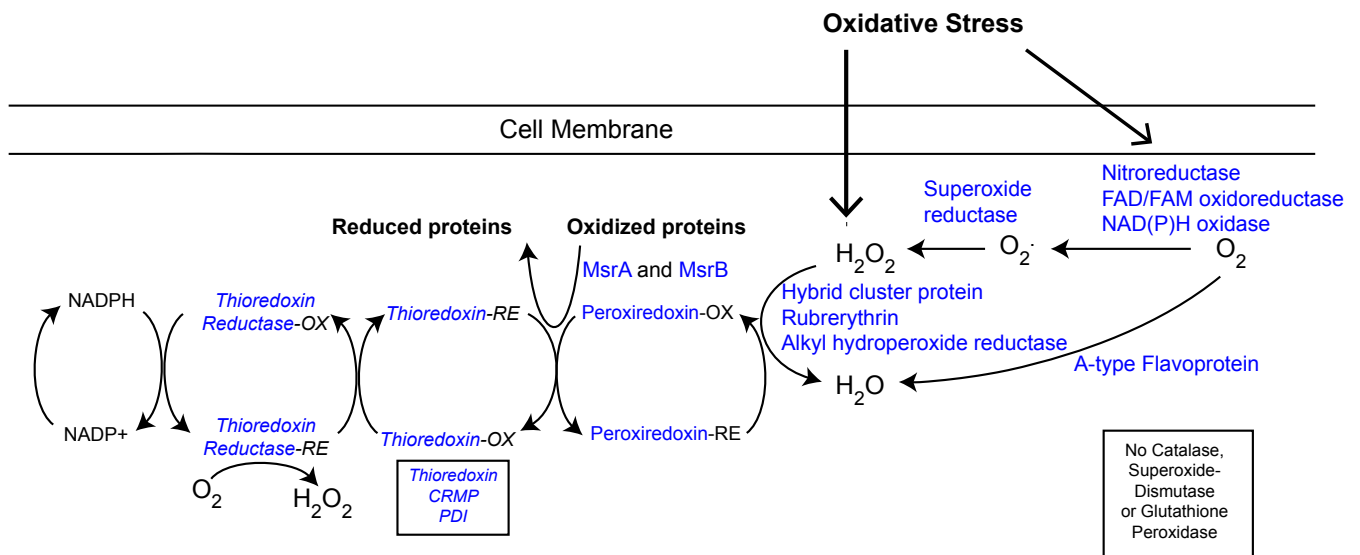

Supplement: Figure S9 — Oxidative stress response in S. salmonicida. Schematic representation of the function of the proteins listed in Table 3. S. salmonicida enzymes are shown in blue. Italic font indicates that putatively proteins performing these functions were identified. (PDF) [file pgen.1004053.s009.pdf]
