# Supplementary material for: The Genome of Spironucleus salmonicida Highlights a Fish Pathogen Adapted to Fluctuating Environments
Source: PLoS Genet. 2014 Feb 6;10(2):e1004053. doi: 10.1371/journal.pgen.1004053 (PMC3916229; doi:10.1371/journal.pgen.1004053)
Supplement: Figure S2 — Alignment of four introns. MUSCLE v3.8.31 [97] was used to align the introns. The AC-repeat motif is underlined. SS50377_17358 marked with * is the gene where the intron failed to be verified using RT-PCR. SS50377_16979 encodes ribosomal protein L30, SS50377_16134 ribosomal protein S24, and SS50377_18398 and SS50377_17358 two hypothetical proteins. (PDF) [file pgen.1004053.s002.pdf]

```
SS50377_16979    GTGAGTTTTAACAACTGAGACCGGTTTGGTCTCAGAAATCTTAACTTTTACTAACAAACTAG
SS50377_16134    GTATGTTTTAA-------------------CAATTAAAAAATAACTTATACTAACAAACTAG
SS50377_18398    GTATGTTTTAA-------------------CTCAATAAATACAACTTTTACTAACAAACTAG
SS50377_17358*   GTATGTCTAAA-------------------CTTTTTTAATGTAACTTATACTAACAAACTAG
                 **. **.* **                   *      *   .***** **************
```
