# Supplementary material for: The Genome of Spironucleus salmonicida Highlights a Fish Pathogen Adapted to Fluctuating Environments
Source: PLoS Genet. 2014 Feb 6;10(2):e1004053. doi: 10.1371/journal.pgen.1004053 (PMC3916229; doi:10.1371/journal.pgen.1004053)
Supplement: Figure S8 — Metabolic reconstruction of G. intestinalis and S. salmonicida. Black arrows indicate enzymatic functions present in both G. intestinalis and S. salmonicida, green arrows indicate functions not detected in G. intestinalis and red arrows indicate functions not detected in S. salmonicida. (PDF) [file pgen.1004053.s008.pdf]

Glucose, pentose-phosphate and selected sugar metabolism

1. glucokinase
2. glucose phosphate isomerase
3. pyrophosphate-dependent phosphofructokinase
4. fructose bisphosphate aldolase
5. triosephosphate isomerase
6. glyceraldehyde 3-phosphate dehydrogenase
7. phosphoglycerate kinase
8. phosphoglyceromutase
9. enolase
10. glycerate kinase
11. phosphomannose isomerase
12. glucosamine 6-phosphate deaminase
13. glucosamine 6-phosphate *N*-acetyltransferase
14. phosphoglucomutase
15. maltose O-acetyltransferase
16. phosphoacetylglucosamine mutase
17. UDP-*N*-acetylglucosamine pyrophosphorylase
18. UDP-*N*- acetylglucosamine 4′ epimerase
19. galactokinase
20. UDP-glucose 4′ epimerase
21. galactose mutarotase
22. β-galactosidase
23. UDP-glucose-1 phosphate uridylyltransferase
24. glucose-6-phosphate 1-epimerase
25. fructokinase
26. glycogen synthase
27. glycogen phosphorylase
28. bifunctional glucose 6-phosphate 1-dehydrogenase / 6-phosphogluconolactonase
29. 6-phosphogluconate dehydrogenase (decarboxylating)
30. transketolase
31. ribose 5-phosphate isomerase
32. ribokinase
33. phosphoribosylpyrophosphate synthetase
34. ribulose phosphate 3-epimerase
35. deoxyribose-phosphate aldolase
36. α-galactosidase
37. ADP-specific glucokinase
38. β-hexosaminidase B (glycosyl hydrolase family 20)
39. α-amylase (glycosyl hydrolase family 13)

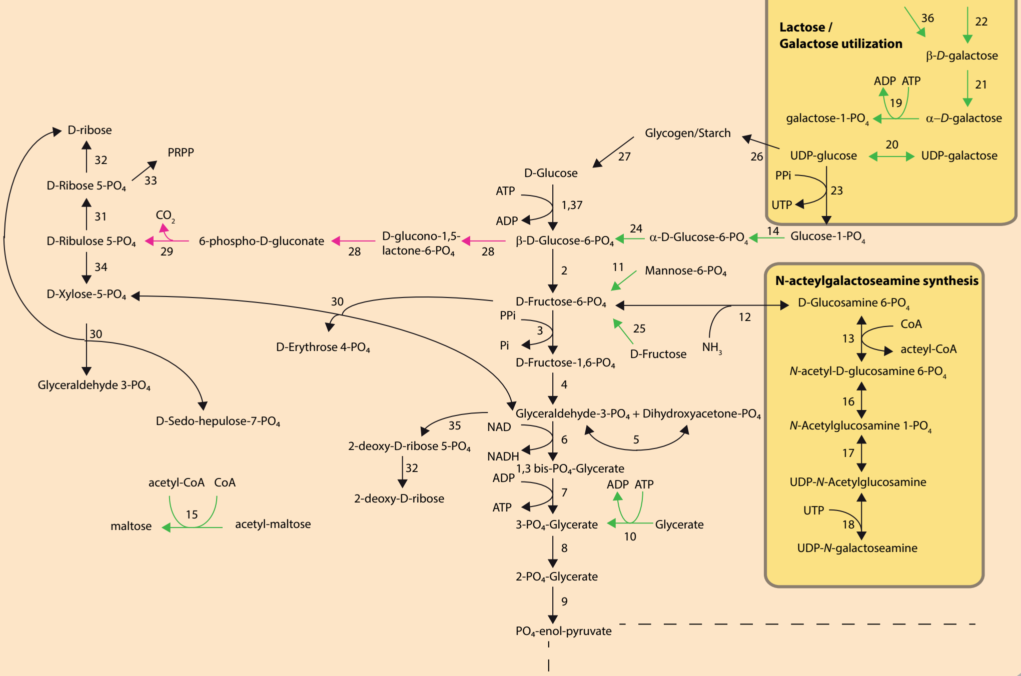

Purine ribonucleoside scavening

1. purine nucleoside phosphorylase
2. inosine-uridine nucleoside N-ribohydrolase
3. adenine phosphoribosyltransferase
4. guanine phosphoribosyltransferase
5. adenylate kinase
6. guanylate kinase

Pyrimidine ribonucleoside scavening

7. uracil phosphoribosyltransferase
8. UMP kinase
9. nucleoside diphosphate kinase
10. CTP synthase
11. cytidine deaminase
12. UMP-CMP kinase
13. uridine/thymine phosphorylase
14. cytosine deaminase
15. cytidine hydrolase

Deoxynucleoside scavening

16. deoxyuridine 5′-triphosphate nucleotidohydrolase
17. deoxyguanosine kinase
18. deoxynucleosidase kinase
19. thymidine kinase
20. thymidylate kinase

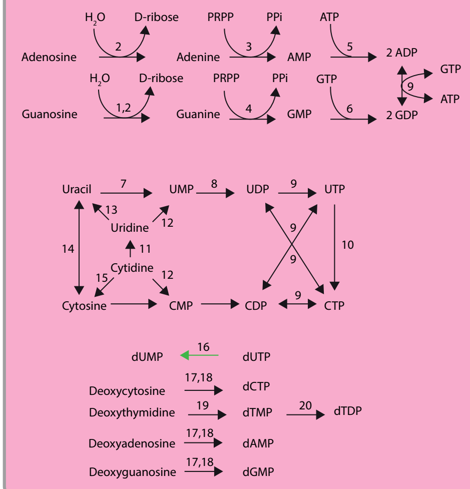

Intermediate pathways to pyruvate synthesis with amino acid fermentation

1. pyruvate kinase
2. alanine transaminase
3. malate dehydrogenase (decarboxylating)
4. malate dehydrogenase
5. aspartate transaminase
6. asparaginase
7. phosphoenolpyruvate carboxyphosphotransferase
8. pyruvate phosphate dikinase
9. asparagine synthase

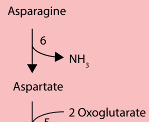

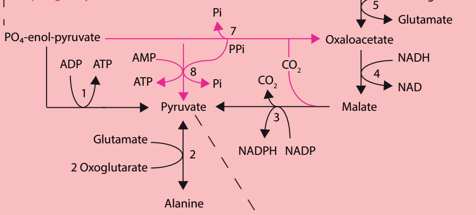

End product synthesis from pyruvate

1. alanine aminotransferase
2. glutamate dehydrogenase
3. pyruvate ferredoxin: oxidoreductase (PFOR)
4. acetyl-CoA synthetase (ADP-forming)
5. alcohol dehydrogase E
6. [FeFe]-hydrogenase

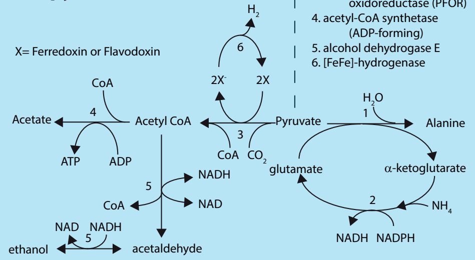

Terpenoid biosynthesis

1. acteyl-CoA acetyltransferase
2. hydoxymethylglutaryl-CoA synthase
3. 3-hydroxy-3-methylglutaryl-CoA reductase
4. mevalonate kinase
5. phosphomevalonate kinase
6. diphoshomevalonate carboxylase
7. isopentenyl-diphosphate delta-isomerase 1
8. farnesyl diphosphate synthase
9. unadecaprenyl pyrophosphate synthase
10. protein farnesyltransferase
11. farnesylated Protein Convering Enzyme 2
12. protein-*S*-isoprenylcysteine *O*-methyltransferase
13. tRNA dimethylallyltransferase

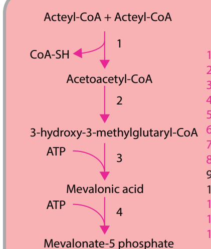

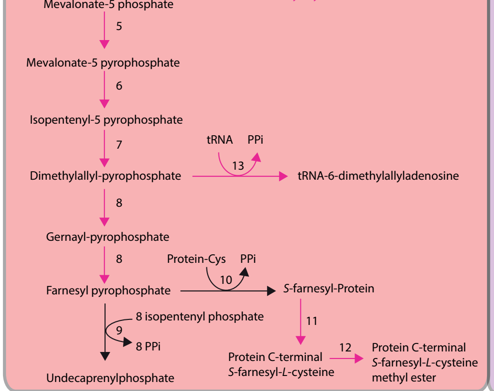

GPI-anchor synthesis

1. phosphomannomutase
2. mannose 1-phosphate guanylyltransferase
3. dolichol-phosphate-mannose synthase
4. GPI-anchor transamidase

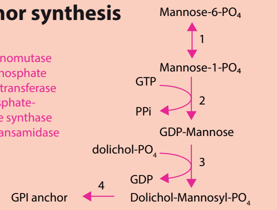

Arginine dihydrolase pathway and proline metabolism

1. arginine deiminase
2. ornithine carbamoyl transferase
3. carbamate kinase
4. ornithine cyclodeaminase
5. pyrroline-5-carboxylate reductase
6. ornithine decarboxylase

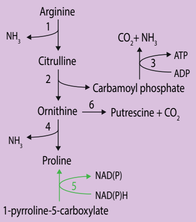

Tryptophan, Serine, Glycine, Cysteine and Methionine metabolism

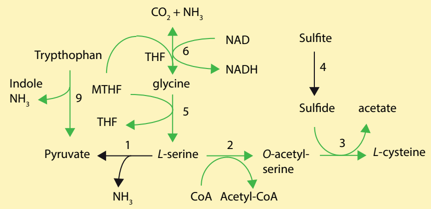

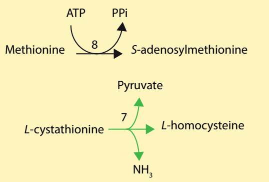

1. *L*-serine dehydratase
2. serine *O*-acetyltransferase
3. cysteine synthase A
4. sulfide dehydrogenase
5. serine hydroxymethyltransferase
6. glycine cleavage system
7. cystathionine β-lyase
8. *S*-adenosylmethionine synthtase
9. tryptophanase

coenzyme A biosynthesis

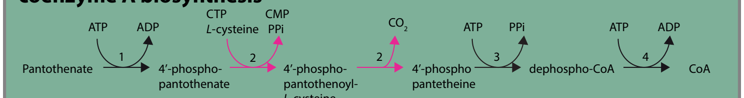

1. pantothenate kinase
2. bifunctional phosphopantothenoyl decarboxylase - phosphopantothenate synthase
3. phosphopantetheine adenylyl transferase
4. dephospho-CoA kinase
