# Supplementary material for: The Genome of Spironucleus salmonicida Highlights a Fish Pathogen Adapted to Fluctuating Environments
Source: PLoS Genet. 2014 Feb 6;10(2):e1004053. doi: 10.1371/journal.pgen.1004053 (PMC3916229; doi:10.1371/journal.pgen.1004053)
Supplement: Protocol S1 — A detailed description of the sequencing data, genome assembly and annotation. (PDF) [file pgen.1004053.s010.pdf]

Protocol S1

The genome of *Spironucleus salmonicida* highlights a fish pathogen adapted to fluctuating environments

Feifei Xu, Jon Jerlström-Hultqvist, Elin Einarsson, Ásgeir Ástvaldsson, Staffan G. Svärd, Jan O. Andersson

Sequencing Data

Four runs of sequenceing data were generated for the *Spironucleus salmonicida* genome project. The details about the data and its corresponding library information and sequencing technology are listed in Table 1.

Table 1: Summary of all the sequencing data in the genome project.

	DNA		RNA	
Instrument Model	454 GS FLX XLR Titanium		Illumina Genome Analyzer IIx	
Library Layout	Single read	Mate pairs, 3 kb insert	Paired-end, 350 bp insert	Paired-end, 175 bp insert
Read count	1,336,623	788,763	18,886,541×2	19,567,992×2
Base count (Mb)	408.5	254.3	3777.3	3913.6
Sequencing Depth	34	21	301	290
Mean read length (bp)	305	322	100	100

We did not do a contamination check at reads level as it introduces a risk of taking out true sequences with biologically interesting features, for example genes horizontally transferred from bacteria. However the subsequent annotation work showed no sign of contamination at the assembly level.

Genome assemblies

As mentioned in the main text, only 454 data was used in constructing *de novo* genome assembly. We tested three different *de novo* genome assemblers - Newbler, Mira [1] and Celera Assembler [2] - in an attempt to find a good assembly from the sequenced data.

Newbler v2.3, provided by the instrument vendor (Roche 454 Life Sciences), was used with default settings. Celera Assembler (CA) v6.0 [2] was run with the default error rate and also the suggested settings for 454 data. Both assemblers produces scaffolds as well as contigs using available mate-pair reads. Mira V3rc4 [1] was run with "-job=denovo,genome,normal,454" settings. It produces only contigs and needs subsequent scaffolding programs, like Consed [3] or Bambus [4], to order contigs. Bambus gave poor

scaffolding results, while Consed did a reasonable job in scaffolding but did not estimate gap sizes, and those results are not shown here.

Basic statistics of three different assemblies from the three different assemblers are shown in Table 2. Celera Assembler uses mate-pair information extensively, even at the contig-building stage, which likely explains why it has the smallest number and largest contigs. Furthermore, the CA assembly has the genome size closest to the estimation from optical maps (12.6 Mbp), and the smallest number of scaffolds. It was difficult to judge the quality of assemblies based on those values, so we further compared the contents of the assemblies, using MUMmer [5].

Table 2: Comparisons in the statistics of three different assemblies

	**Newbler**	**MIRA**	**CA**
Size (Mbp)	12.0	13.8	12.9
Avg. genome coverage	43.0	42.5	40.0
No. of reads used	2,442,701	2,497,406	2,390,565
GC content (%)	32.1	33.9	30.4
No. of paired reads used	439,772	-	468,994
No. of scaffolds	261	-	232
N50 (scaffolds) (kbp)	156.5	-	151.4
Largest scaffold (kbp)	558.2	-	561.3
Avg. scaffold length (kbp)	45.9	-	55.5
No. of contigs	1482	1466	452
Largest contig (kbp)	255.4	174.7	421.7
Avg. contig length (kbp)	7.9	5.1	28.5
N50 (contigs) (kbp)	30.4	32.4	80.2

MUMmer v3.07 [5] was used to align assemblies and generate comparison dotplots. Comparisons between each pair assemblies show that all the three assemblies contain the same genome content, and the size differences are caused by different copies of short repeatitive regions (Figures 1a-d). The comparisons of the scaffolds between the assemblies from Newbler and CA disagree in a few small regions (Figure 1c). Without a reference genome, it was difficult to judge which is mis-assembled, and possibly neither is completely correct.

However, the assemblies are similar enough that whichever assembly we use, it is unlikely that we miss much important genomic content. The mis-assemblies caused by repeats are unlikely to be resolved without sequencing mate-pair libraries with larger inserts.

We thus chose the assembly from CA assembler with the minimum number of scaffolds and good estimated genome size for further analysis. It is beneficial especially for annotation to have large scaffolds instead of many small contigs. And it is easier to break up scaffolds if mis-assembly is found later, than to scaffold by hand afterwards. So the CA assembly in Table 2 is chosen for later process.

The optical maps independent of genomic information also supported the CA assembly we picked. The assembly was one of the best assemblies mapped on the optical maps. MapSolver provided by OpGen was used to align between the assembly and optical maps. 74 out of 232 scaffolds are aligned to the optical

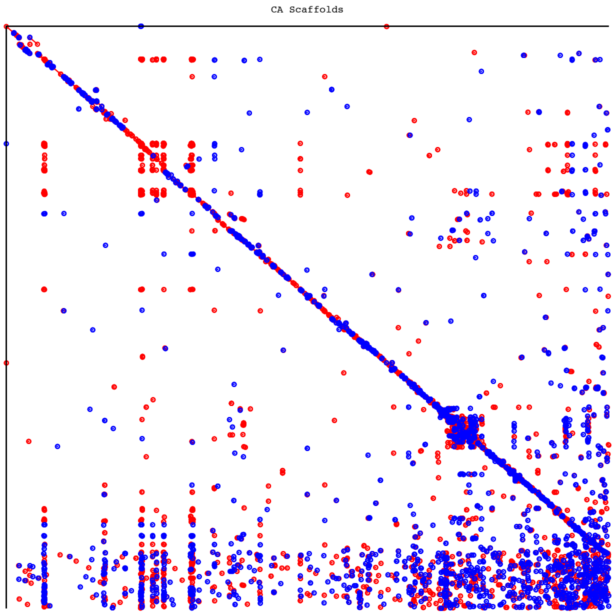

(a) CA scaffolds against MIRA contigs.

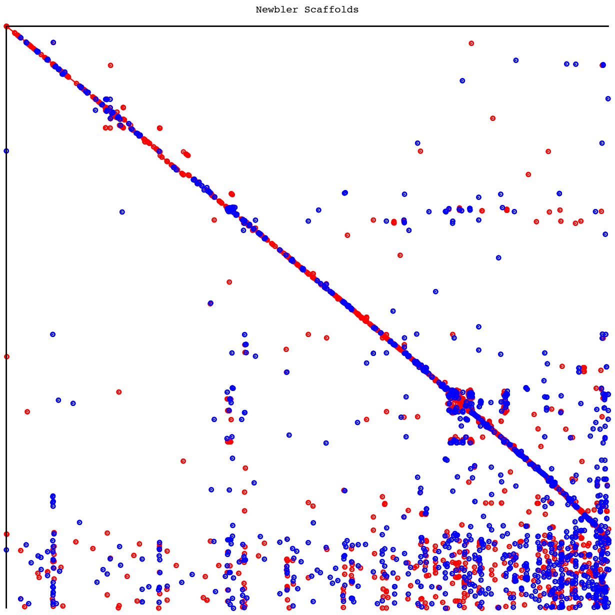

(b) Newbler scaffolds against MIRA contigs.

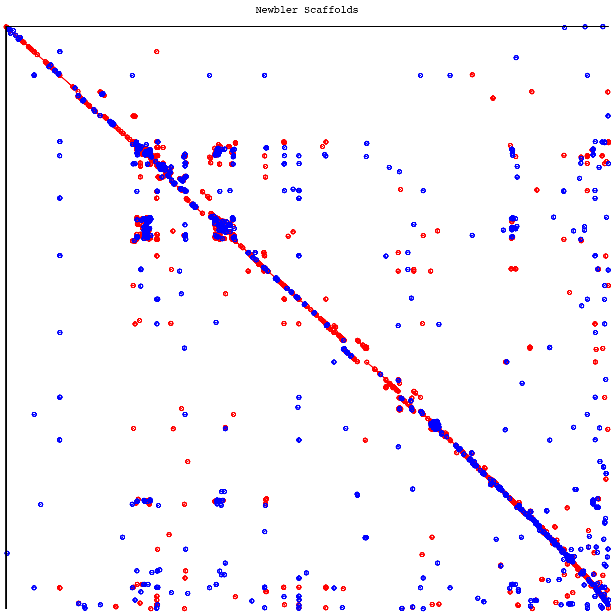

(c) Newbler scaffolds against CA scaffolds.

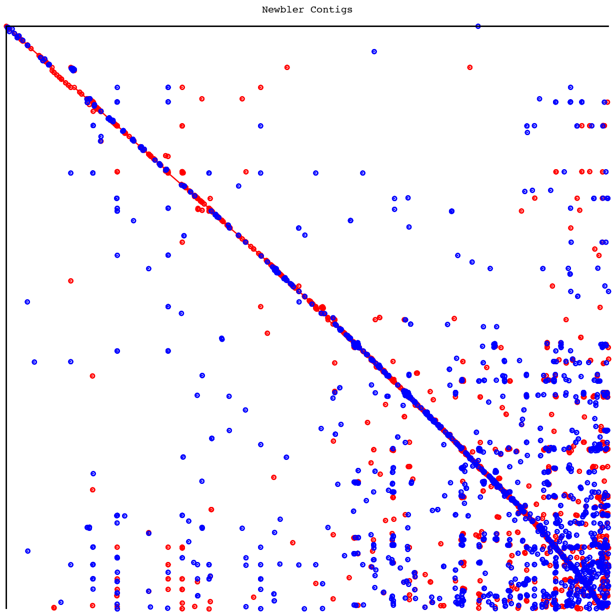

(d) Newbler contigs against CA contigs.

Figure 1: Dot-plot comparisons between different scaffolds/contigs generated by MUMmer. Blue colors forward matches while red means reverse matches.

maps, resulting in 9.6 Mb of assembly placed and 64.8% of the genome covered. There are 74 gaps in total with 10.4% of them in closable distance (5 kb) by PCR.

Correcting the assembly using Illumina data

454 data is known for its homopolymer errors, which stem from the fact that runs of the same nucleotide are intrinsically difficult for pyrosequencing methods. The Illumina data was generated using a different technology which does not have this problem. Therefore, it should be possible to corrects homopolymer errors using these data. To make the best use of the Ilumina data, it would be best if we could make a hybrid assembly. In that way, it would correct the 454 sequencing errors while at the same contributing to scaffolding. CA v6.1 and Newbler v2.6 claim to generate hybrid assembly. We only tested CA v6.1 using all the 454 reads and 100X Illumina reads. It failed at overlapping stage because of memory constraints. Due to facility and time issue, we instead used Illumina data only to correct the 454 assembly.

BWA v0.5.9 [6], was used to map Illumina DNA reads against the chosen CA assembly. 92.1% of reads were mapped with 89.7% properly paired. Nesoni v0.40 [7] was used to correct the consensus based on the mapping results. There were 2977 positions updated including 131 deletions, 1388 insertions and 1458 substitutions. This updated assembly was then used for annotation.

Annotation

The standard approach for annotations is to use information from related organisms that have previously been sequenced and analyzed. There are currently three diplomonad genomes published, all isolates from the intestinal parasite *G. intestinalis* [8–10]. Here we use the *G. intestinalis* isolate WB genome for comparison, since it is most complete and served as reference for the other two *Giardia* genomes.

MUMmer [5] was used for comparison of two genomes. There was no similarity detected on nucleotide level, and limited similarity on the protein level, shown in the dot plot comparison (Figure 2). Only 525 kb (4%) of the *S. salmonicida* genome has similarity to *G. intestinalis* WB genome on amino acid level, and the average similarity of the matches is around 70%. These values are calculated based on the alignment information generated by MUMmer. This makes it impossible to copy annotations from *G. intestinalis*. We have to carefully annotate the *S. salmonicida* genome from scratch. On the positive side, this reduces the risk of propagating bad annotations.

We have done the annotation in two stages. We first generated an annotation from a computation pipeline, then reviewed the annotation manually combining information from different sources (detailed see below).

Computational pipeline

The computation annotation pipeline combines the information from gene prediction programs, domain hits, expressed sequence tags (ESTs) and RNA-Seq data, and performs gene calling. When the structural annotation is decided, functional annotation is assigned with BLASTP [11] and motif hits.

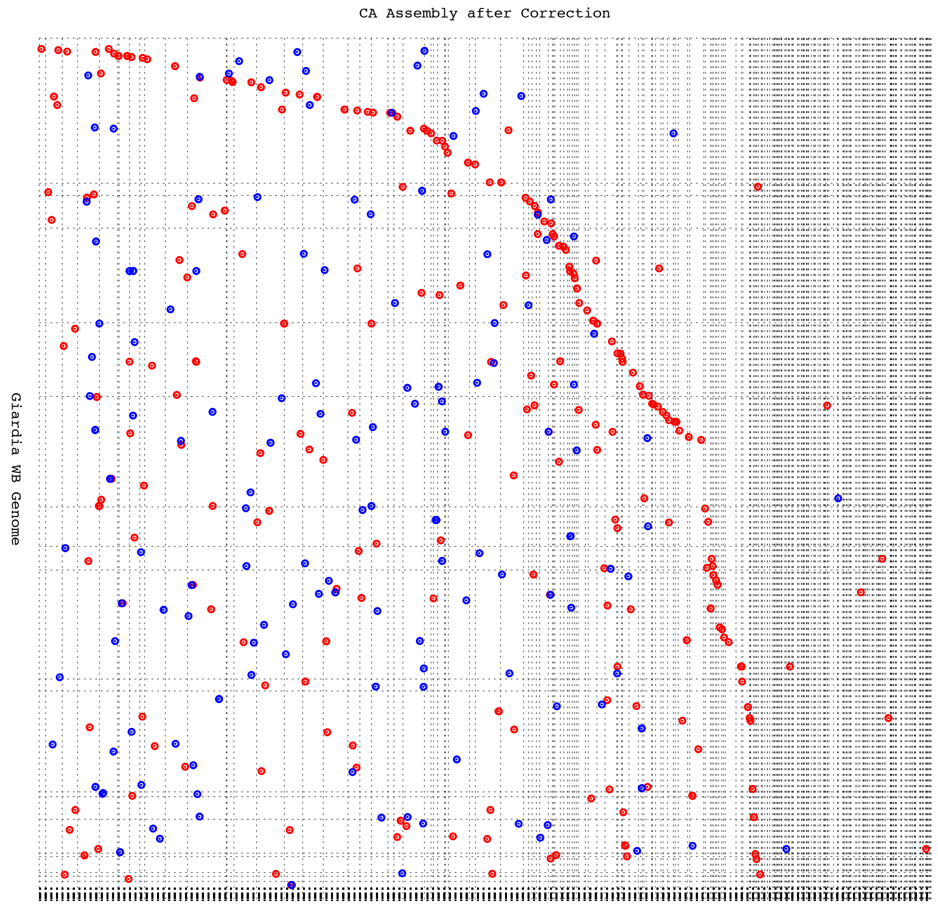

Figure 2: Dot-plot comparison of CA assembly after correction by Illumina data against *G. intestinalis* WB genome on protein level, generated by MUMmer. Only matches with more than 50% similarity is displayed. Blue colors forward matches while red means reverse matches. The sizes of the scaffolds are represented by the grid.

Structural annotation - evaluating gene finding softwares

The first challenge is to find gene prediction programs which work with our genome and have good performance. *S. salmonicida* uses alternative genetic code 6, where UAA and UAG encode glutamine instead of being stop codons [12]. A lot of widely-used gene predictions programs for eukaryotes, like GeneMark and SNAP, do not support alternative genetic codes. GlimmerHMM [13] and AUGUSTUS [14] are the ones we found to support alternative codes, however only GlimmerHMM was used in our pipeline due to the complexity of AUGUSTUS. Diplomonad genomes seem to be very intron-poor. Six introns have been found in *G. intestinalis* [10], and the sequence survey of the *S. salmonicida* genome failed to indicate any introns [15]. Therefore, we reasoned that prokaryotic gene prediction programs also could work well for the genome. Prodigal v2.50 [16] and Glimmer v3.02 [17] were thus used.

A set of 517 highly reliable genes was used to train gene prediction programs. The genes have confident orthologs within the *G. intestinalis* genomes and were closely inspected manually with the help of RNA-Seq data. GlimmerHMM v3.0.1 was trained with "-n 150 -v 50", which limits the intergenic region

to be 150 bp (default 250 bp) and the flanking region around a gene to be 100 bp (default 200 bp). The configuration file for training was also modified, setting "BoostSgl 10" to increase the predicted number of single exon genes and specifying the stop codon to be only "TGA". GlimmerHMM predicted 9723 genes using these settings.

Glimmer3 was run with mostly default settings, except altering the genetic code table to be 6 and maximum overlap length between genes to be 10, which predicted 6042 genes.

Prodigal has its own built-in training mechanism using long ORFs, and was run with default settings except genetic code table 6 was specified. It predicted 11,746 genes.

RNA-Seq data was used to judge the performance of the different gene prediction programs. They were all loaded into Artemis [18] for the comparison. RNA-seq data was mapped on the genome using BWA [6] and visualized in Artemis with BAMview [19]. By inspecting the consistence between the genes predicted and the boudaries of RNA-Seq reads mapped, we concluded that GlimmerHMM worked best for the *S. salmonicida* genome. Prodigal performed second best, and we later found during manual annotation that it was often compensating GlimmerHMM in some problematic regions. Glimmer3 prediction did not correspond well to the RNA-Seq data. The performance of these tests were used to set different weights for gene calling later in the pipeline (Table 3).

Structural annotation - additional evidence used to evaluate gene calls

The gene predictions from the three programs were evaluated using additional sources of information: ESTs, RNA-Seq and sequence similarity to protein domains. EVidenceModeler (EVM) [20], was used to combine the different types of evidence.

There are EST data available from NCBI both from *S. salmonicida* itself and other members of the *Spironucleus* genus: *Spironucleus vortens* and *Spironucleus barkhanus*. The BLAST programs [11] were used to perform similarity searches between ESTs and the genome. BLASTN was used for *S. salmonicida* EST data. TBLASTX was used for EST data from *S. vortens* and *S. barkuhanus*. The E-value cutoff 1e-05 was used in all searches.

The RNA-Seq data was mapped to the genome by BWA and then the mapped positions were extracted by Cufflinks v1.0.3 [21]. It was weighted heavily in the annotation pipeline due to its high quality.

Domain hits were also weighted heavily in the pipeline. Pfam [22] and TIGRFAM [23] domain databases were both used in domain searches using HMMER 3.0 [24]. Pfam 25.0 contains 12,273 families, and TIGRFAM 10.0 contains 4023 families in March 2011. Domains hits with score $\geq$20 are regarded as good hits, while domain hits with scores between 20 and 15 are considered as weak hits. They were weighted differently (Table 3). Using the weights listed in Table 3, EVM predicted 9244 genes which were used for functional annotation.

There were several things we noticed while testing EVM. Gene predictions are important. The consensus prediction is based on the pool of genes predicted. So if the gene predictions are poor to begin with, EVM is not able to make new good predictions based on other strong evidence. If there are several different gene prediction programs supporting the same gene calling, it is likely that the gene is called in the consensus as

Table 3: Weights used for different evidence in EVM

Evidence	Weight
Prodigal gene prediction	4
GlimmerHMM gene prediction	5
Glimmer3 gene prediction	1
HMMER3 good	7
HMMER3 weak	3
RNA-Seq	20
S. salmonicida BLASTN	5
S. vortens TBLASTX	5
S. barkhanus TBLASTX	5

well. EVM is designed for eukaryotic genomes, and does not allow gene overlapping. Putative gene overlaps have been found in the *S. salmonicida* genome [15], these have to be adjusted during manual annotation. Luckily, overlapping genes are easy to spot with RNA-seq data and seem to be rare.

To conclude, with the combination of multiple gene calling software and other sources of information we believe we have a pipeline that is able to produce a reasonable structural annotation to be used for functional annotation.

Functional annotation

The pipeline for functional annotation was designed as a hierarchy of similarity searches. *G. intestinalis* is the closest species that has been published. The annotation is assigned first by good similarity searches (e-value $\leq$1e-10) against *G. intestinalis* database, then the NCBI non-redundant protein database. If the best BLAST hit is of intermediate quality (1e-10$<$ e-value $\leq$1e-05), domain hits are also indicated in the annotation. Functions based on domain hits, if any, are assigned to genes lacking BLAST hits. When there are no BLAST nor domain hits, the gene is automatically assigned as a “hypothetical protein”. GFF3 format is used for storing annotation information.

Manual annotation

Annotations generated by any automatic pipeline need to be reviewed by scientists. An important aspect of this process is to make it as user-friendly as possible because it is repetitive and time-consuming. We carry out manual annotation in Artemis, a genome browser as well as an annotation editor [18].

Along with the genome consensus FASTA file and annotation GFF3 file, all the relevant information are also available to load into the Artemis window. RNA-Seq data mapped by BWA can be loaded into Artemis with BAMView. All the other information are prepared in GFF3 format, including the three gene predictions, domain searches against two domain databases, three EST similarity searches, transmembrane predictions, signal peptide predictions, tRNAs predictions, other RNA families predictions, and repeats.

Transmembrane domains were predicted using TMHMM v2.0 [25] with default settings. 1442 proteins were predicted with transmembrane helices and 524 of them contained more than one.

SignalP v4.0 [26] online service was used to predict signal peptides. With the default settings, there were 119 genes predicted containing signal peptides.

tRNAs were called by tRNAScan-SE v1.23 [27]. The most sensitive co-variance model, using Cove analysis only, was used by specifying "-C". It found 145 tRNAs in total, 143 tRNAs coding for all 20 standard amino acids, 1 selenocysteine tRNAs as well as one coding tRNA with unknown isotypes. 10 of the tRNAs predicted contained introns.

Other RNA families are predicted by similarity search against Rfam database v10.0 containing 1446 families [28] using infernal v1.0.2 [29]. 1815 results were found with the suggested cutoff (e-value $\leq$0.1), including 146 tRNAs and 7 rRNAs.

RepeatMasker v3.3.0 [30] with library version rm-20110920 was used to detect the repetitive region of the genome. "-frag 600000 -excln -gccalc" settings were used. "-frag 600000" was specified to force the program not to fragmentize the scaffolds; "-excln" was given to calculate the percentages displayed in the statistics file using the total sequence length excluding runs of 25 Ns or more. It was necessary because the draft genome consists of concatenated contigs separated by long stretches of Ns. "-gccalc" option forced RepeatMasker to use the average GC level of the genome. It masks 5.2% of the genome, including 4 MIRs (SINEs), 31 LINEs, 2 LTR, 3 DNA elements, 130 small RNA, 424 Simple repeats and 4.8% of genome as low complexity.

During the manual annotation, we also used a "confidence" flag to keep track the reliability of an annotation. There are four levels of confidence. Confidence 4 means both confident functional and structural annotation; confidence 3 indicates either good structural or functional annotation; confidence 2 suggests reasonable structural and/or functional annotation; confidence 1 implies weak structural annotation without functional annotation.

After manually inspecting all the genes, we arrived at 9275 genes, including 1895 highly confident genes, 3275 genes with good confidence, 2650 with modest confidence and 1455 genes of low confidence. The genes with low confidence tends to be smaller in size and lower in GC contents (Figure 3). We decided then to filter out genes with low confidence and are shorter than 150 bp. This removed 921 genes and resulted in 8354 genes in the current annotation, including 267 partial genes and 21 genes with in-frame stop codon caused by frameshift.

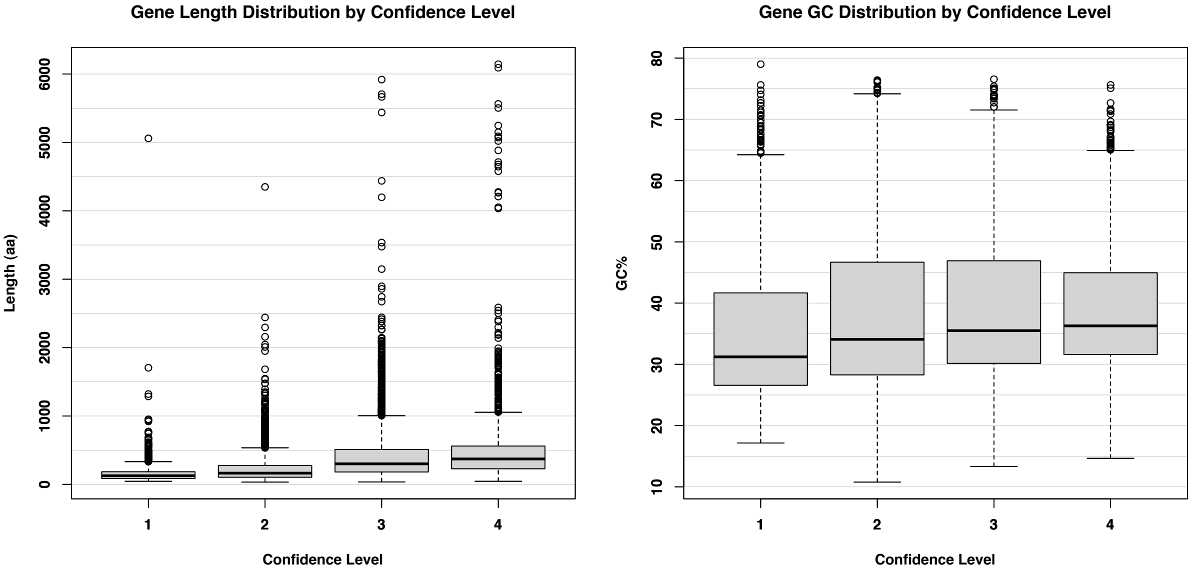

(a) Gene Length Distribution

(b) Gene GC% Distribution

Figure 3: Gene length and GC% distribution at different confidences. **a.** is the gene length distribution grouped by confidence levels. **b.** is the GC content percentage distribution of the genes in different confidences. The distribution is presented as box plot with the mean value in each group marked as a black line across the box.

References

[1] Chevreux B, Wetter T, Suhai S: **Genome Sequence Assembly Using Trace Signals and Additional Sequence Information**. *Computer Science and Biology: Proceedings of the German Conference on Bioinformatics (GCB)* 1999, **99**:45–56.

[2] Miller JR, Delcher AL, Koren S, Venter E, Walenz BP, Brownley A, Johnson J, Li K, Mobarry C, Sutton G: **Aggressive assembly of pyrosequencing reads with mates.** *Bioinformatics* 2008, **24**(24):2818–2824.

[3] Gordon D, Abajian C, Green P: **Consed: a graphical tool for sequence finishing.** *Genome Research* 1998, **8**(3):195–202.

[4] Pop M, Kosack DS, Salzberg SL: **Hierarchical scaffolding with Bambus.** *Genome Research* 2004, **14**:149–159.

[5] Kurtz S, Phillippy A, Delcher AL, Smoot M, Shumway M, Antonescu C, Salzberg SL: **Versatile and open software for comparing large genomes.** *Genome Biology* 2004, **5**(2):R12.

[6] Li H, Durbin R: **Fast and accurate short read alignment with Burrows-Wheeler transform.** *Bioinformatics* 2009, **25**(14):1754–1760.

[7] Harrison P, Seemann T:
Nesoni [http://www.vicbioinformatics.com/nesoni.shtml].

[8] Morrison HG, McArthur AG, Gillin FD, Aley SB, Adam RD, Olsen GJ, Best AA, Cande WZ, Chen F, Cipriano MJ, Davids BJ, Dawson SC, Elmendorf HG, Hehl AB, Holder ME, Huse SM, Kim UU, Lasek-Nesselquist E, Manning G, Nigam A, Nixon JEJ, Palm D, Passamaneck NE, Prabhu A, Reich CI, Reiner DS, Samuelson J, Svärd SG, Sogin ML: **Genomic minimalism in the early diverging intestinal parasite Giardia lamblia.** *Science* 2007, **317**(5846):1921–1926.

[9] Franzén O, Jerlström-Hultqvist J, Castro E, Sherwood E, Ankarklev J, Reiner DS, Palm D, Andersson JO, Andersson B, Svärd SG: **Draft genome sequencing of giardia intestinalis assemblage B isolate GS: is human giardiasis caused by two different species?** *PLoS Pathogens* 2009, **5**(8):e1000560.

[10] Jerlström-Hultqvist J, Franzén O, Ankarklev J, Xu F, Nohynkova E, Andersson JO, Svärd SG, Andersson B: **Genome analysis and comparative genomics of a Giardia intestinalis assemblage E isolate.** *BMC Genomics* 2010, **11**:543.

[11] Altschul SF, Gish W, Miller W, Myers EW, Lipman DJ: **Basic local alignment search tool.** *Journal of Molecular Biology* 1990, **215**:403–410.

[12] Keeling PJ, Doolittle WF: **Widespread and ancient distribution of a noncanonical genetic code in diplomonads.** *Molecular Biology and Evolution* 1997, **14**(9):895–901.

[13] Majoros WH, Pertea M, Salzberg SL: **TigrScan and GlimmerHMM: two open source ab initio eukaryotic gene-finders.** *Bioinformatics* 2004, **20**(16):2878–2879.

[14] Stanke M, Schöffmann O, Morgenstern B, Waack S: **Gene prediction in eukaryotes with a generalized hidden Markov model that uses hints from external sources.** *BMC Bioinformatics* 2006, **7**:62.

[15] Andersson JO, Sjögren AM, Horner DS, Murphy CA, Dyal PL, Svärd SG, Logsdon JM, Ragan MA, Hirt RP, Roger AJ: **A genomic survey of the fish parasite Spironucleus salmonicida indicates genomic plasticity among diplomonads and significant lateral gene transfer in eukaryote genome evolution.** *BMC Genomics* 2007, **8**:51.

[16] Hyatt D, Chen GL, Locascio PF, Land ML, Larimer FW, Hauser LJ: **Prodigal: prokaryotic gene recognition and translation initiation site identification.** *BMC Bioinformatics* 2010, **11**:119.

[17] Delcher AL, Bratke KA, Powers EC, Salzberg SL: **Identifying bacterial genes and endosymbiont DNA with Glimmer.** *Bioinformatics* 2007, **23**(6):673–679.

[18] Rutherford K, Parkhill J, Crook J, Horsnell T, Rice P, Rajandream MA, Barrell B: **Artemis: sequence visualization and annotation.** *Bioinformatics* 2000, **16**(10):944–945.

[19] Carver T, Böhme U, Otto TD, Parkhill J, Berriman M: **BamView: viewing mapped read alignment data in the context of the reference sequence.** *Bioinformatics* 2010, **26**(5):676–677.

[20] Haas BJ, Salzberg SL, Zhu W, Pertea M, Allen JE, Orvis J, White O, Buell CR, Wortman JR: **Automated eukaryotic gene structure annotation using EVidenceModeler and the Program to Assemble Spliced Alignments.** *Genome Biology* 2008, **9**:R7.

[21] Trapnell C, Williams BA, Pertea G, Mortazavi A, Kwan G, van Baren MJ, Salzberg SL, Wold BJ, Pachter L: **Transcript assembly and quantification by RNA-Seq reveals unannotated transcripts and isoform switching during cell differentiation.** *Nature Biotechnology* 2010, **28**(5):511–515.

[22] Finn RD, Mistry J, Tate J, Coggill P, Heger A, Pollington JE, Gavin OL, Gunasekaran P, Ceric G, Forslund K, Holm L, Sonnhammer ELL, Eddy SR, Bateman A: **The Pfam protein families database.** *Nucleic Acids Research* 2010, **38**:D211–22.

[23] Selengut JD, Haft DH, Davidsen T, Ganapathy A, Gwinn-Giglio M, Nelson WC, Richter AR, White O: **TIGRFAMs and Genome Properties: tools for the assignment of molecular function and biological process in prokaryotic genomes.** *Nucleic Acids Research* 2007, **35**:D260–4.

[24] **HMMER 3.0** [http://hmmer.janelia.org].

[25] Krogh A, Larsson B, von Heijne G, Sonnhammer EL: **Predicting transmembrane protein topology with a hidden Markov model: application to complete genomes.** *Journal of Molecular Biology* 2001, **305**(3):567–580.

[26] Petersen TN, Brunak S, von Heijne G, Nielsen H: **SignalP 4.0: discriminating signal peptides from transmembrane regions.** *Nature Methods* 2011, **8**(10):785–786.

[27] Lowe T: **tRNAscan-SE: a program for improved detection of transfer RNA genes in genomic sequence**. *Nucleic Acids Research* 1997, **25**(5):955–964.

[28] Gardner PP, Daub J, Tate J, Moore BL, Osuch IH, Griffiths-Jones S, Finn RD, Nawrocki EP, Kolbe DL, Eddy SR, Bateman A: **Rfam: Wikipedia, clans and the "decimal" release.** *Nucleic Acids Research* 2011, **39**:D141–5.

[29] Nawrocki EP, Kolbe DL, Eddy SR: **Infernal 1.0: inference of RNA alignments.** *Bioinformatics* 2009, **25**(10):1335–1337.

[30] Smit A, Hubley R, Green P: **RepeatMasker Open-3.0.** 1996-2011, [http://www.repeatmasker.org].
