# Supplementary material for: The Genome of Spironucleus salmonicida Highlights a Fish Pathogen Adapted to Fluctuating Environments
Source: PLoS Genet. 2014 Feb 6;10(2):e1004053. doi: 10.1371/journal.pgen.1004053 (PMC3916229; doi:10.1371/journal.pgen.1004053)
Supplement: Table S1 — S. salmonicida proteins without homologs in G. intestinalis. (PDF) [file pgen.1004053.s011.pdf]

Table S1 ***S. salmonicida*** **proteins without homologs in** ***G. intestinalis***

Count	Gene annotation
3867	Hypothetical protein
240	Transmembrane domain-containing protein
156	Conserved hypothetical protein
64	Myb-like DNA-binding domain-containing protein
43	Cysteine-rich membrane protein 2
40	Cysteine-rich membrane protein 1
25	Cysteine-rich protein
10	Palmitoyl-protein thioesterase
6	E2F/DP family winged-helix DNA-binding domain-containing protein
6	Major facilitator superfamily protein
5	Zinc finger, C2H2 type domain-containing protein
4	Carotenoid isomerase
4	Frataxin-like protein
4	RNA recognition motif-containing protein
3	Aldose 1-epimerase
3	Beta-galactosidase
3	Cysteine synthase A
3	Glycosyl hydrolase family 20 protein
3	Hemolysin III family protein
3	LSM domain protein
3	Lipase class 3 family protein
3	Plasma-membrane choline transporter and transmembrane domain-containing protein
3	Rubrerythrin 1
3	TBC domain-containing protein
3	Tryptophanase
3	Ubiquitin carboxyl-terminal hydrolase family protein
3	Zinc finger domain-containing protein
2	5' nucleotidase family protein
2	Bifunctional phosphopantothenoylcysteine decarboxylase - phosphopantothenate synthase
2	Bromodomain-containing protein
2	CLN3 protein
2	Calponin homology (CH) domain-containing protein
2	Cofilin/tropomyosin-type actin-binding protein
2	EAP30/Vps36 domain-containing protein
2	Galactokinase
2	Glucokinase, ROK family
2	Glycerophosphoryl diester phosphodiesterase family protein
2	HMG (high mobility group) box domain-containing protein
2	Hsp20/alpha crystallin family protein
2	M18 family aminopeptidase
2	Macro domain-containing protein
2	Maltose O-acetyltransferase
2	Microsomal signal peptidase 12 kDa subunit (SPC12)-containing protein
2	Peptidase T
2	Prefoldin subunit-containing protein
2	Putative ferrodoxin
2	Pyridoxamine 5'-phosphate oxidase domain-containing protein
2	Rubrerythrin 2
2	SCP-like extracellular protein
2	SET domain-containing protein

Count	Gene annotation
2	SH3 domain-containing protein
2	SNF7 family protein
2	SPFH domain/Band 7 family protein
2	Sec1 family protein
2	Selenium-dependent molybdenum hydroxylase system protein, YqeB family
2	TB2/DP1, HVA22 family protein
2	Transglutaminase/protease-like domain-containing protein
1	2,3-bisphosphoglycerate-independent phosphoglycerate mutase
1	3' exoribonuclease family protein
1	4-alpha-glucanotransferase
1	4Fe-4S ferredoxin iron-sulfur binding domain protein
1	5'-nucleotidase
1	50S ribosomal protein L12P domain-containing protein
1	ADP-ribosylglycohydrolase
1	ADP-specific phosphofructokinase
1	ARID/BRIGHT DNA binding domain-containing protein
1	Abnormal spindle-like microcephaly-associated protein-like protein
1	Acetyltransferase, GNAT family
1	Actin related protein
1	Actin-related protein 2/3 complex subunit
1	Adaptor complexes medium subunit family protein
1	Adenylyl cyclase-associated protein
1	Aldehyde dehydrogenase
1	Aldose 1-epimerase domain-containing protein
1	Alpha amylase catalytic region
1	Alpha-galactosidase
1	Amino acid permease domain-containing protein
1	Ankyrin repeat-containing protein
1	Annexin
1	Apurinic/apyrimidinic endonuclease
1	Aspartate kinase I domain-containing protein
1	B9 domain-containing protein
1	B9 protein family domain-containing protein
1	BadF/BadG/BcrA/BcrD ATPase family protein
1	Band 7/Mec-2 family protein
1	C-terminal proteasome-interacting domain of thiored-containing protein
1	C2H2-type zinc finger domain-containing protein
1	CAF1 family ribonuclease
1	COPI associated protein
1	CYTH-like domain-containing protein
1	Calcineurin-like phosphoesterase
1	Calcineurin-like phosphoesterase domain-containing protein
1	Calmodulin
1	Calponin homology (CH) domain and Ras family domain-containing protein
1	Caretonoid isomerase
1	Central repeat-containing protein
1	Chromodomain-containing protein
1	Co-chaperone GrpE
1	Cornichon protein
1	Cyclin
1	Cyclin N-terminal domain-containing protein
1	Cyclin, N-terminal domain-containing protein
1	Cystatin domain protein

Count	Gene annotation
1	Cystatin domain-containing protein
1	Cysteine synthase
1	DHHC zinc finger and transmembrane domain-containing protein
1	DHHC zinc finger domain and transmembrane domain-containing protein
1	DHHC zinc finger domain-containing protein
1	DNA binding domain-containing protein
1	DNA cross-link repair protein
1	DTW domain-containing protein
1	DUSP domain-containing protein
1	Deoxyuridine 5'-triphosphate nucleotidohydrolase
1	Dual specificity phosphatase
1	Dynactin subunit p22 family protein
1	EAP30/Vps36 family protein
1	ELMO/CED-12 family protein
1	EMP24/GP25L/P24 family/GOLD family protein
1	ESCRT-II complex subunit-containing protein
1	Elongation factor Tu GTP binding and transmembrane domain-containing protein
1	Endonuclease IV
1	Endonuclease/Exonuclease/phosphatase family protein
1	Endonuclease/exonuclease/phosphatase family protein
1	Eukaryotic initiation factor 4E-like protein
1	Eukaryotic translation initiation factor 4E like protein
1	Exosome 3'-5' exoribonuclease complex, subunit Rrp6p
1	F420 ligase family protein
1	F420-0:Gamma-glutamyl ligase superfamily protein
1	FAD binding domain-containing protein
1	FYVE zinc finger domain-containing protein
1	Fe-S protein assembly chaperone HscA domain-containing protein
1	Ferrodoxin
1	Fip1 motif-containing protein
1	FolC bifunctional family protein
1	Formin homology 2 domain-containing protein
1	Frataxin
1	Fructokinase
1	GCN5-like protein 1 (GCN5L1) family member
1	GCN5-related N-acetyltransferase
1	GINS complex subunit Sld5
1	GOLD domain-containing protein
1	Gar1/Naf1 RNA binding region-containing protein
1	Glutathione S-transferase C-terminal-like domain-containing protein
1	Glycerate kinase
1	Got1/Sft2-like family protein
1	HD domain-containing protein
1	HEC/Ndc80p domain-containing protein
1	HECT E3 ubiquitin-protein ligase
1	HECT domain-containing protein
1	HIT zinc finger domain-containing protein
1	Haloacid dehalogenase-like hydrolase family protein
1	Homeobox domain-containing protein
1	Hydrolase, TatD family
1	Initiation factor 2 subunit family protein
1	Ion channel and transmembrane domain-containing protein
1	Isoamyl acetate-hydrolyzing esterase 1 protein

Count	Gene annotation
1	KH domain-containing protein
1	KIF1-binding domain-containing protein
1	Kinetochore protein nuf2
1	L-seryl-tRNA(Sec) kinase
1	LSM domain-containing protein
1	Las1-like family protein
1	MADS-box protein
1	Mannose-6-phosphate isomerase, class I
1	Meckelin and transmembrane domain-containing protein
1	Metallo-beta-lactamase superfamily protein
1	Methyltransferase TRM13 family protein
1	Methyltransferase domain-containing protein
1	Mib_herc2 domain-containing protein
1	Mitochondrial import inner membrane translocase subunit TIM14
1	Multidrug resistance protein
1	NAD dependent epimerase/dehydratase
1	NADH pyrophosphatase
1	NUDIX hydrolase
1	Neutral sphingomyelinases (nSMase) family protein
1	Nitroreductase
1	Nrap family protein
1	Nuclear transport factor 2
1	Nuf2 family protein
1	O-phosphoseryl-tRNA(Sec) selenium transferase
1	OTU-like cysteine protease domain-containing protein
1	PCI domain-containing protein
1	POP1 domain-containing protein
1	PPPDE putative peptidase domain protein
1	PPPDE putative peptidase domain-containing protein
1	Paired box domain-containing protein
1	Pantothenate kinase
1	Peptidase family M50 protein
1	Peptidyl-dipeptidase
1	Peptidyl-prolyl cis-trans isomerase
1	Phage/plasmid primase
1	Phosphatidate cytidylyltransferase domain-containing protein
1	Phosphatidylserine decarboxylase
1	Phosphopantetheine adenylyltransferase
1	Plasma-membrane choline transporter
1	Pominin domain-containing protein
1	Proteasome activator pa28 beta subunit-containing protein
1	Protein tyrosine kinase domain-containing protein
1	Protein tyrosine phosphatase
1	Putative ATP synthase
1	Putative RNA polymerase I specific transcription initiation factor RRN3 family protein
1	Putative RNA polymerase Rpb4 protein
1	Putative glycine cleavage system H protein
1	Putative nuclear pore protein
1	Putative plastin
1	Pyrroline-5-carboxylate reductase
1	RING finger domain-containing protein
1	RING finger-containing protein
1	RNA polymerase III RPC4 family protein

Count	Gene annotation
1	RNA polymerase Rpc34 subunit
1	RNAse P Rpr2/Rpp21/SNM1 subunit domain-containing protein
1	Rad50 zinc hook motif-containing protein
1	Ran-interacting Mog1 protein domain-containing protein
1	Rap/ran-GAP family protein
1	Ras family domain-containing protein
1	Ras guanine nucleotide exchange factor
1	Ras-like GTPase superfamily protein
1	RhoGAP domain-containing protein
1	Rhodanese-like domain-containing protein
1	Rhodopsin-like GPCR transmembrane domain-containing protein
1	Ribonuclease H
1	Ribonuclease Z
1	Ribophorin I family protein
1	Ribosomal protein L29e
1	Ribosomal protein L39
1	Ribosomal protein S19e*
1	Ribosomal protein S30
1	STAS domain-containing protein
1	Sec7 domain-containing protein
1	Sedlin N-terminal conserved region-containing protein
1	Serine acetyltransferase
1	Serine hydroxymethyltransferase
1	Signal peptide-containing protein
1	Signal recognition particle receptor beta subunit
1	Sugar (and other) transporter family protein
1	Surfeit locus protein 6 family protein
1	Synaptobrevin
1	TATA-binding protein (TBP)
1	TB2/DP1 and transmembrane domain-containing protein
1	TLD domain-containing protein
1	TLD family protein
1	Tenascin
1	Thermophilic metalloprotease (M29) family protein
1	ThiF family protein
1	Thiamin pyrophosphokinase
1	Thioredoxin-like domain-containing protein
1	TolA and transmembrane domain-containing protein
1	Transcription elongation factor Ef1 like domain-containing protein
1	Transcriptional coactivator p15
1	Transcriptional coactivator p15 domain-containing protein
1	Translation machinery associated TMA7 domain-containing protein
1	Type IIB DNA topoisomerase domain-containing protein
1	UDP-N-acetylglucosamine diphosphorylase
1	UDP-N-acetylglucosamine transferase subunit ALG14
1	UEV domain-containing protein
1	Ubiquitin carboxyl-terminal hydrolase domain-containing protein
1	Ubiquitin family domain-containing protein
1	Ulp1 protease family protein
1	Uridine kinase domain-containing protein
1	V-SNARE N-terminal domain-containing protein
1	VMA21-like and transmembrane domain-containing protein
1	Vacuolar ATP synthase subunit E

Count	Gene annotation
1	Vacuolar-sorting protein SNF8
1	Von Willebrand factor type A domain-containing protein
1	WD domain, G-beta repeat-containing protein
1	WD40 domain-containing protein
1	YL1 nuclear protein C-terminal domain-containing protein
1	YbaK/prolyl-tRNA synthetases associated domain containing protein
1	YkwD family and domain-containing protein
1	Zinc carboxypeptidase-containing protein
1	Zinc finger C-x8-C-x5-C-x3-H type domain-containing protein
1	Zinc finger, C3HC4 type (RING finger) domain-containing protein
1	Zinc-finger of C2H2 type-containing protein
1	[FeFe]-hydrogenase assembly protein HydE
1	[FeFe]-hydrogenase assembly protein HydF
1	[FeFe]-hydrogenase assembly protein HydG
1	mRNA capping enzyme, beta chain
1	pfkB family carbohydrate kinase, putative
1	tRNA (5-methylaminomethyl-2-thiouridylate)-methyltransferase
1	tRNA splicing endonuclease

*) Ribosomal protein S19e is annotated in *G. intestinalis* P15, but not *G. intestinalis* WB which was used for comparison.
