# Supplementary material for: The Genome of Spironucleus salmonicida Highlights a Fish Pathogen Adapted to Fluctuating Environments
Source: PLoS Genet. 2014 Feb 6;10(2):e1004053. doi: 10.1371/journal.pgen.1004053 (PMC3916229; doi:10.1371/journal.pgen.1004053)
Supplement: Table S2 — The kinome of G. intestinalis and S. salmonicida. (PDF) [file pgen.1004053.s012.pdf]

Table S2 The kinome of *G. intestinalis* and *S. salmonicida*.

Group	Family	Subfamily	*S. salmonicida*	*G. intestinalis*
AGC	Akt	None	2	1
AGC	AktR	None	1	0
AGC	NDR	NDR-Unclassified	1	1
AGC	PDK1	None	1	1
AGC	PKA	None	1	2
AGC	PTF	FPK	0	1
AGC	RSK	RSK-p3	1	0
CAMK	CAMK-Unique	None	1	1
CAMK	CAMK1	None	0	1
CAMK	CAMK1	CAMK1c	2	0
CAMK	CAMKL	AMPK	8	3
CAMK	CAMKL	CAMKL-Unclassified	0	2
CAMK	CAMKL	CIPK	0	1
CAMK	CAMKL	MARK	1	0
CAMK	CAMKL	NuaK	1	0
CAMK	CAMKL	QIK	1	0
CK1	CK1	CK1-D	2	1
CMGC	CDK	CDK-Unclassified	6	3
CMGC	CDK	CDC2	3	3
CMGC	CDK	CDK5	1	1
CMGC	CDK	CDK2	1	0
CMGC	CDKL	None	2	1
CMGC	CK2	None	1	1
CMGC	CLK	None	2	1
CMGC	CMGC-GL1	None	0	2
CMGC	DYRK	DYRK2	3	3
CMGC	DYRK	DYRK1	2	1
CMGC	GSK	None	3	2
CMGC	MAPK	Erk7	2	1
CMGC	MAPK	ERK1	1	1
CMGC	RCK	MAK	3	2
CMGC	RCK	MOK	2	1
CMGC	SRPK	None	1	1
Other	Aur	None	1	1
Other	Bud32	None	1	1
Other	CAMKK	None	0	1
Other	CAMKK	CAMKK-Unclassified	2	0
Other	CDC7	None	3	1
Other	Ciliate-D1	None	2	0
Other	Ciliate-E2	Ciliate-E2-Unclassified	8	0
Other	IKS	None	0	1
Other	NAK	NAK-Unclassified	2	2
Other	NAK	GAK	1	0
Other	NEK	NEK-Unclassified	1	148
Other	NEK	Nek-GL4	0	51
Other	NEK	NEK-GL1	0	10
Other	NEK	NEK-GL3	0	4
Other	NEK	NEK-GL2	0	3
Other	NEK	NEK1	8	1
Other	NEK	NEK-GL5	4	0

Group	Family	Subfamily	*S. salmonicida*	*G. intestinalis*
Other	NEK	NEK-GL6	3	0
Other	NEK	NEK8	2	0
Other	Other-GL1	None	0	3
Other	Other-Unique	None	10	8
Other	PEK	GCN2	1	1
Other	PLK	PLK1	0	1
Other	PLK	PLK-Unclassified	4	0
Other	SCY1	None	1	1
Other	TTK	None	1	1
Other	ULK	ULK	6	1
Other	ULK	Fused	1	1
Other	Uni1	None	1	1
Other	VPS15	None	0	1
Other	WEE	WEE-Unclassified	3	1
Other	WNK	None	1	1
PKL	CAK	ChoK	1	1
PKL	CAK	FruK	0	1
PKL	PIK	PI3K	1	2
PKL	PIK	PI4K	2	1
PKL	PIKK	FRAP	2	1
PKL	PIKK	PIKK-Unclassified	0	1
PKL	PIKK	ATR	1	0
PKL	RIO	RIO1	1	1
PKL	RIO	RIO2	1	1
STE	STE11	CDC15	1	2
STE	STE11	STE11-Unclassified	1	1
STE	STE20	FRAY	1	1
STE	STE20	MST	1	1
STE	STE20	PAKA	0	1
STE	STE20	YSK	0	1
STE	STE7	MEK1	0	1
STE	STE7	STE7-Unclassified	1	0
TKL	LISK	LISK-DD1	1	0
