# Supplementary material for: The Genome of Spironucleus salmonicida Highlights a Fish Pathogen Adapted to Fluctuating Environments
Source: PLoS Genet. 2014 Feb 6;10(2):e1004053. doi: 10.1371/journal.pgen.1004053 (PMC3916229; doi:10.1371/journal.pgen.1004053)
Supplement: Table S3 — Identified protease families in G. intestinalis and S. salmonicida. (PDF) [file pgen.1004053.s013.pdf]

Table S3 Identified protease families in *G. intestinalis* and *S. salmonicida*.

Catalytic type	Clan	Family	*S. salmonicida*	*G. intestinalis*
Aspartic	AA	A28	0	2
Aspartic	AD	A22	0	1
Cysteine	CA	C1	33	27
Cysteine	CA	C12	2	0
Cysteine	CA	C19	11	6
Cysteine	CA	C88	1	0
Cysteine	CD	C13	0	1
Cysteine	CD	C50	1	1
Cysteine	CE	C48	1	1
Cysteine	CO	C40	1	0
Metallo	MA	M3	1	0
Metallo	MA	M41	5	5
Metallo	MA	M49	1	2
Metallo	MC	M14	0	1
Metallo	MC	M14	5	5
Metallo	ME	M16	0	1
Metallo	ME	M16	2	2
Metallo	MG	M24	1	1
Metallo	MG	M24	3	2
Metallo	MH	M18	4	0
Metallo	MH	M20	2	0
Metallo	MH	M20	3	2
Metallo	MK	M22	1	1
Metallo	MM	M50	1	0
Metallo	MP	M67	2	2
Metallo	MQ	M29	2	0
Threonine	PB	T1	14	14
Serine	SB	S8	0	1
Serine	SC	S9	0	2
Serine	SC	S28	3	2
Serine	SF	S26	1	1
Serine	SP	S59	2	2
Serine	ST	S54	2	1
Metallo	unassigned	M79	0	1
Inhibitor	ID	I4	3	1
Cysteine	None	None	0	1
Metallo	None	None	1	2
Unknown	None	None	8	3
