# Supplementary material for: The Genome of Spironucleus salmonicida Highlights a Fish Pathogen Adapted to Fluctuating Environments
Source: PLoS Genet. 2014 Feb 6;10(2):e1004053. doi: 10.1371/journal.pgen.1004053 (PMC3916229; doi:10.1371/journal.pgen.1004053)
Supplement: Table S4 — Cysteine-rich proteins in S. salmonicida. (PDF) [file pgen.1004053.s014.pdf]

Table S4 Cysteine-rich proteins in *S. salmonicida*.

ID	Group	MW	Cys%	CXXC	CXC	Transmembrane	C-terminal sequence
SS50377_15953	CRMP-1	64.1	17	40	3	VIAVLVVAGGAGGVLTYYFI	KKAKK
SS50377_16104	CRMP-1	141.9	16	51	17	IVGMIVACITAVGFITGVVVFYA	KRPKKYTFQHAITQ
SS50377_17513	CRMP-1	75.8	16	41	6	AGIVIAVLVVVGAVGGGIAYYFI	KKGKK
SS50377_18003	CRMP-1	64.4	16	35	5	VTGIVIAVLVVAGGVGGGLAYYF	IKKGKK
SS50377_18067	CRMP-1	48.0	16	29	3	VTGIVIAVLVVVGAVGGGLAYYF	IKKGKK
SS50377_10628	CRMP-1	52.4	15	18	6	IIGMVFALIIIICGILGIIIYYV	RRPKRHTFQHSMAR
SS50377_11345	CRMP-1	74.4	15	35	5	TVGIVVAVLVIIMLSIGVGIFVV	KKVKRNNKKIEGLVSGEVTQGQVLKIGASSPQMRDQSDQNY
SS50377_11553	CRMP-1	36.9	15	16	2	TAITIAALVVICGVGNCILLYFL	KKARKQQLK
SS50377_jh035	CRMP-1	37.8	15	19	2	AGIVIAVLTVAGAVGGGLAYYFV	KRARK
SS50377_11557	CRMP-1	36.9	15	16	2	TAITIAALVVICGVGNCILLYFL	KKARKQQLK
SS50377_11942	CRMP-1	140.3	15	51	17	IAGMVVAAVAAVGIIAGVSIFYA	KRPKKYSFQHNMAK
SS50377_11947	CRMP-1	140.3	15	51	17	IAGMVVAAVAAVGIIAGVSIFYA	KRPKKYSFQHNMAK
SS50377_13232	CRMP-1	37.9	15	19	2	AGIVIAVLVVAGAVGGGLAFYFI	KKGKK
SS50377_13269	CRMP-1	139.8	15	51	17	IAGMVVAAVAAVGIIAGVSIFYA	KRPKKYSFQHSMGK
SS50377_13274	CRMP-1	45.2	15	17	5	ITGMVVAAVAAVGIIAGVSIFYA	KRPKKYSFQHSMAK
SS50377_13872	CRMP-1	34.5	15	19	2	VTGIVIAVLVVAGGVGGGLAYYF	IKRAKK
SS50377_13874	CRMP-1	68.1	15	38	5	IAGIVGGILVVIGVVSVGLAVYL	KKQKKQKKFNSISTSDNNVEPLSNVLW
SS50377_14561	CRMP-1	139.8	15	51	17	IAGMVVAAVAAVGIIAGVSIFYA	KRPKKYSFQHAMIE
SS50377_14697	CRMP-1	34.6	15	19	2	AGIVIAVLVVVGAVGGGLAYYFI	KRAKK
SS50377_14705	CRMP-1	34.4	15	19	2	VTGIVIAVLVVVGAVGGGLAYYF	TKKAKK
SS50377_15964	CRMP-1	47.6	15	26	0	IIGIVVAVIILTICVVAGLTIFI	IKRKKKSVVVAKSDLVNSNTRSQINRSMSWNNSKIQ
SS50377_15974	CRMP-1	44.3	15	15	5	IIGMVFALIIIICGILGIIIYYV	RRPKRHTFQHSMAR
SS50377_16904	CRMP-1	34.5	15	19	2	AGIVIAVLVVVGAVGGGLAYYFI	KKGKK
SS50377_17084	CRMP-1	34.7	15	16	2	AGIVIAVLVVVGGVGGGLAYYFV	KKAKK
SS50377_17101	CRMP-1	34.9	15	16	2	ITGIVIAVLVVVGAVGGGLAYYF	IKKSKK
SS50377_17215	CRMP-1	47.7	15	26	0	IIGIVVAVIILTICVVAGLTIFI	IKRKKKTVVVAKQDLVKNNSRTYINGSMSQNGSKIQ
SS50377_17480	CRMP-1	29.8	15	16	2	VTGIVISVLVVVGAVGGGLAYYF	IKKGKK
SS50377_17503	CRMP-1	77.8	15	45	6	IAGIVGGILVVIGVVSVGLAVYL	KKQKKQKKFNSIPTSDNNVEPLSNVLW
SS50377_17515	CRMP-1	61.1	15	27	4	IAGIVIAMMVLIGGIGCGVVYYL	MKRYKK
SS50377_17647	CRMP-1	38.2	15	19	3	AGIVVAVLVVAGAVGGGLAFYFI	KRARK
SS50377_18013	CRMP-1	77.6	15	45	6	IAGIVGGILVVIGVVSVGLAVYL	KKQKKQKKFNSIPTSDNNVEPLSNVLW
SS50377_18027	CRMP-1	47.8	15	26	0	IIGIVVAAIILTICVVAGLTIFI	IKRKKKTVVVAKQDLVKNNSRSQINRSMSWNNSKIQ

ID	Group	MW	Cys%	CXXC	CXC	Transmembrane	C-terminal sequence
SS50377_18092	CRMP-1	34.5	15	19	2	VTGIVIAVLVVVGAVGGGLAYYF	IKKGKK
SS50377_18124	CRMP-1	36.6	15	16	2	TAITIAALVVVCGVGNCILLYFL	KKARKQQLK
SS50377_18125	CRMP-1	37.9	15	19	2	AGIVIAVLVVVGAVGGGLAYYFI	KRARK
SS50377_10050	CRMP-1	31.0	14	12	2	IVGIIVGVVAVIGAVGGGLAYYF	IKKGKK
SS50377_10881	CRMP-1	32.1	14	16	2	TGIVIGVLALVGAIGGGVAFYFI	KKSKR
SS50377_11422	CRMP-1	29.2	14	12	2	IVGIVVGVLVVVGAIGGGLAYYF	IRKAKK
SS50377_11595	CRMP-1	30.7	14	12	2	VAGIVIAVLVVVGAVGGGLAYYF	IKKAKK
SS50377_11940	CRMP-1	49.2	14	16	3	NYLGPAAAVVIFTVCIGVVLYFV	QKRVRKNTKVTATAIN
SS50377_12525	CRMP-1	30.0	14	12	2	VGIVIGALVIVGVVGGGLAYYFI	RKARK
SS50377_13209	CRMP-1	54.5	14	18	4	YLGAAAAIGILTFSVGAVAYIAV	KKARKAAGVAATAIN
SS50377_13230	CRMP-1	32.3	14	13	2	AGIVIAVLVVVGAVGGGIAYYFI	KKSKK
SS50377_13289	CRMP-1	30.7	14	12	2	AGIVVAVLIVVGVVGGGLAYYFV	KKAKK
SS50377_15471	CRMP-1	31.2	14	12	2	IAGIVIGIVAVVGVVGCGVAYYL	VRRAKKLLVSEAQ
SS50377_15526	CRMP-1	30.3	14	12	2	IAGIVIAVLVVVGVVGGGLAFYF	IKKAKK
SS50377_15819	CRMP-1	29.7	14	12	2	IVGIVVGVLAVVGAVGSCLAFYL	IKKAKK
SS50377_16083	CRMP-1	31.0	14	12	2	IVGIIVGVVAVIGAVGGGLAYYF	IKKGKK
SS50377_16398	CRMP-1	29.8	14	12	2	AGIIIATLVVVGAVGGGLAYYFI	RKSKK
SS50377_16474	CRMP-1	29.9	14	12	2	AGIIIGTLVVVAAIGGGLAYYFI	RKSRK
SS50377_17112	CRMP-1	32.3	14	13	2	AGIVIAVLVVVGAVGGGIAYYFI	KKSKK
SS50377_17164	CRMP-1	29.7	14	12	2	IAGIIIGVLVVAGAIGGGLAYYF	IKKARK
SS50377_17168	CRMP-1	30.8	14	12	2	AGIVVAVLIVVGAVGGGLAYYFV	KKAKK
SS50377_17501	CRMP-1	30.4	14	12	2	ITGIVIGGLAVISAAGGGLAYYI	IRKAKK
SS50377_17602	CRMP-1	33.5	14	13	2	AGIVLGAVVVVGAVGGGLAYYFI	KKAKK
SS50377_17619	CRMP-1	29.7	14	12	2	IAGIIIGVLVVAGAIGGGLAYYF	IKKARK
SS50377_17626	CRMP-1	30.5	14	12	2	AGIVVTVLIVVGAVGGGLAYYFV	KKAKK
SS50377_17632	CRMP-1	29.8	14	12	2	IAGIIIGVLVVAGAVGGGLAYYF	IKKARK
SS50377_17757	CRMP-1	30.1	14	12	2	IAGIIIGVLAVASAVGGGLAYYF	IKKARK
SS50377_17799	CRMP-1	32.3	14	16	2	IAGIVVAVLVVLGALGGGLAFYL	IKRSKK
SS50377_17996	CRMP-1	29.9	14	12	2	IAGIIIGVLVVAGAVGGGLAYYF	IKKARK
SS50377_18046	CRMP-1	29.8	14	12	2	IAGIIIGVLVVAGAVGGGLAYYF	IKKARK
SS50377_18052	CRMP-1	30.8	14	12	2	AGIVVAVLIVVGAVGGGLAYYFV	KKAKK
SS50377_18100	CRMP-1	30.2	14	12	2	ITGIVIGGLAVISAAAGGLGYYF	IRKAKK
SS50377_18116	CRMP-1	33.0	14	13	2	AGIVIAVLVVTGAVGGGLAYYFI	KKGKK
SS50377_18126	CRMP-1	32.7	14	13	2	AGIVIAVLVVVGAVGGGLAYYFV	KKGKK
SS50377_18841	CRMP-1	30.0	14	12	2	IVGIVVGVLVVVGAVAGGLAYYF	VRRAKKRVEQQ

ID	Group	MW	Cys%	CXXC	CXC	Transmembrane	C-terminal sequence
SS50377_18844	CRMP-1	29.4	14	12	2	IVGIVVGVLAVVVAVGGGLAYYF	IRKGKK
SS50377_18845	CRMP-1	25.5	14	11	2	IVGIVVGVLVVVGAVGSGLAYYF	IRKAKK
SS50377_18884	CRMP-1	29.3	14	12	2	IVGIVVGVLVVVGAVGGGLAYYF	VRRAKK
SS50377_18917	CRMP-1	30.0	14	12	2	IVGIVVGVLAVVVAVGGCLAYYF	VRRGKK
SS50377_18918	CRMP-1	29.4	14	12	2	IVGIVVGVLVVVGAVGGGLAYYF	IRKAKK
SS50377_18919	CRMP-1	29.6	14	12	2	IVGIVVGVLVVVGAIGGGLAYYF	IRKAKK
SS50377_18923	CRMP-1	30.0	14	12	2	IVGIVVGVLVVVGAIGGGLAYYF	VRKAKKRVEQQ
SS50377_18952	CRMP-1	29.6	14	12	2	LVVVGGVVDGLAYYFVRKGKK	
SS50377_18965	CRMP-1	30.5	14	12	2	IVGIVVGVLVVVGAVGGGLAYYF	IRKAKKRVEQQ
SS50377_18966	CRMP-1	29.6	14	12	2	VLVVVGAVGGGLAYYFVRRAKK	
SS50377_12053	CRMP-1	30.1	13	9	2	ITGVIIGILSVILLEGCIIYFFI	KKTKK
SS50377_12088	CRMP-1	41.9	13	13	4	YLGVIAAVLALISCVCITAYIVV	KKARKSPGVVAISIY
SS50377_12553	CRMP-1	22.3	13	7	2	IAGMVVAAVAAVGIIAGVSIFYA	KRPKKYSFQHSMAK
SS50377_12636	CRMP-1	32.5	13	12	2	AGIVIAVLVVVGAVGGGIAYYFI	KKSKK
SS50377_12864	CRMP-1	41.9	13	13	4	YLGVIAAVLALISCVCITAYIVV	KKARKSPGVVAISIY
SS50377_13120	CRMP-1	31.7	13	12	2	FGIIFSVLAVIGGLGIGIALFLI	KRGKKVKPDVGVTNNLE
SS50377_jh058	CRMP-1	29.1	13	12	1	IAGIVIGGVLAVGAVGGLGWFLV	SRKGRKAPVGRPKW
SS50377_15453	CRMP-1	22.6	13	8	1	IVGIIVGVVAVIGAVGGGLAYYF	IKKGKK
SS50377_15539	CRMP-1	31.8	13	12	2	IAGIIIAVLVVIGAVIGLIILLL	RKNKKTQPISGVKRDLI
SS50377_ja015	CRMP-1	29.1	13	12	1	IAGIVIGGVLAVGAVGGLGWFLV	SRKGRKAPVGRPKW
SS50377_17965	CRMP-1	22.3	13	7	2	ITGMVVAAVAAVGIIAGVSIFYA	KRPKKYSFQHSMAK
SS50377_18031	CRMP-1	30.6	13	12	1	AGIVVAALIVVGVVGGGLAYYFV	KKAKK
SS50377_18882	CRMP-1	31.3	13	11	2	AGIVIGVLLVVGAVGGGLTYYFV	RRAKK
SS50377_18907	CRMP-1	29.8	13	11	2	IVGIVISVLVVVGAVGGGLTYYF	IRKGKK
SS50377_13212	CRMP-1	34.0	12	8	2	SIGIIVSIAVLTICIGVVAYNFA	KKDRKAPRVPGMTIN
SS50377_16406	CRMP-1	34.7	12	12	1	IIAAITIGCLLFICSTVGIILYY	MKKRSKKNQQAVFIEKKLAPAFIFNTDQIMTNK
SS50377_17372	CRMP-1	17.4	12	4	2	VAGILVAVLVVVGAVGGGLAFYF	IKKGKK
SS50377_17639	CRMP-1	34.1	12	10	2	AISGIIIGALAVVGAISGTVIVI	MRKSRKNQVSTDTGAVNILE
SS50377_17646	CRMP-1	37.0	12	13	1	LSQPLVIICGVGNCILLYFL	KKARKQQLK
SS50377_18971	CRMP-1	34.0	12	10	2	AIAGIIIGALAVVGAISGTVIVI	MRKSRKNQVSTDTGAVNILE
SS50377_10079	CRMP-1	32.5	11	9	2	IVGIVLGVVAVAAVVGGGLAYYF	VKKHKKAAGGSATQVQVE
SS50377_11341	CRMP-1	21.8	11	6	1	TGIVIGVILVVGAVAGGLAYYFI	KRGKK
SS50377_11344	CRMP-1	22.0	11	6	1	ITGIVIGAVVVVGAVAGGLAYYF	IKRGKK
SS50377_11556	CRMP-1	32.4	11	9	2	AGIVIAVLAVVGAVGGGLAYYFV	KRARK
SS50377_12850	CRMP-1	71.5	11	19	4	YLGAAAAIGILTFSVGAVAYIAV	KKARKAAGVAATAIN

ID	Group	MW	Cys%	CXXC	CXC	Transmembrane	C-terminal sequence
SS50377_17512	CRMP-1	16.3	11	6	1	AGIVIAVLVVVGAVGGGLAYYFI	KKGKK
SS50377_17746	CRMP-1	18.0	11	4	2	VTGIMIAVLIVVGAVGGGLVYYF	IKRAKK
SS50377_17777	CRMP-1	21.5	11	6	1	VGIVIGALVAVGAIGGGLAFYFI	RRSKK
SS50377_18852	CRMP-1	27.3	11	8	1	AGILIGVLLVVGAVVGGLAYYFV	RRAKKRVEQQ
SS50377_18861	CRMP-1	26.8	11	8	1	AGIVVGVLIVIGAVGGGLAYYFV	RRQKK
SS50377_18908	CRMP-1	37.7	11	12	2	AGIVIGVLLVVGAVGGGLTYYFV	RRAKK
SS50377_18922	CRMP-1	26.7	11	8	1	AGIVIGVLLVVGAVGGGLAYYFV	RRGKK
SS50377_18953	CRMP-1	26.8	11	8	1	AGIVIGVLLVVGAVGGGLTYYFV	RRAKK
SS50377_18967	CRMP-1	18.4	11	6	1	TGIVIGVLLVVGAVGGGLAYYFV	RRGKK
SS50377_18975	CRMP-1	36.5	11	12	2	TGIVIGVLLVVGAIGGGLAYYFI	RKAKKRVEQQ
SS50377_18999	CRMP-1	37.4	11	12	2	TGIVIGVLLVVGAIGGGLAYYFI	RKAKKRVEQQ
SS50377_10017	CRMP-1	23.0	10	6	1	TGIVIGVILVVGAVAGGLAYYFI	KRGKK
SS50377_10032	CRMP-1	22.8	10	6	1	TGIVIGVILVVGAVAGGLAYYFI	KRGKK
SS50377_10639	CRMP-1	23.6	10	6	1	TGIVVGVVLVVGAVAGGLAYYFI	KKGKK
SS50377_10643	CRMP-1	23.6	10	6	1	TGIVVGVVLVVGAVAGGLAYYFI	KKGKK
SS50377_12258	CRMP-1	25.1	10	6	1	VGIVIGALVAVGAIGGGLAFYFI	RRSKK
SS50377_17609	CRMP-1	57.0	10	12	2	IAGIIIGVLVVAGAIGGGLAYYF	IKKARK
SS50377_17890	CRMP-1	15.4	10	3	1	IAGIVIASLVVVGVIGGGLAYYF	IKKLKR
SS50377_18017	CRMP-1	13.0	10	3	1	VTGIVIAVLVVVGAVGGGLAYYF	IKKAKK
SS50377_12257	CRMP-1	23.9	9	6	1	VGIVIGALVAVGAIGGGLAFYFI	RRSKK
SS50377_18957	CRMP-1	22.6	9	6	1	TGIVIGVLLVVGAIGGGLAYYFI	RKAKKRVEQQ
SS50377_10064	CRMP-1	16.6	7	3	1	VGIVLGVVAVAAVVGGGLAYYFV	KKHKKAAGGSATQVQVE
SS50377_12645	CRMP-1	31.9	7	4	2	AGIVIAVLVVVGAVGGGIAYYFI	KKSKK
SS50377_10028	CRMP-2	8.7	23	2	4	AVLCAGICFDLVVIWGWIAGIIV	GVKMMC
SS50377_11351	CRMP-2	9.7	17	3	2	AGASMFFTCFIFWGWIVAIIVGV	KMMCPG
SS50377_11945	CRMP-2	69.7	17	40	0	IIIVVVVCILVLCITAGVIYYIV	KHKKQARSTKSKALHNIQQQDLN
SS50377_14690	CRMP-2	116.1	17	77	9	IAGIVIAVLVVIGLVAGGAFWYV	KRSKHTPAEIGLVDRRLQ
SS50377_15174	CRMP-2	90.5	17	52	0	AIVGILVGVLLVVSVLAGLGVYY	SILKKKKANATVLEQDNVQLATVPVYDGMGNGGTLVKQGFV
SS50377_16496	CRMP-2	89.7	17	52	0	AIVGIVVGVVLAVGVLAGLGAYY	AMSKKKKANVTVIEENSLQPASVSVFAVNSNGGTLVK
SS50377_17603	CRMP-2	65.6	17	40	3	AVTGIIIAVLLVVGGVAGGVFWC	MKKSKVNSEIETSNSNIK
SS50377_17697	CRMP-2	108.4	17	55	5	WQILLSIIVICAILVGIAVWIVY	FKKKKLNKKQDSYMIFPNAEN
SS50377_10984	CRMP-2	68.0	16	40	0	IAGIVAGLVLLVGLIGGAIAFAV	ARKRKAQRSVYGDLEVKKENLQLQVDDDNL
SS50377_10986	CRMP-2	45.4	16	25	0	IAGIVVGLVLLVGLIGGAIAFAV	ARKRKVQCSIQGDLEVKKENIQLQVDDDSLWSKVSK
SS50377_11560	CRMP-2	64.1	16	39	4	AIAGIVIAVLIVASAIGGGVFWF	MRQKKKTSVSVEGSAGIQE
SS50377_11939	CRMP-2	67.8	16	40	0	VIAIIVAVLIVVACVTGGAIFFI	VKRKRDSGRGLHRMATKRDVAGSQDKVSAFSSD

ID	Group	MW	Cys%	CXXC	CXC	Transmembrane	C-terminal sequence
SS50377_11941	CRMP-2	87.3	16	49	9	IIFIVIGIIGGITLTFGAAVFVI	IARKRKSNVNPDYDTTLLNQSNNSSSQLF
SS50377_12594	CRMP-2	63.2	16	34	6	IGIIAGIILLLILIILGITFAVV	KYRKRKEKLKLNDLSMLVRKETSSSQLLL
SS50377_13016	CRMP-2	85.4	16	46	10	QIIYIVLGVIAGVCLIIGIVIVV	KIVKKRKSIKSDEYNSAELLTRNNSSSQIY
SS50377_13303	CRMP-2	64.5	16	39	0	WLLTAVISVIVACIVGGVAYLVV	KMGKNKALRMGIQKDLYEENSGIRTQQPQE
SS50377_13319	CRMP-2	71.1	16	35	6	VISVSIGVVAAIIIIVVAIVIIL	NRAYRNKKYSVNDSNHKLFKKAKTQVLKTACDNLCPE
SS50377_14685	CRMP-2	105.8	16	67	8	IAGIVIAVLVVIGLVAGGAFWYV	KRSKHTPAEIGLVDRRLQ
SS50377_14694	CRMP-2	98.4	16	56	7	TVAGIVIAVLAVVGGVAGGVFWY	MKKSKTNQDIERLNFILK
SS50377_14702	CRMP-2	56.1	16	33	0	IIGIAVGIIAVVGIVIGGVVYAV	QMKKKSNLVQKNSNVKPNKQNIQFQIDESVKILTKTE
SS50377_14714	CRMP-2	75.6	16	47	4	AIAGIIIAVLVVVGGVAGGVFWY	MKKTKVNQETEKQNFSRQ
SS50377_15353	CRMP-2	51.2	16	33	0	IVGIVVGVVAAIGLVAGAGAYFI	VKKRKQAVVSPPKDDEIKHANLQMLQEQNIILQKRQQ
SS50377_15666	CRMP-2	51.0	16	33	0	IVGIVVGVVAAIGLVAGAGAYFI	VKKRKQAVVSPPKDDEIKHANLQMLQEQNIILQKRQQ
SS50377_15947	CRMP-2	82.9	16	46	8	IGLGAVLGLALAVAAVILVIWAV	KKRKARANKDYDTDVVGVRISSSSSQLF
SS50377_15963	CRMP-2	78.5	16	42	7	IAGVVIAVLLVIGGLCVGGFFLA	RRCVGKRNLGNAEPLKIDNTVW
SS50377_15971	CRMP-2	117.5	16	42	14	IVCLLIALIIIIGGVWIIVYHYL	SKPKKYYQQHNILAAQNQQ
SS50377_16105	CRMP-2	115.7	16	42	14	WDVVGCVAAGATIVAIVGYLIYY	YTHRPKKFYTQHNMFSK
SS50377_16470	CRMP-2	89.5	16	52	0	AIVGIVVGVVLAVGVLAGLGAYY	AMSKKKKANVTVIEENSLQPASVSVFAVNSNGGTLVK
SS50377_16581	CRMP-2	61.4	16	34	6	IIGIAVGSVAGLVIVCVIIVIVV	KKSSKKHKMPKRDNDVLIQRTDSNSQLIV
SS50377_16894	CRMP-2	63.9	16	39	4	AIAGIVIAVLIVAGAIGGGVFWF	MRQKKKASVCVEGSAGIQE
SS50377_17167	CRMP-2	73.7	16	46	5	AVAGIVIAVLVVAGGVAGAAFWY	MKKSKANQKVEKQNFNMQ
SS50377_17171	CRMP-2	77.9	16	49	6	AVAGIVIAVLVVAGGVAGAAFWY	MKKSKANQEVEKQNFNMQ
SS50377_17182	CRMP-2	66.2	16	40	3	AVAGIIIAVLLVVGGVAGGVFWC	MKKSKVNSEIETSKSNIK
SS50377_17377	CRMP-2	57.0	16	32	0	IGIVVGIIAVVGIVIGGVVYAVL	MKKKSNLVQKNNNVKPNKQNVYQRIDESATILGKAQ
SS50377_17486	CRMP-2	56.7	16	32	0	MIGIVVGIIAVVGIVIGGVVYAV	QMKKKSNLVQKNSNVKPNKQNIQFQIDESVRILTKTE
SS50377_17489	CRMP-2	71.7	16	38	5	AVAGIVIAVLVVVGGVAGGVFWY	MKKTKVNQEIEKQNFSRQ
SS50377_17491	CRMP-2	63.7	16	39	4	AIAGIVIAVLIVAGAIGGGVFWF	MRQKKKTSVSVEGSAGIQE
SS50377_17598	CRMP-2	59.0	16	35	4	AVTGIIIAGLVVVGGVAGGIFWY	MKKSKLNQEIDEQKFVLK
SS50377_17623	CRMP-2	73.6	16	46	5	AVAGIVIAVLVVAGGVAGAAFWY	MKKSKANQEVEKQNFNMQ
SS50377_17627	CRMP-2	73.3	16	46	5	AVAGIVIAVLVVAGGVAGAAFWY	MKKSKANQEVEKQNFNMQ
SS50377_17750	CRMP-2	59.8	16	35	4	AVTGIIIAVLVAVGGVAGGIFWY	MKKSKLNQEIDEQKFVLK
SS50377_17988	CRMP-2	61.1	16	36	4	AVTGIIIAVLVAVGGVAGGIFWY	MKKSKLNKEIDEQKFVLK
SS50377_18009	CRMP-2	56.7	16	32	0	IGIVVGIIAAVGVIIGGVVYAVL	MKKKSNFVQKNNDATTNKQNIQYQIVESATMLGQTKQ
SS50377_18033	CRMP-2	82.1	16	52	6	AVAGIVIAVLVVAGGVAGAAFWY	MKKSKANQEVEKQNFNMQ
SS50377_18050	CRMP-2	74.5	16	46	6	AVAGIVIAVLVVAGGVAGAAFWY	MKKSKANQEVEKQNFNMQ
SS50377_18057	CRMP-2	55.0	16	32	3	AIAGIVIAVLIVAGAIGGGVFWF	MREKKKTSVCVEGSAGIQE

ID	Group	MW	Cys%	CXXC	CXC	Transmembrane	C-terminal sequence
SS50377_18076	CRMP-2	56.6	16	32	0	IIGIVVGIIAVVGIVIGGVVYAV	QMKKKSNLVQKNSNVKPNKQNIQFQIDESVRILTKTE
SS50377_18081	CRMP-2	83.9	16	52	6	VAGISLAVIAVVLGASGGVFWYL	RRAKIAKRKTVMKWRQQ
SS50377_18085	CRMP-2	82.8	16	53	6	AITGIVITVLIIIGAICFGIFWY	MKKVKITKPLVESGVAIQ
SS50377_18089	CRMP-2	56.3	16	33	0	IGIAVGIIAVVGIVIGGVVYAVL	MKKKSNLVQKNNNVKHNKQNVYQRIDESATILGKAQ
SS50377_18096	CRMP-2	96.2	16	60	7	ITGIVIAVLVVVGLIAGGAFWYV	KRSKHTYSDIGLVGRLQQ
SS50377_10023	CRMP-2	50.1	15	25	5	YIVVGVIAGLTVIMSIVITVLVI	KKRKISNKQQFERSLLEKANNSSNQVF
SS50377_10070	CRMP-2	60.3	15	34	0	AMVGIIICLLVIVGGVVGTAIFI	TQKRKRSVGNNNHVDILSNNEITIQGLQNVVDQKSILNSIKE
SS50377_10747	CRMP-2	38.9	15	19	2	GIIGIVIAILVAVGVVGGVVGCC	MRKKKHTAVVADAETVVAGQQ
SS50377_10751	CRMP-2	51.8	15	23	4	GLSAGVIVGIAVAVVGIYRSGCW	SRYRCLRCCKEARASSDRAGNCYNRRLTIYFTAVNQCAFLQKWCLPKFNCSVTIT
SS50377_12054	CRMP-2	86.4	15	28	9	IVGLVFAGLIIILTVCLIVYQYL	AKPKKYYQQHNLKALQNNQ
SS50377_12837	CRMP-2	52.3	15	21	7	AIVGIVLGVLGLVGALVILIVVV	MKKRAARGNGQDIQEELVVDSKYYSQFSEVSIASMIE
SS50377_12885	CRMP-2	70.2	15	21	7	EIAGIALAVLCGALLVVVVVVAV	KCSARKRQSGPWK
SS50377_13121	CRMP-2	84.9	15	36	6	IQITAIILSVVAIVIVIGSTITL	IQKYKRRITRVAKVETYYN
SS50377_13277	CRMP-2	38.9	15	19	2	GIIGIVIAILVAVGVVGGVVGCC	MRKKKHTAVVADAETVVAGQQ
SS50377_13717	CRMP-2	115.4	15	41	13	WDVVGCVAAGATIVAIVGYLIYY	YTHRPKKFYTQHNMFSK
SS50377_14187	CRMP-2	38.9	15	19	2	GIIGIVIAILVAVGVVGGVVGCC	MRKKKHTAVVADAETVVAGQQ
SS50377_14533	CRMP-2	59.2	15	29	4	TIAGIVIAVLVVVGGAGVIFWYI	KKSKVNQEIEMPKSNIK
SS50377_14537	CRMP-2	41.2	15	21	1	VAGIVIAIIVIIGGIGGGVFWYL	KKSKTQLKNVSQGGVMQE
SS50377_14594	CRMP-2	55.4	15	30	5	ITGIVITVLIILGLISGGVFWFI	KKSKSEQYAIKDEVIVNQI
SS50377_14605	CRMP-2	43.0	15	21	3	GAGISAIVFACLWVIGGIAYGIF	RYKKFCKIERAFISGKPEKIE
SS50377_15967	CRMP-2	48.2	15	22	0	AIVGTIAGMLLVVSVLAGLGVYY	SIIKKKKVGAPVLEQDNVKLATVPMYAGIGNGGTLVK
SS50377_16110	CRMP-2	122.5	15	42	9	IAGVVIGVGIGIVAMAAISIWCL	CKNGSRKARFVDQGLMQGENGSQVAIVD
SS50377_ja020	CRMP-2	42.1	15	21	5	ITGIFVGVLAVIAITILVVVLIL	KSMRNKKKAPQLDLPVQRMTGTPHAHDMS
SS50377_16896	CRMP-2	62.0	15	30	4	AVAGIVIAVLIVVGGVAGGAFWY	MKKSKVNQEIEKQNFSRQ
SS50377_16901	CRMP-2	50.5	15	29	0	IGIVVGIVALVGVIIVGVVYAVL	MKKKSNFIQKNNDATTNKQNIQYQIVESATMLEQTKQ
SS50377_17196	CRMP-2	140.2	15	51	8	GIAGIVIALIAVLLVVILTVILV	KRRKSNAKRAQYVDQGLLDRGNGSQVAIVE
SS50377_18072	CRMP-2	32.7	15	13	2	VFNILLGTLVALCIIWGMLDIIL	CKKIKNKKQ
SS50377_18086	CRMP-2	32.4	15	13	2	VFNILLGTLVALCIIWGMLDIIL	CKKIKNKKQ
SS50377_18105	CRMP-2	61.3	15	36	4	AIAGIVIAVIVVVAAIGGGVFWY	MKKSKASSTTTRSNAAMQ
SS50377_19076	CRMP-2	52.4	15	26	6	ITGLIIGAFILIVTVVIVIIGFV	RRSNEIKYSVYKIKAQQRAEELEKSIVENNDE
SS50377_10265	CRMP-2	72.3	14	22	6	DILSILFGLVLAAIIVVFGVFWF	KKCNRSKRQDMVDNRNIFI
SS50377_10641	CRMP-2	32.6	14	12	2	AIAGIIIGVLIVVGGLCFATFWF	MKKKVSTAPVQVRNDFE
SS50377_11709	CRMP-2	62.9	14	18	6	MEIAGIALAVLCGALLVVVVVAV	KCSARKRQSEPWK
SS50377_11828	CRMP-2	31.4	14	12	2	GAIAGIVIAVLVVAGAVGAGVWF	MMKKKRCIGSAGTRHGRK

ID	Group	MW	Cys%	CXXC	CXC	Transmembrane	C-terminal sequence
SS50377_11948	CRMP-2	30.0	14	12	2	ITGIIVAILIVVGGLCVGTFLFL	KKRANSAPVKIINNIE
SS50377_12075	CRMP-2	58.1	14	21	4	AIGVIFTLVVLTGSVGAILYAIL	KKRNKAAVAVDTATN
SS50377_12085	CRMP-2	10.5	14	2	2	AGLYMFLFAPLLVGWGLSVVVGV	RMMLQ
SS50377_12213	CRMP-2	31.6	14	12	2	IGIVMAILCGVLGVLGVMVFLYF	QKKKGLVGEGYHQ
SS50377_12582	CRMP-2	58.1	14	21	4	AIGVIFTLVVLTGSVGAILYAIL	KKRNKAAVAVDTATN
SS50377_13286	CRMP-2	50.2	14	23	3	AVAGIVIGVLVVAALAIGCVVYF	RMRKRAPAATPLELSHQTD
SS50377_14141	CRMP-2	61.4	14	31	0	IAGIVVGLVLLVGLIGGAIAFAV	ARKRKAQRSVQGDLEVKKENLQLQVDDDSLWSKVSK
SS50377_14189	CRMP-2	47.0	14	22	3	GEVAGIVIGVLVVVGVISGVVVF	QMKKKHSTVLTSEKVAHQND
SS50377_14193	CRMP-2	43.2	14	19	4	AIIGIVVVVILIVCACIGAVMVY	KKRKLEKAETPLNVSELSN
SS50377_14386	CRMP-2	65.3	14	29	0	MIIGIVSVVGLVLILSFLIAFFV	CKRVLLVKVQQNVETIKVENIDSNAIQIGIQ
SS50377_14590	CRMP-2	44.1	14	13	4	VIGCSIAIAILLAVIVVLVVFLV	RKMSVKAPTVVRDFHGIQ
SS50377_14630	CRMP-2	43.3	14	20	2	TITNTEFVFLALTAVWVAYIVFL	SMKHHRAQRQQRAERIQNHTFQLSNKLANTSFGWG
SS50377_14699	CRMP-2	34.3	14	13	2	VFNILLGTLVALCIIWGMLDIIL	YKKIKIKSSEEFVILNKQKLSNT
SS50377_15275	CRMP-2	79.3	14	40	0	VVGVSIGCIMAILLLGIILIVVW	KKLQKKIQPIPPFSASLEYENLVINQ
SS50377_15292	CRMP-2	30.5	14	12	2	IAGIIIAIIILIGAVGGGLAYYF	TKRAKS
SS50377_15527	CRMP-2	32.0	14	12	2	IVTIVVIVMIAAGLIGCGIFILI	WKNKQTATATGVVKDHV
SS50377_ja042	CRMP-2	45.7	14	14	4	VLLWQILFGVSIVLLSGLLCILL	WRYLYNKINHEKEGQRLIGVQLQEQ
SS50377_15956	CRMP-2	32.4	14	12	2	AITGIIIAVVIVVGVVCVGTFLF	MKKKVYNVAVEIRNNLK
SS50377_16377	CRMP-2	29.3	14	12	2	IAGIVIGVLAVTGAVGGGLAYYF	TMRSKKYLLLK
SS50377_16381	CRMP-2	44.0	14	14	4	AVVNRLGAGSIAGIAVGVALGVC	VLAAAAVMVVRAVKSRAVAKVERDIAYI
SS50377_16520	CRMP-2	31.5	14	12	2	GAIAGIVIAVLVVAGAVGAGVWF	MMKKKRCIGSAGTRHGRK
SS50377_17006	CRMP-2	61.3	14	19	6	WTVAGISIAVFLAIALIAILVGL	WMFGYLRKSQAEAVMEETITETPDVQTGRNNGNNLLPPVEAGKDVELGKIALEQK
SS50377_17251	CRMP-2	69.8	14	20	6	VILIGIFGFLLACLIIGLVVLLI	KNRVRKPQLLNRMATSADLLDAEYEIGSAAQ
SS50377_17383	CRMP-2	31.3	14	16	2	AVTGIIIAVLVAVGGVAGGIFWY	MKKSKLNQEIDEQKFVLK
SS50377_17538	CRMP-2	40.0	14	14	3	GGIVGIVIAVVVVIGVIGGVVYF	MKKKNSRRVPDDIQTKFN
SS50377_17648	CRMP-2	32.6	14	13	2	AGIVIAVLVVVGAVGGGLAYYFV	KKGKQ
SS50377_18270	CRMP-2	73.6	14	32	0	AAGLGMIVIAVIIIAVVVGAMLV	RKMRSKAQPEASSGYAIN
SS50377_18298	CRMP-2	64.7	14	28	0	TAGIGLIVITVLILAVMAGVMLV	RKIRSKAQPEASSGHAIN
SS50377_18883	CRMP-2	29.6	14	12	2	IVGIVIGVLVVVGAVGGGLAYYF	VRMGKK
SS50377_11190	CRMP-2	13.6	13	5	3	LAGFVMWLTSSFVVGWVVACIVG	CQICYN
SS50377_12385	CRMP-2	16.6	13	6	2	IAGIILGTLACICGVGAIVFFVI	KNKRCKTIP
SS50377_12597	CRMP-2	31.1	13	10	2	VGIIIGVLAVVGAISGIVMVIII	KSKKNQVK
SS50377_12632	CRMP-2	13.3	13	6	3	VCLSGIVMWLTAQFVVGYVMACI	VGCKMCYGG
SS50377_12857	CRMP-2	60.0	13	9	8	LIAGIVILLLVIIIIIGGTVYYF	TKKRNQISSANSIIIPKKDSGIQEMKTTRHSGLVTENEQYAK

ID	Group	MW	Cys%	CXXC	CXC	Transmembrane	C-terminal sequence
SS50377_13172	CRMP-2	32.5	13	12	2	AGIVVGVLVVLGTVGGGLTYYFI	KKSKTQKFQAGMEESTSKQAPE
SS50377_14481	CRMP-2	30.8	13	12	1	IAGIAVGSILVIGIAVGTVCYFL	KRKSRFAQNQTQAVKDTTMDTVFGGQTVMRSQ
SS50377_14530	CRMP-2	28.6	13	14	2	AIAGIVIAVLIVAGAIGGGVFWF	MRQKKKTSVSVEGSAGIQE
SS50377_15311	CRMP-2	56.9	13	24	4	WIIGISVTGCLLLCTLIVGLIVW	FVRRGKIGIYENQMSDATLSRFWLAQSLITVTMRFTISRFSDEIVRIN
SS50377_15343	CRMP-2	31.2	13	10	2	AGIVVGVALIVLAVGGGLAYYFI	NKQKK
SS50377_15525	CRMP-2	31.4	13	11	2	IAGIVIAIFVVVGAISDGLAFYF	IKKSKTNGMTE
SS50377_17160	CRMP-2	42.3	13	15	0	TIGAVLGGVVALTAVIGVVAFAV	RSRKQRMHTEPPPRAEE
SS50377_17208	CRMP-2	34.7	13	14	1	GIAGIVIAILAVLLLVTLVVILI	RRRKSTAVRAKLPDGGLLEGHNGSQVAVVD
SS50377_17553	CRMP-2	46.3	13	16	2	VIIGFFICVIILIITAAIIIFLI	QKNRLGQYKKIPDGIKTLKKQNCTKLRKGNISSNGNLSTNCVSYLPPETKADDNIWKKTK
SS50377_17874	CRMP-2	59.6	13	9	8	LIAGIVIMALIVVALVGLGVYFF	MKKRNDIPRRNSIVMPLRDAPIHRMNSNNSTALVTENPEYAK
SS50377_18002	CRMP-2	27.2	13	10	2	AVVGIVIAVMVVVGGVAGGVFWF	MKKSKVNQEIEKQNFSRE
SS50377_18006	CRMP-2	26.8	13	10	2	VAGIVIAIIVIIGGIGGGVFWYI	KKSKTQLKNVGQGEVMQE
SS50377_18008	CRMP-2	41.6	13	12	2	LIANTANFAIYQTLGILIFVSCF	L
SS50377_18016	CRMP-2	26.9	13	10	2	VAGIVIAIIVIIGGIGGGVFWYI	KKSKTQLKNVGQGEVMQE
SS50377_18080	CRMP-2	56.5	13	26	0	IIGIVVGIIAVVGIVIGGVVYAV	QMKKKSNLVQKNSNVKPNKQNVYQRIDESATILGKAQ
SS50377_18676	CRMP-2	32.7	13	4	5	YGILLGAGAGVLVTAGLLIYFIR	NSTFGYKRVTGE
SS50377_10698	CRMP-2	27.7	12	8	2	AIGVIFTLVVLTGSVGAILYAIL	KKRNKAAVAVDTATN
SS50377_fx024	CRMP-2	20.2	12	6	1	ALIIFLSLEVIWGVLGMVSYFGA	KRLITIRDVKAQIQAQSDTKNE
SS50377_13205	CRMP-2	27.1	12	10	1	TITNTEFVFLALTAVWVAYIVFL	SMKHHRAQRQQRAERIQNHTFQLSNKLANTSFGWG
SS50377_14554	CRMP-2	31.7	12	8	2	VAGIVVVVIAISGGISGGIFWYL	KKSKTYMKNVDQVGIRQE
SS50377_14603	CRMP-2	32.7	12	13	2	WIIGISVTGCLLLCTLIVGLIVG	LSEGANRNIRESDE
SS50377_15512	CRMP-2	36.6	12	11	2	GIITGIVITIIIVVTGVAATITY	QIIRKLKK
SS50377_15515	CRMP-2	52.2	12	8	7	LFAMLIVIALSLVAIMSIGFYLF	ARKYLIKHTYSITDNQIESEDKYNPELIISHQVACLNSGDSV
SS50377_15940	CRMP-2	36.5	12	13	0	TIGAVLGGVVALTAVIGVVAFAV	RSRKPRMHTELTLVQRNEI
SS50377_15950	CRMP-2	30.1	12	10	2	AIAGITIAVVIVVGVVCVGTFLF	MKKKVYNVAVEIRNNLK
SS50377_17132	CRMP-2	30.7	12	9	2	GAIIAIIAVVAIIAVCVILGCGF	AAKAYRKRQKSIKDGAERCGAERSETGVEAAVHIA
SS50377_17543	CRMP-2	48.8	12	16	2	TIGFLLCFLISLVGAGIIITLYW	KQRREKYYKLENYIIDQNFNTKSRFQLANNSKVVQLKNRQEKILQGNENQIENMLETPALSGEI QIDLSDNTINDKVQ
SS50377_17954	CRMP-2	48.9	12	16	2	IIGFFICLLILIIAAITIIFLFW	KQKRNIYYKIENYGKEEDISTKDRFQFVNASKIFNKSQENFQPTKGMKKQLSFKFNKPETRDKT TIDLTESVLPDNEE
SS50377_17982	CRMP-2	33.4	12	10	2	IAGITIAVVIVVGVVCVGTFLFL	KKKFYNVAVEIRNNLK
SS50377_18070	CRMP-2	17.1	12	4	2	VAGILVAILVIVGAVGGGLAFYF	IKK
SS50377_18266	CRMP-2	39.5	12	17	0	IIGIVIAVILVAAGFAVFGVYLF	KKYKQNKASTLIKDQVQAQIGSQYEV
SS50377_18273	CRMP-2	38.2	12	16	0	IIGIIIVIVVVILAIVIITLYCW	RKVIQNRVLPFENNEDLLNIDSQMEQ
SS50377_18685	CRMP-2	33.6	12	9	2	WVIALVIGIILIIICIGTIVFKI	LHKKRQKTKAGVVETNVISLSDQLTACFDQTEIPEKLV

ID	Group	MW	Cys%	CXXC	CXC	Transmembrane	C-terminal sequence
SS50377_18842	CRMP-2	33.4	12	12	2	LGGWVLTVFLEFGIIRIVGIIIL	KLKLK
SS50377_18905	CRMP-2	27.1	12	12	0	IVGVTIGLILGLVVLGVAVYFVW	RNRIKNHHGLNKSNAVEHGEMMLQVFREE
SS50377_10896	CRMP-2	23.0	11	6	1	ITGIVIGSLLFLLLLILLIIYII	WRNRKQNQDEEGIIEECNGDPILLETK
SS50377_12087	CRMP-2	33.0	11	5	3	GMVVAIVVLVAVAVFQVCAIAVY	QLRQRGIPTRRNSILSSRTVSISQRTKSNDFQGLKMDSYYAE
SS50377_12129	CRMP-2	20.2	11	3	2	HLYAAFQLAIPIFLFLLLLTIQA	LPCDPAQKRQKFTCRPSDCALCPFAACEYSPKRQKFACKCGNSSGKFCEICPENALFDAIFGCF VAENCGRGRFNVRKGRCECDNGYGGKGCAQCSRGFEMVDLEGRQEGWACFESVRCEIGFRKVIS GNQLVCELNACENGVVWGDFCVVGN
SS50377_12863	CRMP-2	33.0	11	5	3	GMVVAIVVLVAVAVFQVCAIAVY	QLRQRGIPTRRNSILSSRTVSISQRTKSNDFQGLKMDSYYAE
SS50377_13234	CRMP-2	26.6	11	6	1	IVVMCVIILICLIGAAISCV	EKRLQTAASSRKTIIYTQPWQDYE
SS50377_ee043	CRMP-2	57.7	11	17	3	VIVGIAVAVVVFIGLVVGVVIGV	CAVAKRREPAPIAPEIVITAD
SS50377_16519	CRMP-2	19.6	11	2	2	GSIIGLCVSVCVIAGAIVGVVAY	RVIKHKQGQKQKAETEKTPLVGEGSAEKSQSA
SS50377_ja068	CRMP-2	32.2	11	10	1	LIANTANFAIYQTLGILIFVSCF	L
SS50377_17287	CRMP-2	79.3	11	18	4	LLFVILVLLVFTWQINFVLV	RHQIIFIIFGCFYFYYQLQQQYLQYLQDNNTSQSYSDTNLKLTSTEQYVSFSRYFVEFSFVKQQ LFVLRYRTRTWMIYIFTLPLQVSAHSHLPSCCSSYRLPIWINSTTTRYRTSSCSCSGRGWTSCS RLLYIYYYQKRCPCPRPSSSASQRHFPCCCLRSF
SS50377_17502	CRMP-2	30.9	11	8	1	GTIAGIVISALVVVGGWMTYYFI	RTVRK
SS50377_17783	CRMP-2	18.1	11	6	0	IGVISGVTIATVVALGALCFLVW	RELRKNRILAVDAAGTRPGSLALD
SS50377_17984	CRMP-2	18.0	11	4	2	VSGIMIAVLIVVGTVGGGLAYYF	IKRANK
SS50377_18065	CRMP-2	18.1	11	6	1	VAGIVIAVLVVIGGIGGGVFWYL	KKSKSQLKNVSQQEIMQE
SS50377_19026	CRMP-2	22.2	11	3	3	PIIATIIAIIPFLFALSPCPNNF	KKHQFTCKLNDCESKCNGGGCRYDPVNHKFSCVCKIHFEGQYCENCEEGYLYQSDTK- DCELKSCVNGKYQNGKCICKDNYSGDYCENCKGQIYQNKCILNIVCNEKQKQVFSGG- QFSCQCNNGFTGNKCQKCEKGKVVVDNICVQK
SS50377_10026	CRMP-2	13.3	10	2	1	GIFGGCLILAVIIGVSVVICSII	KKKKQANADYLEVPLQADDVI
SS50377_10861	CRMP-2	21.4	10	3	2	AIAGIVIGVLALLALIFLIVFVA	MRRKPTGPSKAKSSVTKDTVGGQRFI
SS50377_10889	CRMP-2	23.7	10	6	1	AISGIVIGSLLLLLLLILLIIYI	IWKNRKQKIEEEEIVQLNIAYQNDDSVLMDVK
SS50377_11861	CRMP-2	11.8	10	2	1	ISGVILGCLLGIVILLITILVTV	KISLRKRKSNHIRLV
SS50377_17485	CRMP-2	13.3	10	4	1	VLGISLAVKSVVLGASGGVFWYL	RRAKIAKRKTVMKWRQQ
SS50377_10357	CRMP-2	216.1	9	8	2	IVIGFLVIFILCASGIYLWLILA	KNRRNLIVSFVDYRVKGIIISKDDQQTSYSGFLSGTEDRIFELA
SS50377_10551	CRMP-2	7.6	9	2	1	YLSVFIAAAITLSIAIVLYQYRP	QTD
SS50377_11695	CRMP-2	86.9	9	1	27	IGVGTVVGVLCGVLIALGVYFLV	KKYQQRPKIAAKTDLPKVQKVKKAKKAVKAFGRVQVSKPQME
SS50377_12210	CRMP-2	17.9	9	3	0	ISFAAVFTIYSFICFLCIIVIFR	KQINGKINEKVISGVIAKPVLVQLL
SS50377_12576	CRMP-2	44.1	9	11	1	IFFTHIIVDKKVFALILVVLIFL	INLK
SS50377_12824	CRMP-2	23.0	9	4	2	IVAVTILVIIFVLHAIGITWYIV	NKCYKNAVLKSQGIKSA
SS50377_14197	CRMP-2	14.2	9	3	1	TGGIIGIVIAILVAVGVVGGVVG	CCMRKKKHTAVVADAETVVAGQQ
SS50377_14248	CRMP-2	42.7	9	7	0	LIISLCIIVVLAIVIAYSATFIL	KKVRYNKLLKAALLAALSDEDKKMVEEEQAFDVNDYMTTVQEIPWEDEI
SS50377_16084	CRMP-2	52.0	9	16	0	IVGIVVGVVAAIGLVAGAGAYFI	VKKRKQAVVSPPKDDEIKHANFQVLISSMENNKIYNDLYY

ID	Group	MW	Cys%	CXXC	CXC	Transmembrane	C-terminal sequence
SS50377_16112	CRMP-2	20.2	9	1	2	ALSVLVLYAVFTSLIAGLKCFML	GPAENQQSHSGRFSCWRPCSPRSSCLAPRRRSPEPSRRAWCSTSRCSSWWSASTTPSWGARCRASPASRCSRATSSACRPCSPARCQSRARRPPPTRSPRFCWPSEGTSSTGCCCRRSSSARSGWRSCWC
SS50377_17858	CRMP-2	39.2	9	6	6	LIVSVSAGILAIIILIIVIVCLF	RKGILPIYRTKQVEARQKNVIQDQPQYQPIYPTVPTIPTRNGQIIFTADMKVVQV
SS50377_18255	CRMP-2	10.9	9	3	0	TLYIIIGVSIGVIFSVIAIISIF	CYFKKPKQGIENEPLLQEIDE
SS50377_12343	CRMP-2	19.0	8	3	2	IALWMLGIIISVQILILSYFVTV	KLVQMRKVKLQKLSNE
SS50377_12931	CRMP-2	60.7	8	4	0	VDGAVAIGVVLGLIGVIGALGNL	V
SS50377_15458	CRMP-2	15.2	8	3	1	AGIIVSVIAVGGAVGGGLAYYFI	RKSKLAIARQ
SS50377_16922	CRMP-2	38.0	8	4	3	IVSTVVGGVLLLVAGAVVFICIW	KAHAQKRDFLAAASPPLCTDQGMHI
SS50377_16927	CRMP-2	38.0	8	4	3	IVSTVVGGVLLLVAGAVVFICIW	KAHAQKRDFLAAASPPLCTDQGMHI
SS50377_17449	CRMP-2	11.0	8	2	3	GTILLLIVASIYSCCCCCFACCC	RKSVVQSKNKPNNANQAQYVMVSPLQEVPATANNGEGLLSSH
SS50377_18252	CRMP-2	11.5	8	3	0	LIGILSAGVIAVIIIIAGIIISF	RLIQKKKADKERQPLVDAEQQNELI
SS50377_18254	CRMP-2	11.8	8	3	0	IIGISMGVVFFLVIIFIAAFVIV	KSKKQSAASKLEKEPLLQEKQLEQQ
SS50377_14538	CRMP-2	16.2	7	2	1	LSGGAVAGIVIAVLVVYAQFEVA	MYITSSRRPRSEKYFHFSHQYLLLQYGYI
SS50377_14715	CRMP-2	16.3	7	2	1	LSGGAVAGIVVAVLVVYAQFEVA	LHITSSRRPRSEKYFHFSHQYLLLLYRYI
SS50377_18243	CRMP-2	13.7	7	3	0	IIFIVAVTVLPLMFIVFSILFVI	KVIIELKDKTIKKDNEDLLLLTDKDSISAEAKIGEYAYNYD
SS50377_16266	CRMP-2	113.2	6	4	6	ATISVIFSLLAIGIAVFLIVFFV	RRARNSKVKASRMTVTGKSVARYFVE
SS50377_16420	CRMP-2	27.3	6	3	1	IAGIAVGSILVIGIAVGTVCYFL	KRKSRFAQNQTQAVKDTTMDTIFGGQTVMRSQ
SS50377_13382	CRMP-2	56.8	5	4	2	LSFFVLILIIFIVVLIMFFVVLF	RTKMPKFLTHKPDRNVHNDGGLNENGKKGVSLNVILEQASQMQQ
SS50377_18863	CRMP-2	29.3	5	2	1	IAPTTLAIGVTIAVCFVTSLTVY	FPHYRKELYVSFAVAPTCMRALCRYNRKQQEVVYGQGSPNYWGSRLGRREIPAIHFTGII
SS50377_16923	CRP	7.9	36	9	13		
SS50377_19119	CRP	8.4	21	5	3		
SS50377_14551	CRP	10.9	18	9	0		
SS50377_17375	CRP	45.0	18	31	3		
SS50377_13213	CRP	22.9	17	10	2		
SS50377_13251	CRP	17.5	17	9	1		
SS50377_16087	CRP	20.3	17	15	0		
SS50377_16885	CRP	47.9	17	33	3		
SS50377_17177	CRP	20.8	17	9	2		
SS50377_17382	CRP	55.8	17	32	5		
SS50377_17481	CRP	23.2	17	16	1		
SS50377_12851	CRP	13.7	16	6	1		
SS50377_13211	CRP	13.7	16	6	1		
SS50377_14678	CRP	61.1	16	37	4		

ID	Group	MW	Cys%	CXXC	CXC	Transmembrane	C-terminal sequence
SS50377_15973	CRP	65.7	16	24	8		
SS50377_17745	CRP	12.2	16	6	1		
SS50377_12057	CRP	31.0	15	11	3		
SS50377_13273	CRP	82.5	15	30	10		
SS50377_18958	CRP	6.5	15	2	1		
SS50377_ja002	CRP	6.4	15	2	1		
SS50377_12056	CRP	44.7	14	12	4		
SS50377_12545	CRP	59.2	14	31	0		
SS50377_16021	CRP	13.7	14	2	2		
SS50377_16108	CRP	133.6	14	44	13		
SS50377_10550	CRP	6.6	13	1	0	1*	
SS50377_10705	CRP	47.9	13	15	3		
SS50377_14913	CRP	58.3	13	18	2		
SS50377_16132	CRP	7.9	13	4	1		
SS50377_18098	CRP	24.3	13	8	2		
SS50377_18843	CRP	34.4	13	12	2		
SS50377_ja001	CRP	30.5	13	15	0		
SS50377_jh039	CRP	19.5	12	6	0		
SS50377_10836	CRP	11.4	12	1	0	1*	
SS50377_13236	CRP	36.9	12	13	1		
SS50377_jh077	CRP	19.6	12	6	0		
SS50377_15348	CRP	8.4	12	1	1		
SS50377_15470	CRP	12.7	12	3	1		
SS50377_fx068	CRP	12.0	11	3	2		
SS50377_13111	CRP	9.2	11	1	2		
SS50377_ee044	CRP	12.0	11	3	2		
SS50377_14002	CRP	21.1	11	8	0		
SS50377_14257	CRP	16.3	11	3	1		
SS50377_14704	CRP	38.9	11	13	1		
SS50377_14837	CRP	6.2	11	1	0		
SS50377_15373	CRP	53.4	11	15	1		
SS50377_15972	CRP	27.0	11	6	2		
SS50377_18295	CRP	18.0	11	6	0		
SS50377_18560	CRP	11.7	11	4	1		

ID	Group	MW	Cys%	CXXC	CXC	Transmembrane	C-terminal sequence
SS50377_13471	CRP	16.2	10	4	2		
SS50377_15383	CRP	19.2	10	3	2		
SS50377_18516	CRP	6.8	10	2	0		
SS50377_18721	CRP	19.2	10	3	2		

*) These proteins have a single TM domain but are classified as CRP because they do not fulfill the CRMP-2 criteria for CXC and CXXC.
