# Supplementary material for: The Genome of Spironucleus salmonicida Highlights a Fish Pathogen Adapted to Fluctuating Environments
Source: PLoS Genet. 2014 Feb 6;10(2):e1004053. doi: 10.1371/journal.pgen.1004053 (PMC3916229; doi:10.1371/journal.pgen.1004053)
Supplement: Table S5 — Identified transporter families in G. intestinalis and S. salmonicida. (PDF) [file pgen.1004053.s015.pdf]

Table S5 Identified transporter families in *G. intestinalis* and *S. salmonicida*.

Family	Short Name	Name	*SS*	*GI*
2.A.1	MFS	Major Facilitator Superfamily	46	17
3.A.1	ABC	ATP-binding Cassette Superfamily	21	26
2.A.18	AAAP	Amino Acid/Auxin Permease Family	28	11
9.B.37	HIP14	Huntington-interacting Protein 14 Family	20	9
2.A.7	DMT	Drug/Metabolite Transporter Superfamily	14	8
3.A.3	P-ATPase	P-type ATPase Superfamily	8	13
2.A.66	MOP	Multidrug/Oligosaccharidyl-lipid/Polysaccharide Flippase Superfamily	10	9
3.A.2	F-ATPase	H+- or Na+-translocating F-type, V-type and A-type Superfamily	5	4
2.A.57	ENT	Equilibrative Nucleoside Transporter Family	7	1
2.A.43	LCT	Lysosomal Cystine Transporter Family	5	2
1.B.12	AT-1	Autotransporter-1 Family	3	3
2.A.5	Zn2+	Zinc (Zn2+)-Iron (Fe2+) Permease (ZIP) Family	5	1
2.A.4	CDF	Cation Diffusion Facilitator Family	3	2
1.A.1	VIC	Voltage-gated Ion Channel Superfamily	4	1
8.A.28	Ankyrin	Ankyrin Family	1	4
3.A.16	ER-RT	Endoplasmic Reticular Retrotranslocon Family	2	2
1.A.35	MIT	CorA Metal Ion Transporter Family	2	2
5.A.1	DsbD	Disulfide Bond Oxidoreductase D Family	2	2
2.A.92	CTL	Choline Transporter-like Family	4	0
8.A.27	PLI-	Phospholipid Importer -subunit Family	2	1
2.A.36	CPA1	Monovalent Cation:Proton Antiporter-1 Family	2	1
9.A.54	B12	Lysosomal Cobalamin (B12) Transporter Family	1	2
9.A.40	HCC	HlyC/CorC Family	2	1
2.A.69	AEC	Auxin Efflux Carrier Family	2	1
3.A.5	Sec	General Secretory Pathway Family	2	1
4.C.1	FAT	Proposed Fatty Acid Transporter Family	3	0
9.B.30	Hly III	Hly III Family	3	0
2.A.3	APC	Amino Acid-Polyamine-Organocation Family	1	1
3.A.20	PPI	Peroxisomal Protein Importer Family	2	0
9.B.142	gt39	integral membrane glycosyltransferase family 39 family	1	1
8.A.21	Stomatin	Stomatin/Podocin/Band 7/Nephrosis.2/SPFH Family	2	0
8.A.30	Nedd4	Nedd4-Family Interacting Protein-2 Family	2	0
9.B.82	Rer1	Endoplasmic Reticulum Retrieval Protein1 (Putative Heavy Metal Transporter) Family	1	1
2.A.94	Pho1	Phosphate Permease Family	1	1
1.A.54	Presenilin	Presenilin ER Ca2+ Leak Channel Family	0	1
1.A.17	Ca-ClC	Calcium-Dependent Chloride Channel Family	0	1
3.A.18	mRNA-E	Nuclear mRNA Exporter Family	0	1
9.B.1	MHP	Metal Homeostasis Protein Family	0	1
2.A.48	RFC	Reduced Folate Carrier Family	0	1
1.A.33	Hsp70	Cation Channel-forming Heat Shock Protein-70 Family	0	1
9.A.6	ATP-E	ATP Exporter Family	0	1
2.A.2	GPH	Glycoside-Pentoside-Hexuronide:Cation Symporter Family	1	0
2.A.12	AAA	ATP:ADP Antiporter Family	0	1
9.B.77	Meckelin	Meckel Syndrome Protein Family	1	0
3.D.1	NDH	H+ or Na+-translocating NADH Dehydrogenase Family	0	1
9.A.45	MagT1	Magnesium Transporter1 Family	0	1
