# Supplementary material for: The Genome of Spironucleus salmonicida Highlights a Fish Pathogen Adapted to Fluctuating Environments
Source: PLoS Genet. 2014 Feb 6;10(2):e1004053. doi: 10.1371/journal.pgen.1004053 (PMC3916229; doi:10.1371/journal.pgen.1004053)
Supplement: Figure S7 — Alignment of the CRMP-1 motif. MUSCLE v3.8.31 [97] was used to align 125 CRMP-1 complete amino acid sequences. Jalview v14.0 [101] was used to visualize and filter the alignment. Left part (2034 positions) of the alignment was removed leaving only C-terminus conserved region (79 positions). There are 66 sequences left after removing redundancy with threshold 95. (PDF) [file pgen.1004053.s007.pdf]

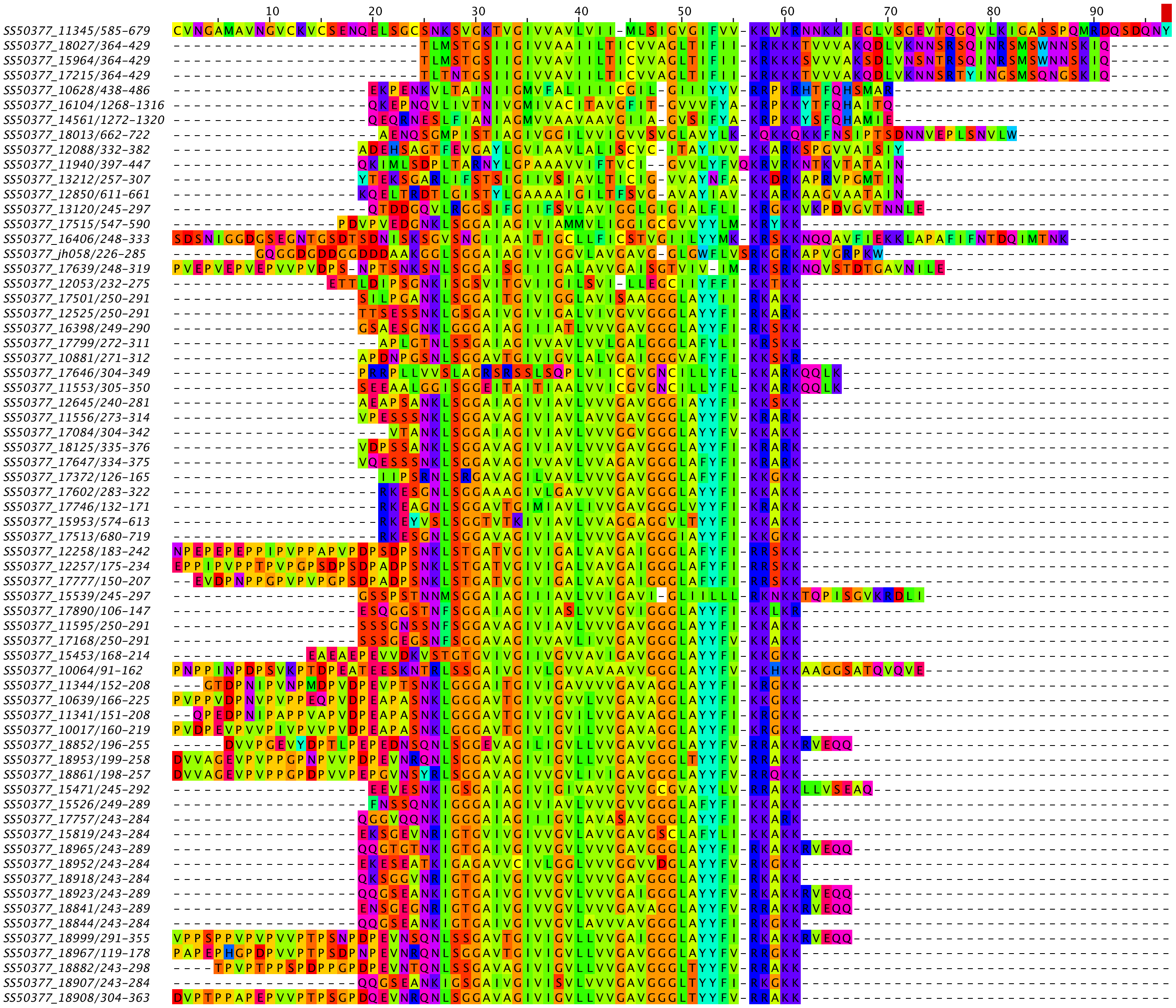
10
20
30
40
50
60
70
80
90
SS50377_11345/585-679
SS50377_18027/364-429
SS50377_15964/364-429
SS50377_17215/364-429
SS50377_10628/438-486
SS50377_16104/1268-1316
SS50377_14561/1272-1320
SS50377_18013/662-722
SS50377_12088/332-382
SS50377_11940/397-447
SS50377_13212/257-307
SS50377_12850/611-661
SS50377_13120/245-297
SS50377_17515/547-590
SS50377_16406/248-333
SS50377_jh058/226-285
SS50377_17639/248-319
SS50377_12053/232-275
SS50377_17501/250-291
SS50377_12525/250-291
SS50377_16398/249-290
SS50377_17799/272-311
SS50377_10881/271-312
SS50377_17646/304-349
SS50377_11553/305-350
SS50377_12645/240-281
SS50377_11556/273-314
SS50377_17084/304-342
SS50377_18125/335-376
SS50377_17647/334-375
SS50377_17372/126-165
SS50377_17602/283-322
SS50377_17746/132-171
SS50377_15953/574-613
SS50377_17513/680-719
SS50377_12258/183-242
SS50377_12257/175-234
SS50377_17777/150-207
SS50377_15539/245-297
SS50377_17890/106-147
SS50377_11595/250-291
SS50377_17168/250-291
SS50377_15453/168-214
SS50377_10064/91-162
SS50377_11344/152-208
SS50377_10639/166-225
SS50377_11341/151-208
SS50377_10017/160-219
SS50377_18852/196-255
SS50377_18953/199-258
SS50377_18861/198-257
SS50377_15471/245-292
SS50377_15526/249-289
SS50377_17757/243-284
SS50377_15819/243-284
SS50377_18965/243-289
SS50377_18952/243-284
SS50377_18918/243-284
SS50377_18923/243-289
SS50377_18841/243-289
SS50377_18844/243-284
SS50377_18999/291-355
SS50377_18967/119-178
SS50377_18882/243-298
SS50377_18907/243-284
SS50377_18908/304-363
